# Attachment of Chiral Functional Groups to Modify the Activity of New GPx Mimetics

**DOI:** 10.3390/ma15062068

**Published:** 2022-03-11

**Authors:** Anna Laskowska, Agata Joanna Pacuła-Miszewska, Angelika Długosz-Pokorska, Anna Janecka, Andrzej Wojtczak, Jacek Ścianowski

**Affiliations:** 1 Department of Organic Chemistry, Faculty of Chemistry, Nicolaus Copernicus University, 7 Gagarin Street, 87-100 Torun, Poland; annlas@doktorant.umk.pl (A.L.); pacula@umk.pl (A.J.P.-M.); 2 Department of Biomolecular Chemistry, Faculty of Medicine, Medical University of Lodz, Mazowiecka 6/8, 92-215 Lodz, Poland; angelika.dlugosz@umed.lodz.pl (A.D.-P.); anna.janecka@umed.lodz.pl (A.J.); 3 Department of Crystallochemistry and Biocrystallography, Faculty of Chemistry, Nicolaus Copernicus University in Torun, 7 Gagarin Street, 87-100 Torun, Poland; awojt@umk.pl

**Keywords:** benzisoselenazol-3(2*H*)-ones, diselenides, pharmacophore, antioxidant activity, antiproliferative activity

## Abstract

A series of new chiral benzisoselenazol-3(2*H*)-ones and their corresponding diselenides bearing an *o*-amido function substituted on the nitrogen atom with various aliphatic and aromatic moieties were synthesized. All derivatives representing pairs of enantiomers or diastereoisomers were obtained to thoroughly evaluate the three-dimensional structure–activity correlation. First, bensisoselenazol-3(2*H*)-ones were synthesized by reacting 2-(chloroseleno)benzoyl chloride with an appropriate enantiomerically pure amine. Then, the Se–N bond was cleaved by a reduction–oxidation procedure using sodium borohydride and then air oxidation to obtain the corresponding diselenides. All derivatives were tested as antioxidants and anticancer agents. In general, the diselenides were more reactive peroxide scavengers, with the highest activity observed for 2,2′-diselenobis[*N*-(1*S*,2*S*)-(-)-*trans*-2-hydroksy-1-indanylbezamide]. The most cytotoxic derivative towards human promyelocytic leukemia HL-60 and breast cancer MCF-7 cell lines was *N*-[(1*S*,2*R*)-(-)-*cis*-2-hydroksy-1-indanyl]-1,2-benzizoselenazol-3(2*H*)-one. The structure–activity relationship of the obtained organoselenium derivatives was discussed.

## 1. Introduction

As the human body is a combination of permanent changes in concentration, tension and movement, the overall biochemical processes that occur are far from maintaining equilibrium. All the reactions and transformation taking place, in most cases carried out by asymmetrical molecules—irregular yet perfectly fitted to one another—create an excellently designed system in this seemingly chaotic phenomenon. The key element which is crucial for proper enzyme function, structure and cell metabolism is the homochirality of L-amino acids and D-sugars. These small molecules impose the chirality of more complex structures such as proteins and nucleic acids, which subsequently force the chirality of the whole cell [1,2,3]. As a result, potential drugs generally possess fixed stereocenters to efficiently interact with specific receptors that have a characteristic spatial structure at their binding side [4,5,6].

Additionally, the primary biochemical role of selenium is associated with its activity as the chiral amino acid L-selenocysteine that forms the active center of the antioxidant enzyme glutathione peroxidase. This enables the elimination of excessive reactive oxygen species production and redox homeostasis preservation [7,8]. The chiral amino acid environment of GPx, which surrounds and interacts with L-Sec, is essential for stabilizing L-selenocysteine in its active-selenol form **1** [9]. As can thus be deduced, the spatial structure of the bioactive molecule is an important feature that influences the activity of the enzyme and the entire biochemical cycle. The same assumption can be superimposed on drugs, where one enantiomer can be a potential therapeutic with the other exhibiting significant toxicity, as in the well-known case of (*R*)- and (*S*)-thalidomide [10].

**Scheme 1:**
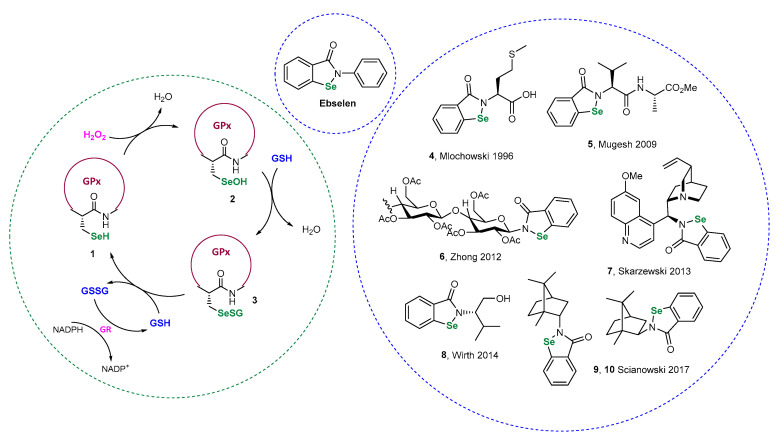
GPx activity cycle and the structure of known chiral benzisoselenazolones **4**–**10**.

To date, a multitude of GPx mimetics possessing an organoselenium moiety that imitates the characteristic activity of *L*-Sec was presented and proved efficient in both in vitro and in vivo assays [11,12]. Among them, *N*-substituted benzisoselenazolones, represented by the versatile potential selenotherapeutic Ebselen, create the most explored group of derivatives with several chiral examples, including functionalized amino acids **4** and **5** [13,14], sugars **6** [15], alkaloids **7** [16], alcohols **8** [17] and terpenes **9** and **10** [18] (Scheme 1).

**Figure 1:**
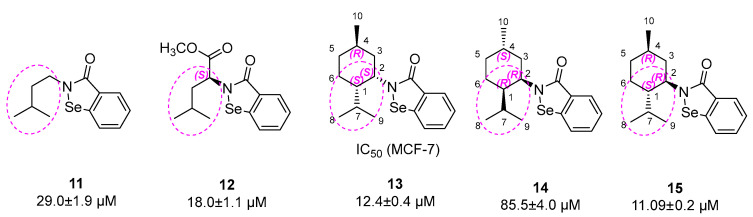
Structural elements influencing the reactivity of compounds **11**–**15**.

However, the difference in the biological activity of GPx mimetics, possessing distinct 3-dimensional orientation of atoms that serve to compare their epimeric/enantiomeric forms, has hardly been evaluated but was originally presented by our research group. Using enantiomerically pure terpene amines from *p*-menthane, carane and pinane systems, a series of *N*-terpenylbenzisoselenazolo-3(2*H*)-ones including pairs of enantiomers and diastereoisomers, were synthetized [19]. In our previous studies, we observed that antiproliferative activity towards the breast cancer cell line MCF-7 was increased by the presence of a 2-methylbuthyl carbon chain (structures **11** and **12**) [18]. The most potent terpene derivatives **13** and **15** confirmed this assumption. However, a significantly different result was obtained for the enantiomer of compounds **13**–**14** which envisioned the chirality of C1 and C4 carbon centers as the key element influencing reactivity, and that the difference in activity between the two enantiomers may be significant (Figure 1).

**Figure 2:**
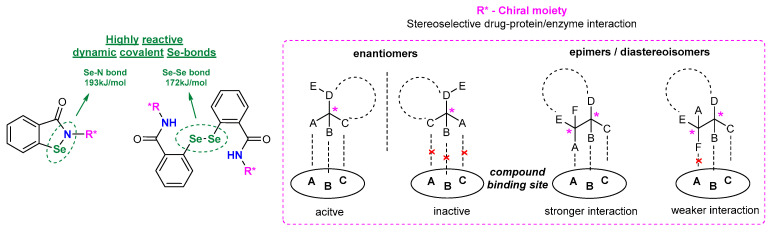
Structural features of the designed compounds—interaction with the target binding sites.

Herein, we planned to address this issue and identify the particular structural features that affect the biological activity of organoselenium GPx mimetics. The opposite enantiomers/diastereoisomers can exhibit stronger or weaker activity due to the change of interaction or affinity induction to other target domains. The biological activity evaluation will show whether the configuration of particular carbon centers can modulate the reactivity of the molecules. The chiral organoselenium compounds will also be obtained in two forms—the aforementioned *N*-substituted benzisoselenazol-3(2*H*)-ones, with a reactive Se–N bond (193 kJ/mol), and the corresponding diselenides, possessing a Se–Se functionality with lower bond energy (172 kJ/mol) (Figure 2) [20,21]. 

As presented in Scheme 1, the reactive selenol of GPx has to covalently bind with inter- or intracellular thiols to regenerate the primary reactive form. The ease of breaking the Se-bond determines the hydrogen peroxide reduction and enzyme regeneration rate. This way, the synthesis of the presented compounds will enable recognizing the influence of the chiral moiety and the reactivity of two different types of organoselenium groups on the bioactivity of the GPx mimetics.

## 2. Materials and Methods

### 2.1. General

NMR spectra were recorded on Bruker Avance III/400 or Bruker Avance III/700 (Karlsruhe, Germany) for 1H and 176.1 MHz or 100.6 MHz for 13 C (see Supplementary Materials). Chemical shifts were recorded relative to SiMe_4_ (δ0.00) or solvent resonance (CDCl_3_ δ7.26, CD3OD δ3.31). Multiplicities were given as: s (singlet); d (doublet); dd (double doublet); ddd (double doublet); t (triplet); dt (double triplet); and m (multiplet). The 77Se NMR spectra were recorded on Bruker Avance III/400 or Bruker Avance III/700 with diphenyl diselenide as an external standard. NMR spectra were carried out using the ACD/NMR Processor Academic Edition. Melting points were measured with a Büchi Tottoli SPM-20 heating unit (Büchi Labortechnik AG, Flawil, Switzerland) and were uncorrected. Elemental analyses were performed on a Vario MACRO CHN analyzer (Elementar Analysensysteme GmbH, Langensenbold, Germany). Optical rotations were measured in 10 mm cells with a polAAr 3000 polarimeter (Optical Activity Limited, Ramsey, United Kingdom). Column chromatography was performed using Merck 40-63D 60 Å silica gel (Merck, Darmstadt, Germany).

### 2.2. Procedures and Analysis Data

Compounds were synthesized according to the previously presented procedure [18].

*N*-[(*S*)-(+)-*sec*-butyl]-1,2-benzisoselenazol-3(2*H*)-one **18a**

Yield: 85%; mp 51–52 °C; ${\left[\propto \right]}_{D}^{20}$ = +40 (c = 1.12, CHCl_3_);

^1^H NMR (700 MHz, DMSO) δ = 0.84 (t, J = 7.0 Hz, 3H), 1.26 (d, J = 7.0 Hz, 3H), 1.58–1.69 (m, 2H), 4.39–4.44 (m, 1H), 7.41–7.44 (m, 1H_ar_), 7.58–7.62 (m, 1H_ar_), 7.79–7.82 (m, 1H_ar_), 8.03–8.06 (m, 1H_ar_) ^13^C NMR (700 MHz, DMSO) δ = 11.40 (CH_3_), 21.74 (CH_3_), 30.57 (CH_2_), 51.31 (CH), 126.24 (2xCH_ar_), 127.78 (CH_ar_), 129.26 (C_ar_), 131.74 (CH_ar_), 139.22 (C_ar_), 166.67 (C=O) ^77^Se NMR 700 MHz, DMSO) δ = 804.33 ppm; IR = 2964, 2873, 1738, 1589, 1562, 1443, 1323, 1247, 1217, 1147, 1046, 1020, 788, 737, 697, 591, 509, 416 cm^−1^. Elemental Anal. Calcd for C_11_H_13_NOSe (255.02): C, 51.98; H, 5.15; N, 5.51; Found C, 51.93; H, 5.15; N, 5.49.

*N*-[(*R*)-(−)-*sec*-butyl]-1,2-benzisoselenazol-3(2*H*)-one **19a**

Yield: 67%; mp 50–52 °C; ${\left[\propto \right]}_{D}^{20}$ = −38 (c = 1.24, CHCl_3_);

^1^H NMR (700 MHz, DMSO) δ = 0.84 (t, J = 7.0 Hz, 3H), 1.26 (d, J = 6.3 Hz, 3H), 1.57–1.69 (m, 2H), 4.39–4.44 (m, 1H), 7.40–7.44 (m, 1H_ar_), 7.58–7.62 (m, 1H_ar_), 7.79–7.82 (m, 1H_ar_), 8.03–8.06 (m, 1H_ar_) ^13^C NMR (400 MHz, DMSO) δ = 11.42 (CH_3_), 21.77 (CH_3_), 30.57 (CH_2_), 51.26 (CH), 126.22 (CH_ar_), 126.27 (CH_ar_), 127.76 (CH_ar_), 129.28 (C_ar_), 131.71 (CH_ar_), 139.25 (C_ar_), 166.64 (C=O) ^77^Se NMR (400 MHz, DMSO) δ = 802.56 ppm; IR = 2965, 2873, 1737, 1589, 1563, 1443, 1323, 1246, 1216, 1148, 1047, 1020, 788, 737, 697, 592, 508, 414 cm^−1^. Elemental Anal. Calcd for C_11_H_13_NOSe (255.02): C, 51.98; H, 5.15; N, 5.51; Found C, 51.95; H, 5.16; N, 5.47.

*N*-[(*S*)-(+)-1-hydroxy-2-butanyl]-1,2-benzisoselenazol-3(2*H*)-one **20a**

Yield: 45%; mp 108–109 °C; ${\left[\propto \right]}_{D}^{20}$ = +47 (c = 0.47, CHCl_3_);

^1^H NMR (700 MHz, DMSO) δ = 0.81 (t, J = 7.7 Hz, 3H), 1.53–1.60 (m, 1H), 1.71–1.77 (m, 1H), 3.52–3.56 (m, 1H), 3.64–3.68 (m, 1H), 4.40–4.44 (m, 1H), 5.14 (t, J = 5.6 Hz, 1H), 7.39–7.42 (m, 1H_ar_), 7.53–7.61 (m, 1H_ar_), 7.81 (dd, J_1_ = 1.4 Hz, J_2_ = 7.0 Hz, 1H_ar_), 8.01–8.04 (m, 1H_ar_) ^13^C NMR (700 MHz, DMSO) δ = 11.09 (CH_3_), 24.98 (CH_2_), 56.94 (CH), 63.77 (CH_2_), 125.96 (2xCH_ar_), 127.72 (CH_ar_), 128.58 (C_ar_), 131.63 (CH_ar_), 140.55 (C_ar_), 167.32 (C=O) ^77^Se NMR (700 MHz, DMSO) δ = 839.29 ppm; IR = 3224, 2959, 2871, 1590, 1562, 1444, 1338, 1310, 1252, 1082, 1020, 791, 739, 676, 601, 549, 483, 422 cm^−1^. Elemental Anal. Calcd for C_11_H_13_NO_2_Se (271.01): C, 48.90; H, 4.85; N, 5.18; Found C, 48.92; H, 4.85; N, 5.11.

*N*-[(*R*)-(−)-1-hydroxy-2-butanyl]-1,2-benzisoselenazol-3(2*H*)-one **21a**

Yield: 57%; mp 109–110 °C; ${\left[\propto \right]}_{D}^{20}$ = −41 (c = 0.83, CHCl_3_);

^1^H NMR (700 MHz, DMSO) δ = 0.81 (t, J = 7.7 Hz, 3H), 1.53–1.60 (m, 1H), 1.71–1.77 (m, 1H), 3.51–3.57 (m, 1H), 3.64–3.68 (m, 1H), 4.38–4.44 (m, 1H), 5.12 (t, J = 4.9 Hz, 1H), 7.39-7.42 (m, 1H_ar_), 7.57–7.61 (m, 1H_ar_), 7.80–7.83 (m, 1H_ar_), 8.02 (d, J = 7.7 Hz, 1H_ar_) ^13^C NMR (400 MHz, DMSO) δ = 11.10 (CH_3_), 24.96 (CH_2_), 56.87 (CH), 63.73 (CH_2_), 125.96 (CH_ar_), 125.98 (CH_ar_), 127.70 (CH_ar_), 128.57 (C_ar_), 131.63 (CH_ar_), 140.56 (C_ar_), 167.30 (C=O) ^77^Se NMR (400 MHz, DMSO) δ = 837.92 ppm; IR = 3233, 2959, 2871, 1591, 1562, 1444, 1338, 1310, 1252, 1081, 1020, 791, 741, 676, 600, 549, 484, 421 cm^−1^. Elemental Anal. Calcd for C_11_H_13_NO_2_Se (271.01): C, 48.90; H, 4.85; N, 5.18; Found C, 48.93; H, 4.84; N, 5.18.

*N*-[(*R*)-(−)-1,2,3,4-tetrahydro-1-napthyl]-1,2-benzisoselenazol-3(2*H*)-one **22a**

Yield: 96%; mp 217–220 °C; ${\left[\propto \right]}_{D}^{20}$ = −50 (c = 0.58, CHCl_3_) (lit. mp 212–214 °C; ${\left[\propto \right]}_{D}^{20}$ = −0.53 (c = 0.075, CHCl_3_) [17])

^1^H NMR (700 MHz, DMSO) δ = 1.81–1.87 (m, 1H), 1.95–2.05 (m, 2H), 2.09–2.15 (m, 1H), 2.76–2.81 (m, 1H), 2.87–2.92 (m, 1H), 5.60 (t, J = 7 Hz, 1H), 7.02–7.06 (m, 1H_ar_), 7.13–7.25 (m, 3H_ar_), 7.43–7.47 (m, 1H_ar_), 7.59–7.62 (m, 1H_ar_), 7.84–7.89 (m, 1H_ar_), 7.96 (d, J = 7.7 Hz, 1H) ^13^C NMR (400 MHz, DMSO) δ = 20.57 (CH_2_), 29.16 (CH_2_), 30.77 (CH_2_), 51.93 (CH), 126.05 (CH_ar_), 126.32 (CH_ar_), 126.48 (CH_ar_), 127.86 (CH_ar_), 128.13 (CH_ar_), 128.75 (CH_ar_), 129.40 (CH_ar_), 129.50 (CH_ar_), 131.95 (C_ar_), 136.21 (C_ar_), 138.12 (C_ar_), 139.77 (C_ar_), 166.88 (C=O) ^77^Se NMR (400 MHz, DMSO) δ = 830.37 ppm; IR = 2919, 2857, 1588, 1561, 1491, 1441, 1308, 1268, 1245, 1154, 1082, 769, 733, 677, 572, 534, 511, 481, 418 cm^−1^. Elemental Anal. Calcd for C_17_H_15_NOSe (329.03): C, 62.20; H, 4.61; N, 4.27; Found C, 62.25; H, 4.62; N, 4.29.

*N*-[(*S*)-(+)-1,2,3,4-tetrahydro-1-napthyl]-1,2-benzisoselenazol-3(2*H*)-one **23a**

Yield: 94%; mp 218–220 °C; ${\left[\propto \right]}_{D}^{20}$ = +54 (c = 0.42, CHCl_3_);

^1^H NMR (700 MHz, DMSO) δ = 1.77–1.84 (m, 1H), 1.92–2.02 (m, 2H), 2.06–2.12 (m, 1H), 2.72–2.79 (m, 1H), 2.83–2.89 (m, 1H), 5.57 (t, J = 7 Hz, 1H), 7.00–7.03 (m, 1H_ar_), 7.10–7.22 (m, 3H_ar_), 7.40–7.44 (m, 1H_ar_), 7.55–7.59 (m, 1H_ar_), 7.84–7.86 (m, 1H_ar_), 7.93 (d, J = 7.7 Hz, 1H) ^13^C NMR (400 MHz, DMSO) δ = 20.58 (CH_2_), 29.16 (CH_2_), 30.77 (CH_2_), 51.92 (CH), 126.06 (CH_ar_), 126.31 (CH_ar_), 126.47 (CH_ar_), 127.85 (CH_ar_), 128.11 (CH_ar_), 128.76 (CH_ar_), 129.40 (CH_ar_), 129.50 (CH_ar_), 131.93 (C_ar_), 136.22 (C_ar_), 138.12 (C_ar_), 139.77 (C_ar_), 166.86 (C=O) ^77^Se NMR (400 MHz, DMSO) δ = 830.01 ppm; IR = 2921, 2853, 1589, 1561, 1492, 1441, 1308, 1269, 1245, 1154, 1081, 769, 734, 676, 572, 534, 512, 481, 419 cm^−1^. Elemental Anal. Calcd for C_17_H_15_NOSe (329.03): C, 62.20; H, 4.61; N, 4.27; Found C, 62.29; H, 4.62; N, 4.21.

*N*-[(*R*)-(+)-*α*-methylbenzyl]-1,2-benzisoselenazol-3(2*H*)-one **24a**

Yield: 49%; mp 108–110 °C; ${\left[\propto \right]}_{D}^{20}$ = +128 (c = 1.04, CHCl_3_) (lit. mp 116–117.5 °C; ${\left[\propto \right]}_{D}^{20}$ = +120 (c = 1.00, C_2_H_5_OH) [13])

^1^H NMR (700 MHz, DMSO) δ = 1.68 (d, J = 7 Hz, 3H), 5.67 (q, J = 7 Hz, 1H), 7.30–7.33 (m, 1H_ar_) 7.36–7.42 (m, 4H_ar_), 7.43–7.45 (m, 1H_ar_), 7.58–7.61 (m, 1H_ar_), 7.83 (dd, J_1_ = 0.7 Hz, J_2_ = 7.7 Hz, 1H_ar_), 7.99 (d, J = 7.7 Hz, 1H) ^13^C NMR (700 MHz, DMSO) δ = 20.86 (CH_3_), 52.37 (CH), 126.18 (CH_ar_), 126.32 (CH_ar_), 127.40 (2xCH_ar_), 127.83 (CH_ar_), 128.11 (CH_ar_), 128.99 (2xCH_ar_), 129.03 (C_ar_), 131.88 (CH_ar_), 139.58 (C_ar_), 142.61 (C_ar_), 166.34 (C=O) ^77^Se NMR (700 MHz, DMSO) δ = 819.68 ppm; IR = 2921, 1590, 1560, 1492, 1441, 1308, 1249, 1154, 1115, 1059, 759, 738, 695, 610, 562, 538, 504, 480, 420 cm^−1^. Elemental Anal. Calcd for C_15_H_13_NOSe (303.02): C, 59.61; H, 4.34; N, 4.63; Found C, 59.68; H, 4.35; N, 4.60.

*N*-[(*S*)-(−)-*α*-methylbenzyl]-1,2-benzisoselenazol-3(2*H*)-one **25a**

Yield: 95%; mp 109–111 °C; ${\left[\propto \right]}_{D}^{20}$ = −123 (c = 1.02, CHCl_3_) (lit. mp 116–117.5 °C; ${\left[\propto \right]}_{D}^{20}$ = −120 (c = 1.00, C_2_H_5_OH) [13])

^1^H NMR (700 MHz, DMSO) δ = 1.68 (d, J = 7 Hz, 3H), 5.67 (q, J = 7Hz, 1H), 7.30–7.33 (m, 1H_ar_) 7.37–7.42 (m, 4H_ar_), 7.42–7.45 (m, 1H_ar_), 7.58–7.61 (m, 1H_ar_), 7.83 (dd, J_1_ = 1.4 Hz, J_2_ = 7.7 Hz, 1H_ar_), 8.00 (d, J = 7.7 Hz, 1H) ^13^C NMR (700 MHz, DMSO) δ = 20.86 (CH_3_), 52.41 (CH), 126.18 (CH_ar_), 126.32 (CH_ar_), 127.40 (2xCH_ar_), 127.83 (CH_ar_), 128.11 (CH_ar_), 128.99 (2xCH_ar_), 129.02 (C_ar_), 131.89 (CH_ar_), 139.59 (C_ar_), 142.60 (C_ar_), 166.36 (C=O) ^77^Se NMR (700 MHz, DMSO) δ = 820.47 ppm; IR = 2924, 1590, 1561, 1492, 1441, 1307, 1245, 1158 1059, 759, 738, 695, 610, 560, 538, 504, 448, 416 cm^−1^. Elemental Anal. Calcd for C_15_H_13_NOSe (303.02): C, 59.61; H, 4.34; N, 4.63; Found C, 59.63; H, 4.34; N, 4.58.

*N*-[(*S*)-(−)-1-(1-napthyl)ethyl]-1,2-benzisoselenazol-3(2*H*)-one **26a**

Yield: 76%; mp 136–138 °C; ${\left[\propto \right]}_{D}^{20}$ = −241 (c = 0.72, CHCl_3_); 

^1^H NMR (700 MHz, DMSO) δ = 1.78 (d, J = 7.0 Hz, 3H), 6.37 (q, J = 6.3 Hz, 1H), 7.40–7.44 (m, 1H_ar_), 7.50–7.56 (m, 3H_ar_), 7.60–7.63 (m, 1H_ar_), 7.82 (d, J = 7 Hz, 1H_ar_), 7.84–7.89 (m, 2H_ar_), 7.94–8.02 (m, 3H_ar_) ^13^C NMR (700 MHz, DMSO) δ = 19,72 (CH_3_), 48,32 (CH), 123.37 (CH_ar_), 123.97 (CH_ar_), 125.67 (CH_ar_), 126.13 (CH_ar_), 126.30 (CH_ar_), 126.44 (CH_ar_), 127.29 (CH_ar_), 127.90 (CH_ar_), 129.00 (C_ar_), 129.21 (CH_ar_), 129.48 (CH_ar_), 131.63 (C_ar_), 131.82 (CH_ar_), 133.99 (C_ar_), 137.91 (C_ar_), 139.73 (C_ar_), 165.81 (C=O) ^77^Se NMR (700 MHz, DMSO) δ = 816.43 ppm; IR = 2924, 2853, 1590, 1562, 1442, 1341, 1309, 1245, 1079, 1020, 798, 736, 674, 605, 523, 505, 453 cm^−1^. Elemental Anal. Calcd for C_19_H_15_NOSe (353.03): C, 64.78; H, 4.29; N, 3.98; Found C, 64.82; H, 4.29; N, 3.90.

*N*-[(*R*)-(+)-1-(1-napthyl)ethyl]-1,2-benzisoselenazol-3(2*H*)-one **27a**

Yield: 65%; mp 135–137 °C; ${\left[\propto \right]}_{D}^{20}$ = +245 (c = 0.62, CHCl_3_) (lit. mp 130–132 °C; ${\left[\propto \right]}_{D}^{20}$ = +2.62 (c = 0.28, CHCl_3_) [17])

^1^H NMR (700 MHz, DMSO) δ = 1.78 (d, J = 7.7 Hz, 3H), 6.37 (q, J = 6.3 Hz, 1H), 7.40–7.44 (m, 1H_ar_), 7.50–7.56 (m, 3H_ar_), 7.60–7.63 (m, 1H_ar_), 7.82 (d, J = 7 Hz, 1H_ar_), 7.84–7.89 (m, 2H_ar_), 7.94–8.02 (m, 3H_ar_) ^13^C NMR (700 MHz, DMSO) δ = 19.74 (CH_3_), 48.29 (CH), 123.38 (CH_ar_), 123.96 (CH_ar_), 125.68 (CH_ar_), 126.14 (CH_ar_), 126.30 (CH_ar_), 126.44 (CH_ar_), 127.28 (CH_ar_), 127.88 (CH_ar_), 129.02 (C_ar_), 129.21 (CH_ar_), 129.46 (CH_ar_), 131.63 (C_ar_), 131.80 (CH_ar_), 133.99 (C_ar_), 137.94 (C_ar_), 139.73 (C_ar_), 165.77 (C=O) ^77^Se NMR (700 MHz, DMSO) δ = 815.51 ppm; IR = 2923, 2853, 1590, 1561, 1456, 1440, 1343, 1309, 1268, 1079, 1020, 797, 739, 675, 606, 522, 504, 453 cm^−1^. Elemental Anal. Calcd for C_19_H_15_NOSe (353.03): C, 64.78; H, 4.29; N, 3.98; Found C, 64.77; H, 4.30; N, 3.89.

*N*-[(1*S*,2*R*)-(−)-*cis*-2-hydroxy-1-indanyl]-1,2-benzisoselenazol-3(2*H*)-one **28a**

Yield: 71%; mp 187–189 °C; ${\left[\propto \right]}_{D}^{20}$ = −97 (c = 0.61, CHCl_3_);

^1^H NMR (700 MHz, DMSO) δ = 2.86–2.89 (m, 1H), 3.14–3.18 (m, 1H), 4.57–4.61 (m, 1H), 5.56 (d, J = 4.9 Hz, 1H), 5.95 (d, J = 4.9 Hz, 1H), 6.90 (d, J = 7.7 Hz, 1H_ar_), 7.14 (t, J = 7.0 Hz, 1H_ar_), 7.23 (t, J = 7.7 Hz, 1H_ar_), 7.28–7.31 (m, 1H_ar_), 7.38–7.41 (m, 1H_ar_), 7.55–7.59 (m, 1H_ar_), 7.87–7.90 (m, 1H_ar_), 7.95 (d, J = 7.7 Hz, 1H) ^13^C NMR (400 MHz, DMSO) δ = 39.52 (CH_2_), 61.36 (CH), 73.06 (CH), 124.73 (CH_ar_), 125.68 (CH_ar_), 125.71 (CH_ar_), 125.78 (CH_ar_), 126.91 (CH_ar_), 127.76 (CH_ar_), 127.86 (CH_ar_), 128.37 (CH_ar_), 131.83 (CH_ar_), 141.06 (C_ar_), 141.46 (C_ar_), 142.28 (C_ar_), 168.25 (C=O) ^77^Se NMR (400 MHz, DMSO) δ = 877.05 ppm; IR = 3259, 2922, 2853, 1621, 1533, 1458, 1446, 1349, 1311, 1259, 1047, 1026, 733, 680, 517, 475, 415 cm^−1^. Elemental Anal. Calcd for C_16_H_13_NO_2_Se (331.01): C, 58.19; H, 3.97; N, 4.24; Found C, 58.11; H, 3.96; N, 4.29.

*N*-[(1*R*,2*S*)-(+)-*cis*-2-hydroxy-1-indanyl]-1,2-benzisoselenazol-3(2*H*)-one **29a**

Yield: 51%; mp 187–189 °C; ${\left[\propto \right]}_{D}^{20}$ = +100 (c = 0.59, CHCl_3_);

^1^H NMR (700 MHz, DMSO) δ = 2.89–2.92 (m, 1H), 3.17–3.21 (m, 1H), 4.60–4.64 (m, 1H), 5.59 (d, J = 4.2 Hz, 1H), 5.98 (d, J = 4.9 Hz, 1H), 6.93 (d, J = 7.7 Hz, 1H_ar_), 7.16 (t, J = 4.9 Hz, 1H_ar_), 7.26 (t, J = 7.7 Hz, 1H_ar_), 7.31–7.34 (m, 1H_ar_), 7.41–7.44 (m, 1H_ar_), 7.58–7.62 (m, 1H_ar_), 7.90–7.93 (m, 1H_ar_), 7.98 (d, J = 7.7 Hz, 1H) ^13^C NMR (400 MHz, DMSO) δ = 39.56 (CH_2_), 61.37 (CH), 74.07 (CH), 124.74 (CH_ar_), 125.68 (CH_ar_), 125.71 (CH_ar_), 125.78 (CH_ar_), 126.90 (CH_ar_), 127.76 (CH_ar_), 127.86 (CH_ar_), 128.37 (CH_ar_), 131.82 (CH_ar_), 141.06 (C_ar_), 141.47 (C_ar_), 142.26 (C_ar_), 168.24 (C=O) ^77^Se NMR (400 MHz, DMSO) δ = 877.00 ppm; IR = 3234, 2950, 2853, 1602, 1566, 1457, 1445, 1337, 1312, 1271, 1061, 1019, 756, 738, 677, 517, 476, 417 cm^−1^. Elemental Anal. Calcd for C_16_H_13_NO_2_Se (331.01): C, 58.19; H, 3.97; N, 4.24; Found C, 58.13; H, 3.98; N, 4.25.

*N*-[(1*S*,2*S*)-(+)-*trans*-2-hydroxy-1-indanyl]-1,2-benzisoselenazol-3(2*H*)-one **30a**

Yield: 87%; mp 204–206 °C; ${\left[\propto \right]}_{D}^{20}$ = +212 (c = 0.55, CHCl_3_);

^1^H NMR (700 MHz, DMSO) δ = 2.80–2.84 (m, 1H), 3.24–3.28 (m, 1H), 4.43 (kw, J = 5.6 Hz, 1H), 5.57 (d, J = 4.9 Hz, 1H), 5.70 (d, J = 5.6 Hz, 1H), 7.10 (d, J = 7.7 Hz, 1H_ar_), 7.20–7.24 (m, 1H_ar_), 7.31 (d, J = 4.1 Hz, 2H_ar_), 7.44–7.48 (m, 1H_ar_), 7.60–7.63 (m, 1H_ar_), 7.87–7.90 (m, 1H_ar_), 7.98 (d, J = 7.7 Hz, 1H) ^13^C NMR (300 MHz, DMSO) δ = 39.01 (CH_2_), 65.63 (CH), 78.88 (CH), 125.18 (CH_ar_), 125.46 (CH_ar_), 126.14 (CH_ar_), 126.31 (CH_ar_), 127.32 (CH_ar_), 127.80 (CH_ar_), 128.88 (CH_ar_), 129.01 (C_ar_), 131.95 (CH_ar_), 139.56 (C_ar_), 140.85 (C_ar_), 140.95 (C_ar_), 167.38 (C=O) ^77^Se NMR (400 MHz, DMSO) δ = 824.06 ppm; IR = 3091, 2923, 2850, 1590, 1562, 1460, 1445, 1329, 1312, 1268, 1072, 1021, 793, 743, 733, 676, 516, 458 cm^−1^. Elemental Anal. Calcd for C_16_H_13_NO_2_Se (331.01): C, 58.09; H, 3.98; N, 4.26; Found C, 58.15; H, 3.97; N, 4.22.

*N*-[(1*R*,2*R*)-(−)-*trans*-2-hydroxy-1-indanyl]-1,2-benzisoselenazol-3(2*H*)-one **31a**

Yield: 66%; mp 205–207 °C; ${\left[\propto \right]}_{D}^{20}$ = −214 (c = 0.57, CHCl_3_);

^1^H NMR (700 MHz, DMSO) δ = 2.80–2.85 (m, 1H), 3.24–3.28 (m, 1H), 4.43 (kw, J = 5.6 Hz, 1H), 5.56 (d, J = 4.9 Hz, 1H), 5.69 (d, J = 5.6 Hz, 1H), 7.10 (d, J = 9.1 Hz, 1H_ar_), 7.20–7.24 (m, 1H_ar_), 7.31 (d, J = 7.7 Hz, 2H_ar_), 7.44–7.47 (m, 1H_ar_), 7.60–7.63 (m, 1H_ar_), 7.88–7.90 (m, 1H_ar_), 7.98 (d, J = 7.7 Hz, 1H) ^13^C NMR (400 MHz, DMSO) δ = 39.33 (CH_2_), 65.71 (CH), 78.93 (CH), 125.24 (CH_ar_), 125.51 (CH_ar_), 126.20 (CH_ar_), 126.35 (CH_ar_), 127.36 (CH_ar_), 127.85 (CH_ar_), 128.95 (CH_ar_), 129.04 (C_ar_), 131.98 (CH_ar_), 139.62 (C_ar_), 140.94 (C_ar_), 141.03 (C_ar_), 167.42 (C=O) ^77^Se NMR (400 MHz, DMSO) δ = 823.38 ppm; IR = 3090, 2923, 2852, 1590, 1562, 1460, 1445, 1329, 1312, 1269, 1071, 1021, 793, 744, 733, 676, 516, 457 cm^−1^. Elemental Anal. Calcd for C_16_H_13_NO_2_Se (331.01): C, 58.19; H, 3.97; N, 4.24; Found C, 58.15; H, 3.97; N, 4.22.

2,2′-diselenobis[*N*-(*S*)-(+)-*sec*-butylbezamide] **18b**

Yield: 66%; mp 233–235 °C; ${\left[\propto \right]}_{D}^{20}$ = +43 (c = 0.24, CHCl_3_);

^1^H NMR (700 MHz, DMSO) δ = 0.92 (t, J = 7.7 Hz, 3H), 1.18 (d, J = 7.0 Hz, 3H), 1.50–1.60 (m, 2H), 3.92–3.97 (m, 1H), 7.31–7.34 (m, 1H_ar_), 7.36–7.39 (m, 1H_ar_), 7.69 (d, J = 7.7 Hz, 1H_ar_), 7.79 (d, J = 7.7 Hz, 1H_ar_), 8.44 (d, J = 7.7 Hz, 1H_ar_) ^13^C NMR (700 MHz, DMSO) δ = 11.15 (CH_3_), 20.59 (CH_3_), 29.26 (CH_2_), 47.33 (CH), 126.55 (CH_ar_), 128.30 (CH_ar_), 130.41 (CH_ar_), 131.79 (CH_ar_), 132.31 (C_ar_), 134.35 (C_ar_), 167.36 (C=O) ^77^Se NMR (700 MHz, DMSO) δ = 443.25′ ppm; IR: 3312, 2968,, 2927, 2870, 1738, 1612, 1583, 1535, 1448, 1435, 1366, 1353, 1302, 1285, 1229, 1216, 1163, 1146, 1027, 871, 743, 677, 645, 541, 472, 446 cm^−1^. Elemental Anal. Calcd for C_22_H_28_N_2_O_2_Se_2_ (512.08): C, 51.77; H, 5.53; N, 5.49; Found C, 51.72; H, 5.53; N, 5.52.

2,2′-diselenobis[*N*-(*R*)-(−)-*sec*-butylbezamide] **19b**

Yield: 74%; mp 234–235 °C; ${\left[\propto \right]}_{D}^{20}$ = −41 (c = 0.30, CHCl_3_);

^1^H NMR (700 MHz, DMSO) δ = 0.91 (t, J = 7.7 Hz, 3H), 1.18 (d, J = 7.0 Hz, 3H), 1.50–1.60 (m, 2H), 3.92–3.97 (m, 1H), 7.31–7.34 (m, 1H_ar_), 7.36–7.39 (m, 1H_ar_), 7.69 (d, J = 8.4 Hz, 1H_ar_), 7.79 (d, J = 7.7 Hz, 1H_ar_), 8.44 (d, J = 7.7 Hz, 1H_ar_) ^13^C NMR (700 MHz, DMSO) δ = 11.15 (CH_3_), 20.60 (CH_3_), 29.27 (CH_2_), 47.32 (CH), 126.54 (CH_ar_), 128.30 (CH_ar_), 130.41 (CH_ar_), 131.78 (CH_ar_), 132.32 (C_ar_), 134.36 (C_ar_), 167.35 (C=O) ^77^Se NMR (700 MHz, DMSO) δ = 443.17 ppm; IR: 3311, 2968, 2927, 2870, 1738, 1613, 1583, 1535, 1448, 1435, 1366, 1353, 1302, 1284, 1229, 1216, 1164, 1145, 1027, 872, 743, 678, 645, 528, 472, 446 cm^−1^. Elemental Anal. Calcd for C_22_H_28_N_2_O_2_Se_2_ (512.08): C, 51.77; H, 5.53; N, 5.49; Found C, 51.70; H, 5.53; N, 5.45.

2,2′-diselenobis[*N*-(*S*)-(+)-1-hydroxy-2-butanylbezamide] **20b**

Yield: 52%; mp 200–202 °C; ${\left[\propto \right]}_{D}^{20}$ = +65 (c = 0.31, CHCl_3_);

^1^H NMR (700 MHz, DMSO) δ = 0.92 (t, J = 7.7 Hz, 3H), 1.44–1.52 (m, 1H), 1.66–1.73 (m, 1H), 3.41–3.46 (m, 1H), 3.49–3.53 (m, 1H), 3.86–3.93 (m, 1H), 4.73 (t, J = 6.3 Hz, 1H), 7.31–7.35 (m, 1H_ar_), 7.36–7.40 (m, 1H_ar_), 7.70 (dd, J_1_ = 1.4 Hz, J_2_ = 8.4 Hz, 1H_ar_), 7.84 (dd, J_1_ = 1.4 Hz, J_2_ = 8.4 Hz, 1H_ar_), 8.31 (d, J = 8.4 Hz, 1H_ar_) ^13^C NMR (700 MHz, DMSO) δ = 11.05 (CH_3_), 24.12 (CH_2_), 53.94 (CH), 63.51 (CH_2_), 126.50 (CH_ar_), 128.43 (CH_ar_), 130.40 (CH_ar_), 131.82 (CH_ar_), 132.38 (C_ar_), 134.27 (C_ar_), 167.91 (C=O) ^77^Se NMR (700 MHz, DMSO) δ = 444.00 ppm; IR: 3278, 3045, 2969, 2870, 1738, 1622, 1583, 1530, 1453, 1366, 1306, 1283, 1216, 1179, 1165, 1076, 1060, 1048, 1025, 873, 727, 692, 644, 557, 447 cm^−1^. Elemental Anal. Calcd for C_22_H_28_N_2_O_4_Se_2_ (544.04): C, 48.72; H, 5.20; N, 5.16; Found C, 48.65; H, 5.21; N, 5.11.

2,2′-diselenobis[*N*-(*R*)-(−)-1-hydroxy-2-butanylbezamide] **21b**

Yield: 46%; mp 199–201 °C; ${\left[\propto \right]}_{D}^{20}$ = −67 (c = 0.41, CHCl_3_);

^1^H NMR (700 MHz, DMSO) δ = 0.89 (t, J = 7.0 Hz, 3H), 1.41–1.49 (m, 1H), 1.63–1.70 (m, 1H), 3.38–3.43 (m, 1H), 3.46–3.50 (m, 1H), 3.83–3.89 (m, 1H), 4.69 (t, J = 5.6 Hz, 1H), 7.27–7.31 (m, 1H_ar_), 7.33–7.37 (m, 1H_ar_), 7.67 (dd, J_1_ = 0.7 Hz, J_2_ = 8.4 Hz, 1H_ar_), 7.81 (dd, J_1_ = 1.4 Hz, J_2_ = 7.7 Hz, 1H_ar_), 8.27 (d, J = 8.4 Hz, 1H_ar_) ^13^C NMR (700 MHz, DMSO) δ = 11.05 (CH_3_), 24.12 (CH_2_), 53.95 (CH), 63.51 (CH_2_), 126.50 (CH_ar_), 128.43 (CH_ar_), 130.42 (CH_ar_), 131.82 (CH_ar_), 132.36 (C_ar_), 134.27 (C_ar_), 167.92 (C=O) ^77^Se NMR (700 MHz, DMSO) δ= 443.96 ppm; IR: 3276, 3047, 2969, 2870, 1738, 1622, 1584, 1530, 1453, 1365, 1306, 1283, 1216, 1179, 1165, 1076, 1060, 1047, 1025, 873, 727, 691, 644, 557, 448 cm^−1^. Elemental Anal. Calcd for C_22_H_28_N_2_O_4_Se_2_ (544.04): C, 48.72; H, 5.20; N, 5.16; Found C, 48.69; H, 5.20; N, 5.09.

2,2′-diselenobis[*N*-(*R*)-(−)-1,2,3,4-tetrahydro-1-napthylbezamide] **22b**

Yield: 58%; mp 266–268 °C; ${\left[\propto \right]}_{D}^{20}$ = −167 (c = 0.21, CHCl_3_);

^1^H NMR (700 MHz, DMSO) δ = 1.69–1.81 (m, 1H), 1.81–1.88 (m, 1H), 1.94–2.05 (m, 2H), 2.72–2.82 (m, 2H), 5.25 (q, J = 7Hz, 1H), 7.09–7.19 (m, 3H_ar_), 7.23–7.31 (m, 2H_ar_), 7.34–7.39 (m, 1H_ar_), 7.74 (dd, J_1_ = 1.4 Hz, J_2_ = 7.7 Hz, 1H_ar_), 7.81 (dd, J_1_ = 1.4 Hz, J_2_ = 7.7 Hz, 1H_ar_), 9.05 (d, J = 9.1 Hz, 1H) ^13^C NMR (700 MHz, DMSO) δ = 20.84 (CH_2_), 29.37 (CH_2_), 30.30 (CH_2_), 48.02 (CH), 126.35 (CH_ar_), 126.60 (CH_ar_), 127.24 (CH_ar_), 128.35 (CH_ar_), 128.56 (CH_ar_), 129.27 (CH_ar_), 130.52 (CH_ar_), 131.99 (C_ar_), 132.62 (CH_ar_), 134.02 (C_ar_), 137.73 (C_ar_), 137.76 (C_ar_), 167.61 (C=O) ^77^Se NMR (700 MHz, DMSO) δ= 445.03 ppm; IR: 3279, 2940, 2923, 1627, 1583, 1530, 1486, 1430, 1335, 1256, 1080, 1024, 878, 865, 758, 682, 593, 447 cm^−1^. Elemental Anal. Calcd for C_34_H_32_N_2_O_2_Se_2_ (660.08): C, 62.01; H, 4.90; N, 4.25; Found C, 61.95; H, 4.91; N, 4.17.

2,2′-diselenobis[*N*-(*S*)-(+)-1,2,3,4-tetrahydro-1-napthylbezamide] **23b**

Yield: 86%; mp 266–268 °C; ${\left[\propto \right]}_{D}^{20}$ = +166 (c = 0.25, CHCl_3_);

^1^H NMR (700 MHz, DMSO) δ = 1.72–1.84 (m, 1H), 1.85–1.91 (m, 1H), 1.97–2.08 (m, 2H), 2.74–2.85 (m, 2H), 5.28 (q, J = 8.4 Hz, 1H), 7.12–7.21 (m, 3H_ar_), 7.26–7.34 (m, 2H_ar_), 7.38–7.42 (m, 1H_ar_), 7.77 (dd, J_1_ = 1.4 Hz, J_2_ = 7.7 Hz, 1H_ar_), 7.85 (dd, J_1_ = 1.4 Hz, J_2_ = 7.7 Hz, 1H_ar_), 9.08 (d, J = 9.1 Hz, 1H) ^13^C NMR (700 MHz, DMSO) δ = 20.84 (CH_2_), 29.37 (CH_2_), 30.30 (CH_2_), 48.02 (CH), 126.36 (CH_ar_), 126.60 (CH_ar_), 127.24 (CH_ar_), 128.35 (CH_ar_), 128.56 (CH_ar_), 129.28 (CH_ar_), 130.52 (CH_ar_), 131.99 (C_ar_), 132.62 (CH_ar_), 134.01 (C_ar_), 137.72 (C_ar_), 137.76 (C_ar_), 167.62 (C=O) ^77^Se NMR (700 MHz, DMSO) δ = 445.22 ppm; IR: 3275, 2939, 2922, 1627, 1583, 1530, 1486, 1430, 1334, 1255, 1079, 1024, 878, 864, 758, 681, 593, 427 cm^−1^. Elemental Anal. Calcd for C_34_H_32_N_2_O_2_Se_2_ (660.08): C, 62.01; H, 4.90; N, 4.25; Found C, 62.00; H, 4.89; N, 4.21.

2,2′-diselenobis[*N*-(*R*)-(+)-α-methylbenzylbezamide] **24b**

Yield: 92%; mp 200–202 °C; ${\left[\propto \right]}_{D}^{20}$ = +101 (c = 0.47, CHCl_3_) (lit. mp 219–220°C; ${\left[\propto \right]}_{D}^{20}$ = +172 (c = 1.00, dioxane) [13])

^1^H NMR (700 MHz, DMSO) δ = 1.52 (d, J = 7 Hz, 3H), 5.17–5.22 (m, 1H), 7.23–7.27 (m, 1H_ar_), 7.33–7.39 (m, 4H_ar_), 7.43–7.45 (m, 2H_ar_), 7.67 (dd, J_1_ = 1.4 Hz, J_2_ = 7.7 Hz, 1H_ar_), 7.92 (dd, J_1_ = 1.4 Hz, J_2_ = 7.7 Hz, 1H_ar_), 9.09 (d, J = 7.7 Hz, 1H) ^13^C NMR (700 MHz, DMSO) δ = 22.61 (CH_3_), 49.30 (CH), 126.57 (2xCH_ar_), 126.58 (CH_ar_), 127.19 (CH_ar_), 128.60 (CH_ar_), 128.75 (2xCH_ar_), 130.46 (CH_ar_), 132.03 (CH_ar_), 132.53 (C_ar_), 133.78 (C_ar_), 144.95 (C_ar_), 167.15 (C=O) ^77^Se NMR (700 MHz, DMSO) δ = 444.99 ppm; IR: 3312, 3061, 3033, 2976, 2930, 1625, 1583, 1524, 1451, 1431, 1376, 1325, 1207, 1133, 1091, 873, 709, 696, 674, 644, 472, 443 cm^-1^. Elemental Anal. Calcd for C_30_H_28_N_2_O_2_Se_2_ (608.05): C, 59.41; H, 4.65; N, 4.62; Found C, 59.31; H, 4.64; N, 4.55.

2,2′-diselenobis[*N*-(*S*)-(−)-α-methylbenzylbezamide] **25b**

Yield: 81%; mp 200–202 °C; ${\left[\propto \right]}_{D}^{20}$ = −104 (c = 0.63, CHCl_3_) (lit. mp 219–220 °C; ${\left[\propto \right]}_{D}^{20}$ = −172 (c = 1.00, dioxane) [13])

^1^H NMR (700 MHz, DMSO) δ = 1.52 (d, J = 7 Hz, 3H), 5.17–5.23 (m, 1H), 7.23–7.27 (m, 1H_ar_) 7.32–7.40 (m, 4H_ar_), 7.43–7.46 (m, 2H_ar_), 7.66–7.70 (m, 1H_ar_), 7.92 (dd, J_1_ = 0.7 Hz, J_2_ = 7.7 Hz, 1H_ar_), 9.09 (d, J = 8.4 Hz, 1H) ^13^C NMR (700 MHz, DMSO) δ = 22.62 (CH_3_), 49.30 (CH), 126.57 (2xCH_ar_), 126.58 (CH_ar_), 127.19 (CH_ar_), 128.61 (CH_ar_), 128.75 (2xCH_ar_), 130.45 (CH_ar_), 132.03 (CH_ar_), 132.52 (C_ar_), 133.76 (C_ar_), 144.96 (C_ar_), 167.15 (C=O) ^77^Se NMR (700 MHz, DMSO) δ= 445.01 ppm; IR: 3310, 3059, 3032, 2975, 1623, 1583, 1522, 1448, 1431, 1376, 1324, 1207, 1132, 1090, 872, 696, 671, 644, 471, 443 cm^−1^. Elemental Anal. Calcd for C_30_H_28_N_2_O_2_Se_2_ (608.05): C, 59.41; H, 4.65; N, 4.62; Found C, 59.32; H, 4.65; N, 4.59.

2,2′-diselenobis[*N*-(*S*)-(−)-1-(1-napthyl)ethylbezamide] **26b**

Yield: 70%; mp 262–64 °C; ${\left[\propto \right]}_{D}^{20}$ = −96 (c = 0.35, CHCl_3_);

^1^H NMR (700 MHz, DMSO) δ = 1.66 (d, J = 7.7 Hz, 3H), 5.95–6.01 (m, 1H), 7.31–7.36 (m, 2H_ar_), 7.49–7.56 (m, 2H_ar_), 7.58–7.62 (m, 1H_ar_), 7.65–7.69 (m, 2H_ar_), 7.86 (d, J = 8.4 Hz, 1H_ar_), 7.90–7.94 (m, 1H_ar_), 7.95–7.99 (m, 1H_ar_), 8.26 (d, J = 8.4 Hz, 1H_ar_), 9.26 (d, J = 7.7 Hz, 1H) ^13^C NMR (700 MHz, DMSO) δ = 21.87 (CH_3_), 45.64 (CH), 123.11 (CH_ar_), 123.63 (CH_ar_), 125.95 (CH_ar_), 126.09 (CH_ar_), 126.60 (CH_ar_), 126.73 (CH_ar_), 127.85 (CH_ar_), 128.67 (CH_ar_), 129.17 (CH_ar_), 130.40 (CH_ar_), 130.90 (CH_ar_), 132.03 (C_ar_), 132.45 (C_ar_), 133.76 (C_ar_), 133.90 (C_ar_), 140.44 (C_ar_), 167.10 (C=O) ^77^Se NMR (700 MHz, DMSO) δ = 442.72 ppm; IR: 3290, 2922, 1625, 1583, 1530, 1452, 1429, 1338, 1308, 1284, 1258, 1180, 1132, 1025, 871, 792, 773, 735, 693, 641, 448 cm^−1^.Elemental Anal. Calcd for C_38_H_32_N_2_O_2_Se_2_ (708.08): C, 64.59; H, 4.56; N, 3.96; Found C, 64.46; H, 4.55; N, 3.89.

2,2′-diselenobis[*N*-(*R*)-(+)-1-(1-napthyl)ethylbezamide] **27b**

Yield: 73%; mp 262–264 °C; ${\left[\propto \right]}_{D}^{20}$ = +98 (c = 0.32, CHCl_3_);

^1^H NMR (700 MHz, DMSO) δ = 1.66 (d, J = 7.7 Hz, 3H), 5.55–6.01 (m, 1H), 7.31–7.36 (m, 2H_ar_), 7.49–7.56 (m, 2H_ar_), 7.58–7.61 (m, 1H_ar_), 7.66–7.69 (m, 2H_ar_), 7.86 (d, J = 8.4 Hz, 1H_ar_), 7.90–7.93 (m, 1H_ar_), 7.96–7.98 (m, 1H_ar_), 8.26 (d, J = 8.4 Hz, 1H_ar_), 9.26 (d, J = 7.7 Hz, 1H) ^13^C NMR (700 MHz, DMSO) δ = 21.87 (CH_3_), 45.64 (CH), 123.12 (CH_ar_), 123.64 (CH_ar_), 125.96 (CH_ar_), 126.09 (CH_ar_), 126.59 (CH_ar_), 126.71 (CH_ar_), 127.85 (CH_ar_), 128.67 (CH_ar_), 129.17 (CH_ar_), 130.41 (CH_ar_), 130.91 (CH_ar_), 132.03 (C_ar_), 132.64 (C_ar_), 133.79 (C_ar_), 133.91 (C_ar_), 140.45 (C_ar_), 167.10 (C=O) ^77^Se NMR (700 MHz, DMSO) δ = 443.08 ppm; IR: 3292, 3063, 2976, 2927, 1624, 1583, 1531, 1450, 1432, 1337, 1306, 1283, 1258, 1180, 1131, 1083, 1026, 997, 878, 793, 773, 736, 725, 692, 641, 447 cm^−1^. Elemental Anal. Calcd for C_38_H_32_N_2_O_2_Se_2_ (708.08): C, 64.59; H, 4.56; N, 3.96; Found C, 64.52; H, 4.57; N, 3.90.

2,2′-diselenobis[*N*-(1*S*,2*R*)-(−)-*cis*-2-hydroxy-1-indanylbezamide] **28b**

Yield: 70%; mp 241–243 °C; ${\left[\propto \right]}_{D}^{20}$ = −149 (c = 0.39, CHCl_3_);

^1^H NMR (700 MHz, DMSO) δ = 2.90–2.95 (m, 1H), 3.14 (dd, J_1_ = 4.9 Hz, J_2_ = 9.1 Hz, 1H), 4.56–4.61 (m, 1H), 5.17–5.21 (m, 1H), 5.49 (dd, J_1_ = 5.6 Hz, J_2_ = 8.4 Hz, 1H), 7.21–7.27 (m, 2H_ar_), 7.28–7.35 (m, 3H_ar_), 7.40–7.44 (m, 1H_ar_), 7.78 (d, J = 8.4 Hz, 1H_ar_), 8.01 (d, J = 7.7 Hz, 1H_ar_), 8.59 (d, J = 8.4 Hz, 1H_ar_) ^13^C NMR (700 MHz, DMSO) δ= 39.92 (CH_2_), 58.10 (CH), 72.54 (CH), 124.94 (CH_ar_), 125.35 (CH_ar_), 126.63 (CH_ar_), 126.86 (CH_ar_), 128.00 (CH_ar_), 129.00 (CH_ar_), 130.42 (CH_ar_), 132.16 (CH_ar_), 132.66 (C_ar_), 133.62 (C_ar_), 141.44 (C_ar_), 142.02 (C_ar_), 168.21 (C=O) ^77^Se NMR (700 MHz, DMSO) δ = 445.85 ppm; IR: 3288, 3046, 2976, 2926, 1622, 1582, 1529, 1428, 1337, 1305, 1281, 1216, 1080, 1047, 997, 873, 792, 772, 735, 691, 558, 447 cm^−1^. Elemental Anal. Calcd for C_32_H_28_N_2_O_4_Se_2_ (664.04): C, 58.01; H, 4.26; N, 4.23; Found C, 58.05; H, 4.27; N, 4.17.

2,2′-diselenobis[*N*-(1*R*,2*S*)-(+)-*cis*-2-hydroxy-1-indanylbezamide] **29b**

Yield: 73%; mp 241–243 °C; ${\left[\propto \right]}_{D}^{20}$ = +152 (c = 0.41, CHCl_3_);

^1^H NMR (700 MHz, DMSO) δ = 2.91–2,95 (m, 1H), 3.14 (dd, J_1_ = 4.9 Hz, J_2_ = 16.1 Hz, 1H), 4.46 (qd, J_1_ = 2.1 Hz, J_2_ = 4.9 Hz, 1H), 5.18 (d, J = 4.9 Hz, 1H), 5.49 (dd, J_1_ = 5.6 Hz, J_2_ = 8.4 Hz, 1H), 7.21–7.27 (m, 2H_ar_), 7.28–7.35 (m, 3H_ar_), 7.40–7.44 (m, 1H_ar_), 7.78 (d, J = 8.4 Hz, 1H_ar_), 8.01 (d, J = 7.7 Hz, 1H_ar_), 8.59 (d, J = 8.4 Hz, 1H_ar_) ^13^C NMR (700 MHz, DMSO) δ = 39.70 (CH_2_), 58.10 (CH), 72.53 (CH), 124.94 (CH_ar_), 125.34 (CH_ar_), 126.63 (CH_ar_), 126.86 (CH_ar_), 128.00 (CH_ar_), 129.00 (CH_ar_), 130.42 (CH_ar_), 132.15 (CH_ar_), 132.65 (C_ar_), 133.62 (C_ar_), 141.43 (C_ar_), 142.02 (C_ar_), 168.21 (C=O) ^77^Se NMR (700 MHz, DMSO) δ = 445.85 ppm; IR: 3289, 3047, 2974, 2927, 1622, 1583, 1530, 1431, 1337, 1306, 1282, 1210, 1082, 1046, 997, 877, 793, 772, 735, 692, 558, 447 cm^−1^. Elemental Anal. Calcd for C_32_H_28_N_2_O_4_Se_2_ (664.04): C, 58.01; H, 4.26; N, 4.23; Found C, 58.09; H, 4.27; N, 4.17.

2,2′-diselenobis[*N*-(1*S*,2*S*)-(−)-*trans*-2-hydroxy-1-indanylbezamide] **30b**

Yield: 93%; mp 234–236 °C; ${\left[\propto \right]}_{D}^{20}$ = +153 (c = 0.33, CHCl_3_);

^1^H NMR (700 MHz, DMSO) δ = 2.76 (dd, J_1_ = 7.7 Hz, J_2_ = 15.4 Hz, 1H), 3.18 (dd, J_1_ = 7.7 Hz, J_2_ = 15.4 Hz, 1H), 4.46 (kwintet, J = 7 Hz, 1H), 5.29 (t, J = 7.7 Hz, 1H), 5.41 (d, J = 5.6 Hz, 1H), 7.17–7.23 (m, 4H_ar_), 7.30–7.34 (m, 1H_ar_), 7.37–7.41 (m, 1H_ar_), 7.76 (dd, J_1_ = 0.7 Hz, J_2_ = 7.7 Hz, 1H_ar_), 7.88 (dd, J_1_ = 0.7 Hz, J_2_ = 7.7 Hz, 1H_ar_), 9.03 (d, J = 8.4 Hz, 1H) ^13^C NMR (700 MHz, DMSO) δ= 39.34 (CH_2_), 62.39 (CH), 77.89 (CH), 124.41 (CH_ar_), 125.16 (CH_ar_), 126.59 (CH_ar_), 127.19 (CH_ar_), 128.17 (CH_ar_), 128.64 (CH_ar_), 130.54 (CH_ar_), 132.09 (CH_ar_), 132.67 (C_ar_), 133.88 (C_ar_), 140.46 (C_ar_), 142.18 (C_ar_), 168.32 (C=O) ^77^Se NMR (700 MHz, DMSO) δ = 445.85 ppm; IR: 3254, 2919, 1630, 1584, 1530, 1456, 1456, 1428, 1345, 1272, 1214, 1122, 1065, 919, 871, 685, 646, 579, 517, 447 cm^−1^. Elemental Anal. Calcd for C_32_H_28_N_2_O_4_Se_2_ (664.04): C, 58.01; H, 4.26; N, 4.23; Found C, 58.05; H, 4.27; N, 4.19.

2,2′-diselenobis[*N*-(1*R*,2*R*)-(−)-*trans*-2-hydroxy-1-indanylbezamide] **31b**

Yield: 86%; mp 233–235 °C; ${\left[\propto \right]}_{D}^{20}$ = −150 (c = 0.33, CHCl_3_);

^1^H NMR (700 MHz, DMSO) δ = 2.76 (dd, J_1_ = 7.7 Hz, J_2_ = 15.4 Hz, 1H), 3.18 (dd, J_1_ = 7.7 Hz, J_2_ = 15.4 Hz, 1H), 4.46 (kwintet, J = 7 Hz, 1H), 5.30 (t, J = 7.7 Hz, 1H), 5.40 (d, J = 5.6 Hz, 1H), 7.15–7.24 (m, 4H_ar_), 7.30–7.34 (m, 1H_ar_), 7.36–7.42 (m, 1H_ar_), 7.76 (dd, J_1_ = 0.7 Hz, J_2_ = 7.7 Hz, 1H_ar_), 7.88 (dd, J_1_ = 0.7 Hz, J_2_ = 7.7 Hz, 1H_ar_), 9.01 (d, J = 8.4 Hz, 1H) ^13^C NMR (700 MHz, DMSO) δ = 39.31 (CH_2_), 62.36 (CH), 77.88 (CH), 124.41 (CH_ar_), 125.16 (CH_ar_), 126.59 (CH_ar_), 127.20 (CH_ar_), 128.17 (CH_ar_), 128.64 (CH_ar_), 130.52 (CH_ar_), 132.09 (CH_ar_), 132.67 (C_ar_), 133.84 (C_ar_), 140.44 (C_ar_), 142.17 (C_ar_), 168.32 (C=O) ^77^Se NMR (700 MHz, DMSO) δ = 445.76 ppm; IR: 3257, 2920, 1630, 1584, 1531, 1458, 1429, 1345, 1272, 1213, 1123, 1066, 920, 871, 658, 649, 580, 518, 448 cm^−1^. Elemental Anal. Calcd for C_32_H_28_N_2_O_4_Se_2_ (664.04): C, 58.01; H, 4.26; N, 4.23; Found C, 57.92; H, 4.27; N, 4.18.

### 2.3. Antioxidant Activity Assay

The antioxidant activity assay was prepared via the method presented by Iwaoka [22].

### 2.4. MTT Viability Assay

The MTT assay was based on the method of Mosmann [23]. 

### 2.5. Crystal Structure Determination

The crystal structures of **24a** and **25a** were determined. The X-ray diffraction data were collected at a temperature of 100(1) K with a Rigaku XtaLAB Synergy CCD diffractometer using CuKα radiation λ=1.54184 Å. Both structures were solved by direct methods and refined with the full-matrix least-squares method on F^2^ with the use of the SHELX2017 [24,25] program package. The absorption corrections were used with CrysAlisPro 1.171.41.120a (Rigaku OD, 2021), [26]. The hydrogen atoms were located from the electron density maps, and their positions were constrained in the refinement. Details of the diffraction experiments and crystal structures are presented in the Supplemental Materials. The crystallographic data were deposited with the Cambridge Crystallographic Data Centre, the CCDC numbers: 2014858 and 2014859 for **24a** and **25a**, respectively.

## 3. Results and Discussion

**Scheme 2:**
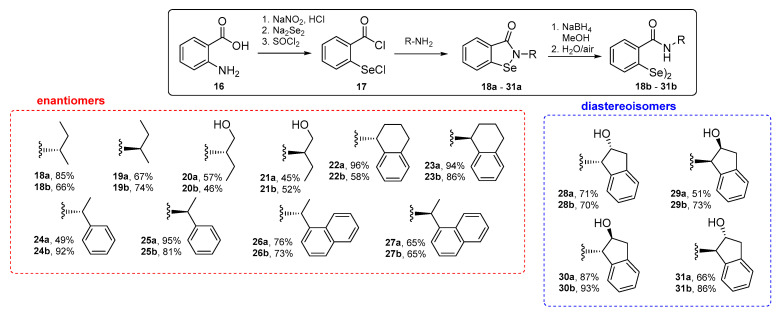
Synthetized chiral benzisoselenazolones **18a**–**31a** and diselenides **18b**–**31b**.

The first step of the research involved the synthesis of *N*-substituted benzisoselenazol-3(2H)-ones. The procedure was based on the reaction of 2-(chloroseleno)-benzoyl chloride **17**, obtained according to our previously reported method [18], with commercially available chiral amines. Then, the obtained compounds **18a**–**31a** were transformed into diselenides **18b**–**31b** by a sodium borohydride reduction and air oxidation protocol. Both benzisoselenazolones (45–96%) and diselenides (46–93%) were synthesized in good yields (Scheme 2).

**Figure 3:**
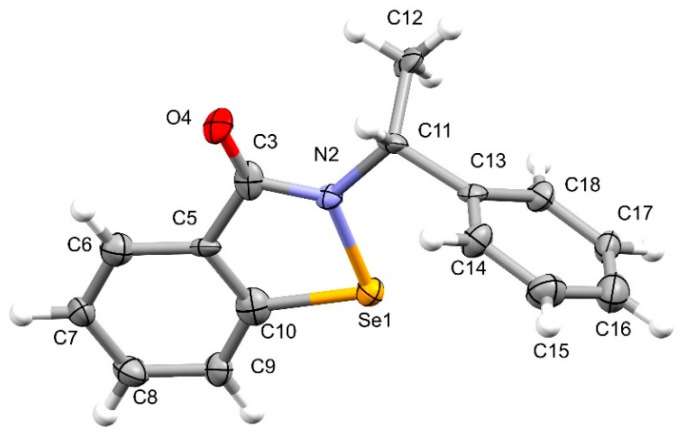
Molecule 1 of **24a** with atomic displacement parameters plotted at a 50% probability level. The atom-numbering scheme is analogous for all molecules in both enantiomers **24a** and **25a**.

Furthermore, the crystal structures of enantiomers **24a**/**24b** were determined. The asymmetric part of the crystal structure of *N*-[(*R*)-(+)-*α*-methylbenzyl]-1,2-benzisoselenazol-3(2*H*)-one **24a** consists of five molecules (Supplemental Materials). The single molecule with the atom numbering scheme is shown in Figure 3. In all of them, the (*R*) configuration of the chiral center was detected. The molecular conformation differs slightly between the molecules, with the dihedral angles between the phenyl ring and the benzisoselenazolone moiety being 84.8(8)°, 82.3(9)°, 85.1(9)°, 76.3(9)° and 83.2(9)° for molecules 1–5, respectively. Significant differences in the conformation of molecules 1–5 were detected, affecting the positions of the methyl groups relative to the benzisoselenazolone moieties. The representative Se1–N2–C11–C12 torsion angle of its equivalents in molecules 2–5 are 102.2(15)°, 82.7(19)°, 93.9(19)°, 85(2)° and 64(2)°. The network of short intermolecular Se…O contacts involving four molecules is found in the structure. The respective distances are Se1…O4[3/2 − x, 1 − y, 1/2 + z] 2.592(13), Se21…O44[x, y, 1 + z] 2.640(13), Se41…O24 2.627(18), Se61…O84[x, y, −1 + z] 2.761(14) and Se81…O64 2.677(16) Å. 

In the crystal structure of *N*-[(*S*)-(+)-*α*-methylbenzyl]-1,2-benzisoselenazol-3(2*H*)-one **25a**, the asymmetric part also consists of five benzisoselenazolone molecules. For all molecules, the S configuration of the benzyl C11 chiral centers was found. The dihedral angles between the phenyl and benzisoselenazolone moieties in molecules 1–5 are 84.6(3)°, 85.8(4)°, 82.5(4)°, 82.7(4)° and 76.4(4)°. Due to the opposite enantiomer, the Se1–N2–C11–C12 torsion angles have negative signs: −102.5(6)°, −94.5(8)°, −81.8(9)°, −65.6(9)° and −84.4(10)° for molecules 1–5, respectively. In **25a,** the similar network of Se…O intermolecular interactions is found, with the respective distances Se1…O4[1 − x, −1/2 + y, 1/2−z] 2.592(5), Se21...O44[x, −1 + y, z] 2.633(6), Se41…O24 2.621(5), Se61…O84 2.685(6) and Se81…O64[x, 1 + y, z] 2.779(6) Å. 

The antioxidant activity of all the obtained compounds was evaluated using the broadly used test presented by Iwaoka [22]. The Se-catalyst reduces hydrogen peroxide and regenerates in the presence of dithiol (DTT^red^). The rate of the reaction is measured using ^1^H NMR spectroscopy. The appearance of signals representing the formed disulfide (DTT^ox^) in specific time intervals is recorded (Table 1).

The antioxidant activity of all enantiomeric pairs was the same. In general, better results were obtained for diselenides **18b**–**31b** than for the corresponding benzisoselenazolones **18a**–**31a**. The best H_2_O_2_-scavenging properties were observed for the indanyl derivatives possessing a *cis* (**28b**/**29b**) and *trans* (**30b**/**31b**) 2-hydroxy group, with the total dithiol conversion after 30 and 15 min, respectively. 

The cytotoxic activity of Se-compounds was measured using a cell viability assay (MTT) on human promyelocytic leukemia HL-60 and breast cancer MCF-7 cell lines [23]. In the case of benzisoselenazolones, the highest antiproliferative potential was also observed for the hydroxyindanyl derivative **28a** (Table 2).

**Figure 4:**
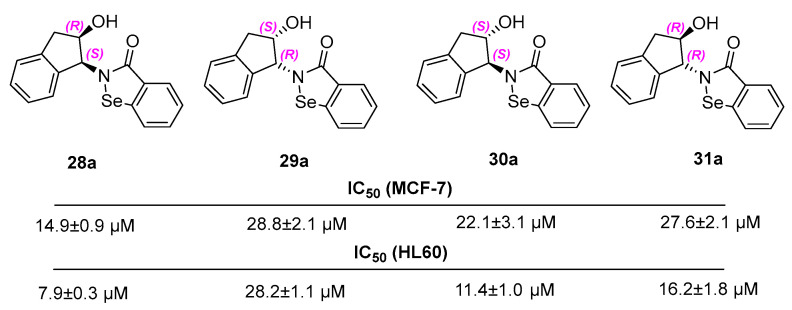
Different activity of diastereomeric hydroxyindanyl derivatives **28a**–**31a**.

The bio-activity of the *N*-(2-hydroksy-1-indanyl)-1,2-benzizoselenazol-3(2*H*)-ones **28a**–**31a** depended on the stereochemistry of the C1 carbon of the *N*-substituent. The IC_50_ values were lower for the (*S*)-configuration of C1, directly connected to the nitrogen atom. The stereochemistry of C2, with the attached hydroxy group, seemed to not influence the reactivity (Figure 4).

The corresponding diselenides only expressed cytotoxicity towards the HL-60 cell line, except for derivative **20b** (IC_50_ 37.00 ± 4.25 µM). This suggests that the Se–N bond is needed to influence the proliferation of MCF-7 cells. Additionally, the cytotoxic activity was exclusively observed for diselenides bearing an additional hydroxy group. The hydroxybuthyl derivatives **20b** and **21b** gave the lowest IC_50_ values 8.67 ± 0.14 µM and 10.10 ± 0.49 µM, respectively. Most potent compounds were also evaluated using human endothelial cells HUVEC. The *N*-*trans*-2-hydroksy-1-indanyl diselenide **31b** revealed a selective antiproliferative activity with no toxicity towards normal cells (Table 3).

## 4. Conclusions

Herein, we reported the synthesis of chiral *N*-substituted benzisoselenazol-3(2H)-ones and the corresponding diselenides with *o*-amido function, creating a series of enantiomers and diastereoisomers. The 28 obtained derivatives possess various moieties on the nitrogen atom, including aliphatic acyclic and cyclic carbon chains with additional aromatic rings and hydroxy groups. All compounds were tested as antioxidants and anticancer agents. The obtained results revealed that: diselenides are generally better antioxidants with a significant activity enhancement by the presence of *N*-hydroxyindanyl moiety; the cytotoxic activity of benzisoselenazolones is similar towards both HL-60 and MCF-7 cell lines with the highest antiproliferative potential for *N*-(2-hydroksy-1-indanyl) derivatives having a (*S*)-configuration of the C1_indanyl_ carbon directly connected to the nitrogen atom of the selenazolone ring; the anticancer activity of diselenides is only selectively expressed towards HL-60 cell lines, revealing that the potential to influence the proliferation of breast cancer cells MCF-7 is connected to the presence of the benzisoselenazolone core; the attachment of a hydroxy group seems to be essential to obtain a cytotoxic effect on human promyelocytic leukemia cell lines; the IC_50_ values obtained for selected diselenides using human endothelial cells HUVEC showed that these compounds can express a selective cytotoxic effect with a minimal disruption of the normal cells. It can be concluded that the hydroxyindanyl moiety attached to the nitrogen atom of the benzisoselenazolone or the *o*-amidodiselenide core enables the improvement of the bio-activity of the Se-compounds and can be considered a useful motif in further modification.

## Figures and Tables

**Table 1:** Results of the antioxidant activity measurement.

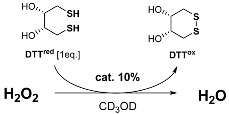				
		Remaining DTT^red^ (%)		
Catalyst (0.1 equiv.)	5 min	15 min	30 min	60 min
Benzisoselenazolones				
**18a/19a**	90	88	86	80
**20a/21a**	86	83	77	60
**22a/23a**	91	88	85	77
**24a/25a**	77	59	38	16
**26a/27a**	90	89	88	84
**28a/29a**	84	80	74	62
**30a/31a**	87	86	83	78
Diselenides				
**18b/19b**	80	65	47	29
**20b/21b**	73	50	30	19
**22b/23b**	89	83	80	75
**24b/25b**	76	54	37	26
**26b/27b**	87	84	82	76
**28b/29b**	44	13	2	0
**30b/31b**	14	2	0	0
Ebselen	75	64	58	52

**Table 2:** The antiproliferative activity of compounds **18a**–**31a**.

Compound	IC_50_ (µM) ± SEM	
	HL-60	MCF-7
**18a**	48.5 ± 6.3	36.5 ± 4.4
**19a**	71.2 ± 0.1	19.2 ± 1.3
**20a**	26.0 ± 1.7	38.3 ± 1.3
**21a**	33.3 ± 0.5	35.1 ± 0.5
**22a**	18.5 ± 0.5	62.3 ± 3.8
**23a**	26.5 ± 3.5	46.0 ± 0.8
**24a**	16.1 ± 0.0	32.8 ± 2.8
**25a**	16.8 ± 0.4	38.8 ± 0.8
**26a**	47.5 ± 6.2	27.2 ± 0.1
**27a**	52.6 ± 0.2	41.1 ± 1.3
**28a**	7.9 ± 0.3	14.9 ± 0.9
**29a**	28.2 ± 1.1	28.8 ± 2.1
**30a**	11.4 ± 1.0	22.1 ± 3.1
**31a**	16.2 ± 1.8	27.6 ± 2.1

**Table 3:** The antiproliferative activity of compounds **18b**–**31b**.

Compound	IC50 (uM) ± SEM		
	HL-60	MCF-7	HUVEC
**18b**	>100	>100	
**19b**	>100	>100	
**20b**	8.67 ± 0.14	37.00 ± 4.25	9.92 ± 0.07
**21b**	10.10 ± 0.49	>100	
**22b**	>100	>100	
**23b**	>100	>100	
**24b**	>100	>100	
**25b**	>100	>100	
**26b**	>100	>100	
**27b**	>100	>100	
**28b**	20.00 ± 0.16	>100	
**29b**	21.75 ± 2.08	>100	
**30b**	12.40 ± 0.33	>100	18.95 ± 0.20
**31b**	13.00 ± 0.41	>100	>100

## Supplementary Materials

The following supporting information can be downloaded at: https://www.mdpi.com/article/10.3390/ma15062068/s1, 1. ^1^H, ^13^C and ^77^Se NMR spectra of benzisoselenazolo-3(2*H*)-ones **18a**–**31a**; 2. ^1^H, ^13^C and ^77^Se NMR spectra of diselenides **18b**–**31b**; 3. Crystallographic data of benzisoselenazolo-3(2*H*)-ones **24a** and **25a**.
