# Supplementary material for: Attachment of Chiral Functional Groups to Modify the Activity of New GPx Mimetics
Source: Materials (Basel). 2022 Mar 11;15(6):2068. doi: 10.3390/ma15062068 (PMC8950439; doi:10.3390/ma15062068)
Supplement: Supplementary file 1 [file materials-15-02068-s001.zip › materials-1627357-supplementary.pdf]

materials

MDPI

Article

Attachment of Chiral Functional Groups to Modify the Activity of New GPx Mimetics

Supplemental Information

Anna Laskowska [1], Agata Joanna Pacuła-Miszewska [1], Angelika Długosz-Pokorska [2], Anna Janecka [2], Andrzej Wojtczak [3] and Jacek Ścianowski [1,*]

[1] Department of Organic Chemistry, Faculty of Chemistry, Nicolaus Copernicus University, 7 Gagarin Street, 87-100 Torun, Poland; annlas@doktorant.umk.pl (A.L.); pacula@umk.pl (A.J.P.-M.)
[2] Department of Biomolecular Chemistry, Faculty of Medicine, Medical University of Lodz, Mazowiecka 6/8, 92-215 Lodz, Poland; angelika.dlugosz@umed.lodz.pl (A.D.-P.); anna.janecka@umed.lodz.pl (A.J.)
[3] Department of Crystallochemistry and Biocrystallography, Faculty of Chemistry, Nicolaus Copernicus University in Torun, 7 Gagarin Street, 87-100 Torun, Poland; awojt@umk.pl
* Correspondence: jsch@umk.pl

Table of content

1. ^{1}H, ^{13}C and ^{77}Se NMR spectra of benzisoselenazol-3(2*H*)-ones **18a-31a**, pages 1-22
2. ^{1}H, ^{13}C and ^{77}Se NMR spectra of diselenides **18b-31b**, pages 22-43
3. Crystallographic data of benzisoselenazol-3(2*H*)-ones **24a** and **25a**, pages 43-71

1. NMR spectra of benzisoselenazol-3(2*H*)-ones 18a-31a

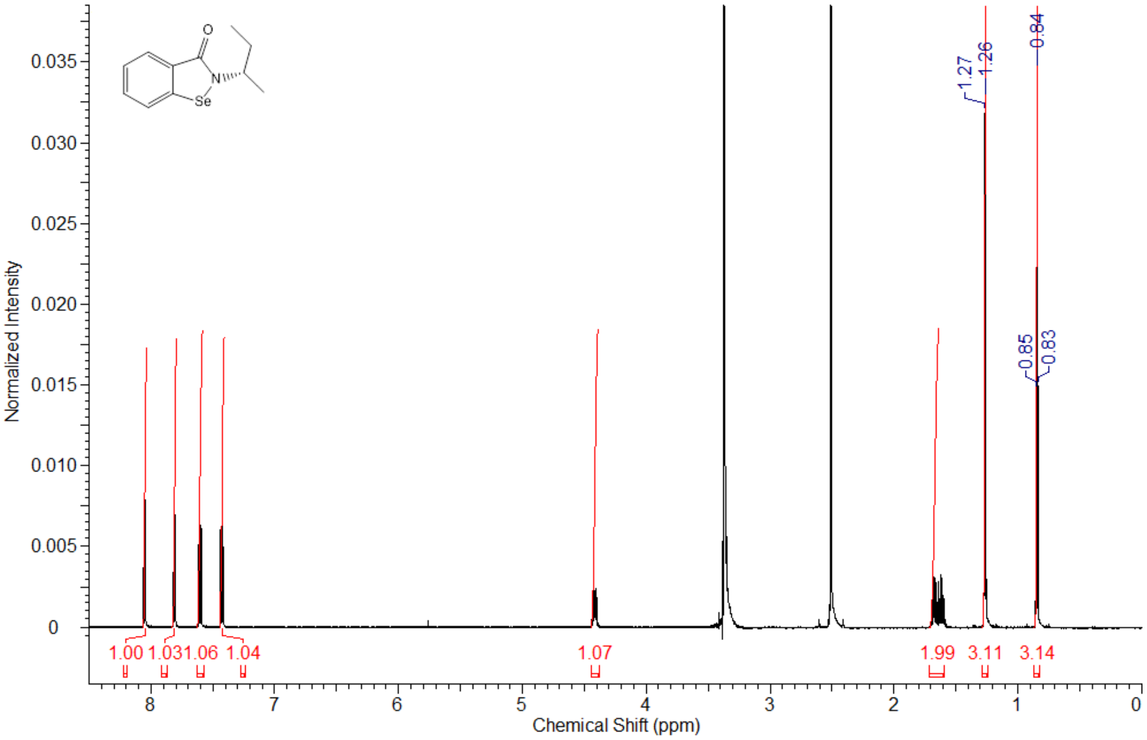

(a)

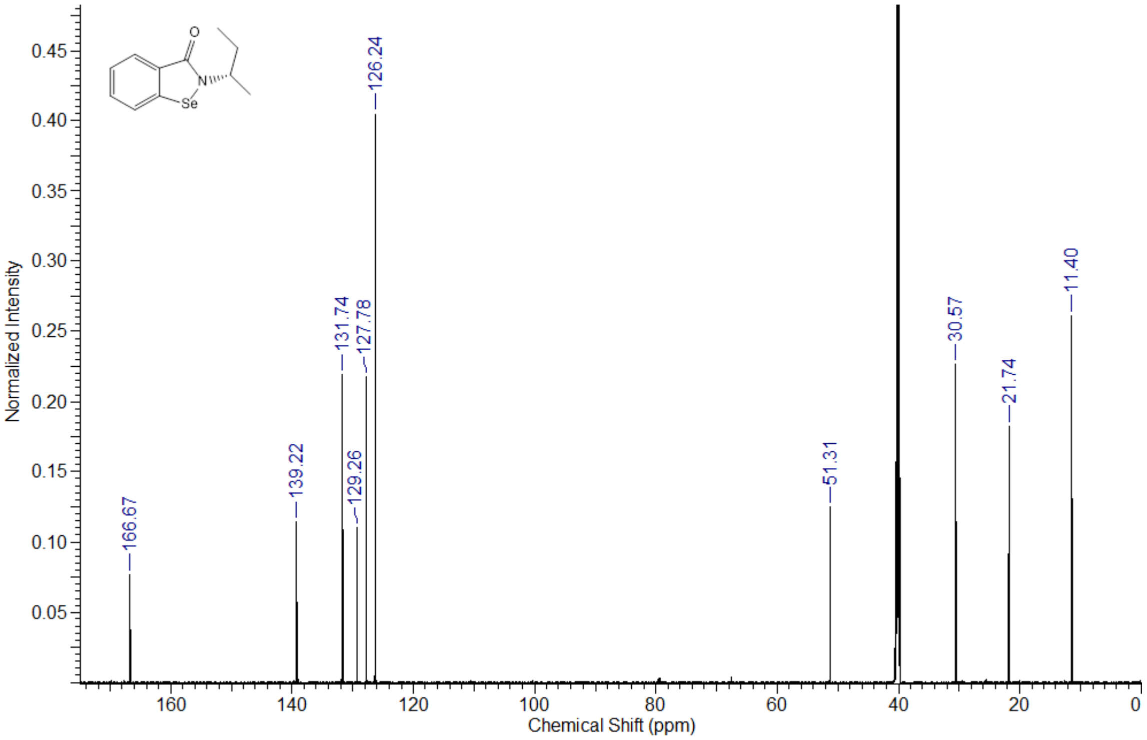

(b)

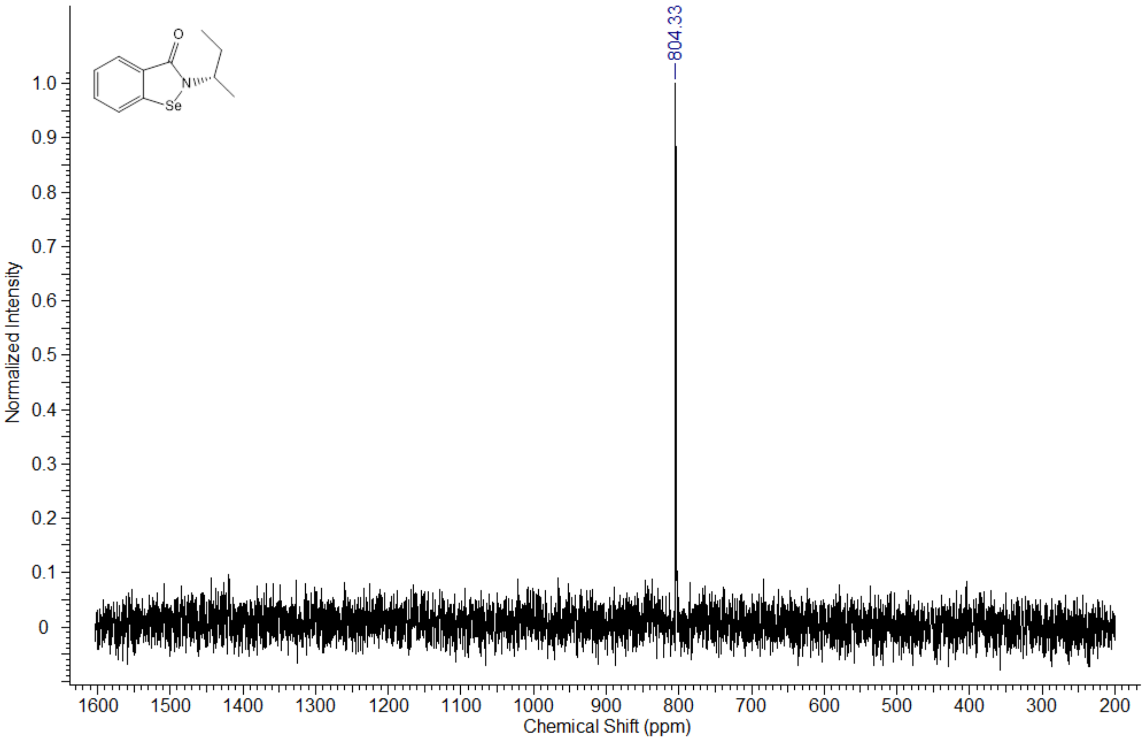

(c)

Figure S1. (**a**) 1H NMR, (**b**) 13C NMR, and (**c**) 77Se NMR spectra of *N*-[(*S*)-(+)-*sec*-butyl]-1,2-benzisoselenazol-3(2*H*)-one **18a.**

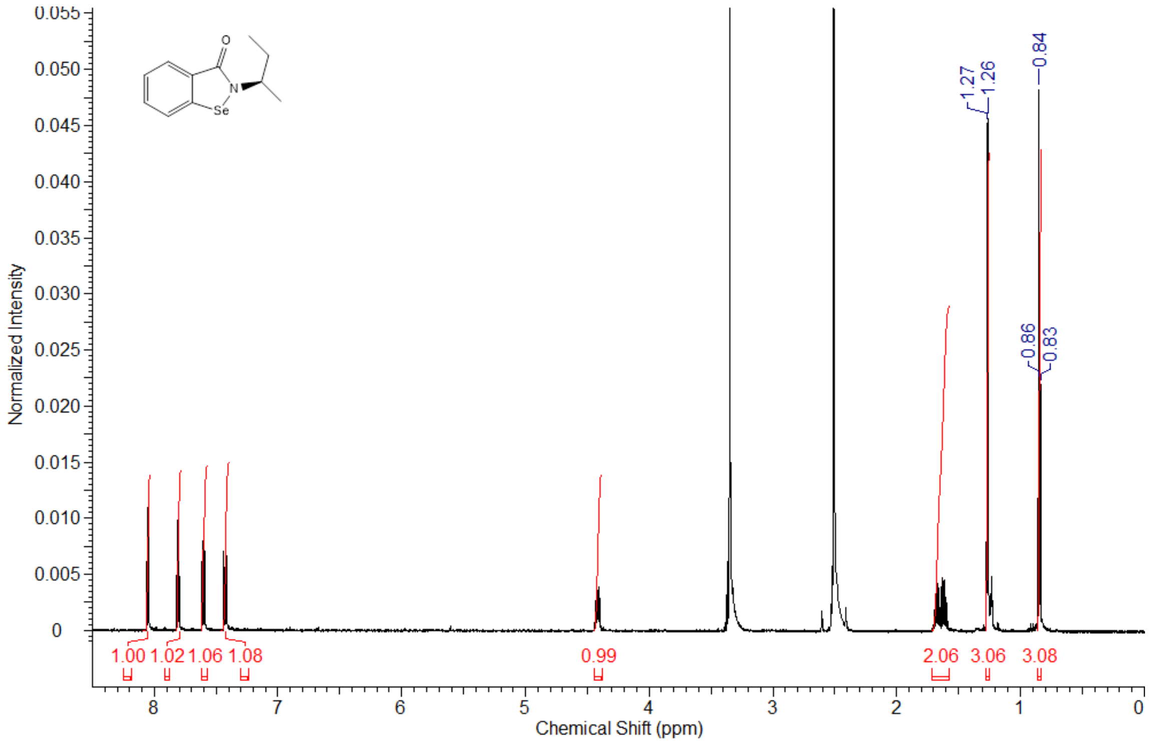
Normalized Intensity
Chemical Shift (ppm)
1.27
1.26
0.84
0.86
0.83
1.00 1.02 1.06 1.08
0.99
2.06 3.06 3.08

(a)

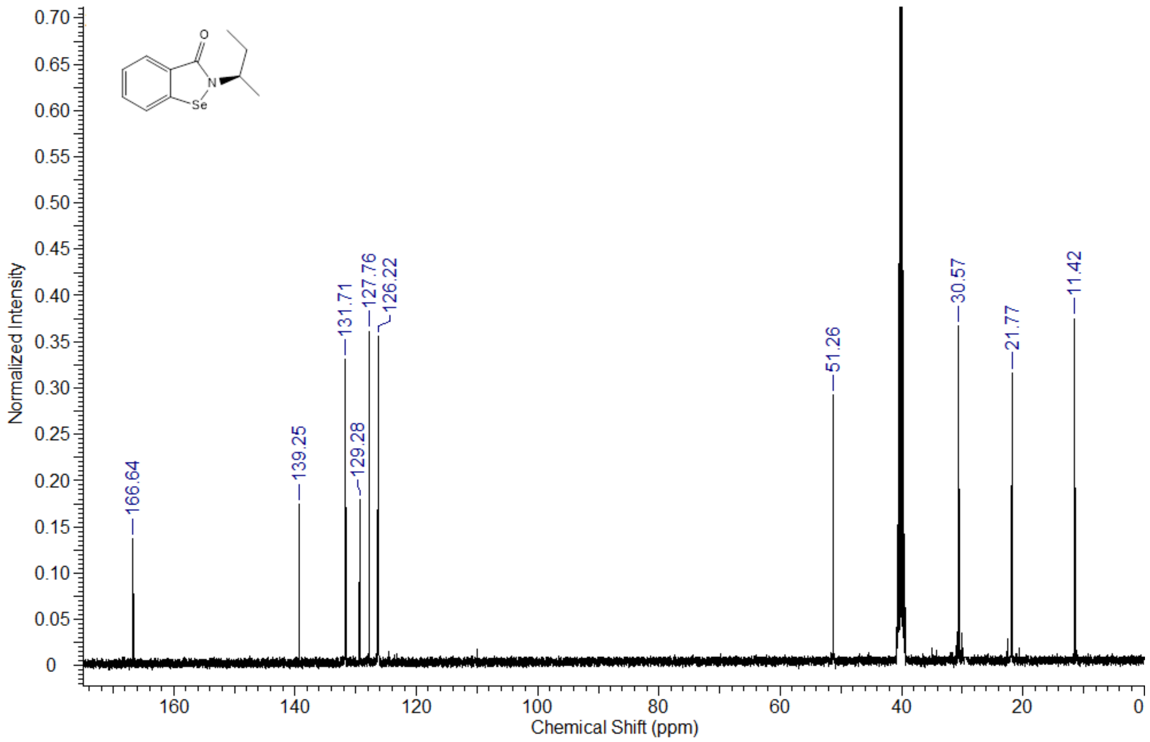
Normalized Intensity
Chemical Shift (ppm)
166.64
139.25
131.71
129.28
127.76
126.22
51.26
30.57
21.77
11.42

(b).

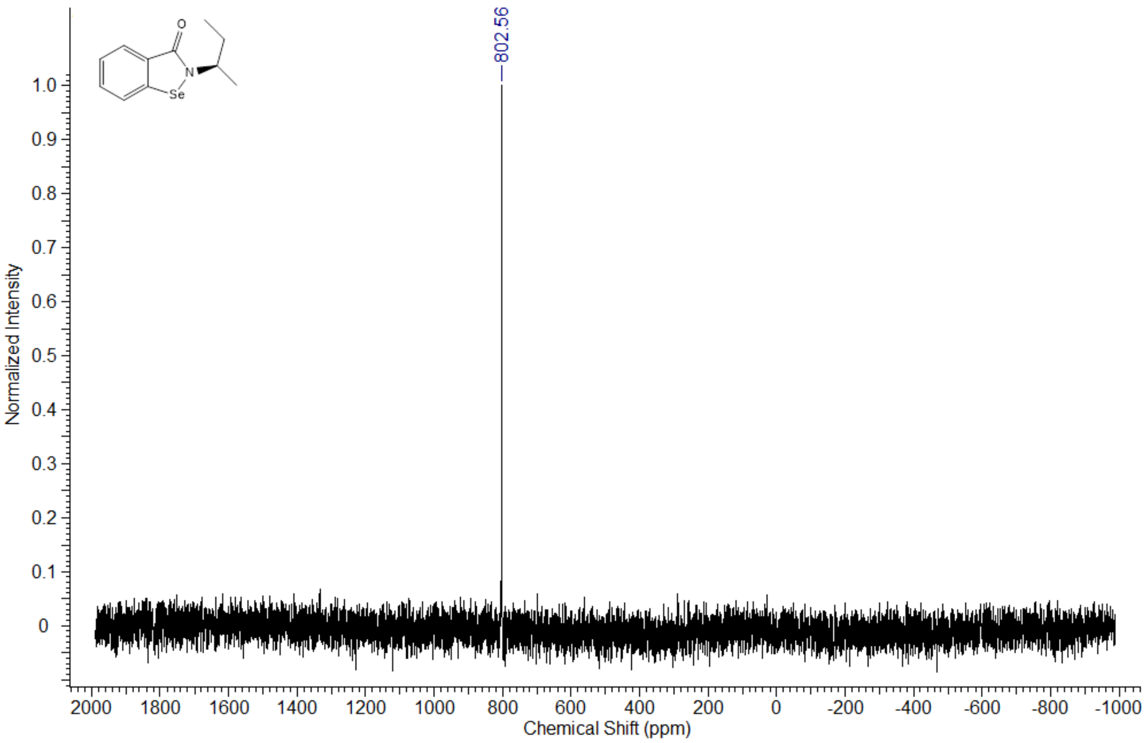

(c)

Figure S2. (**a**) 1H NMR, (**b**) 13C NMR, and (**c**) 77Se NMR spectra of *N*-[(*R*)-(-)-*sec*-butyl]-1,2-benzisoselenazol-3(2*H*)-one **19a.**

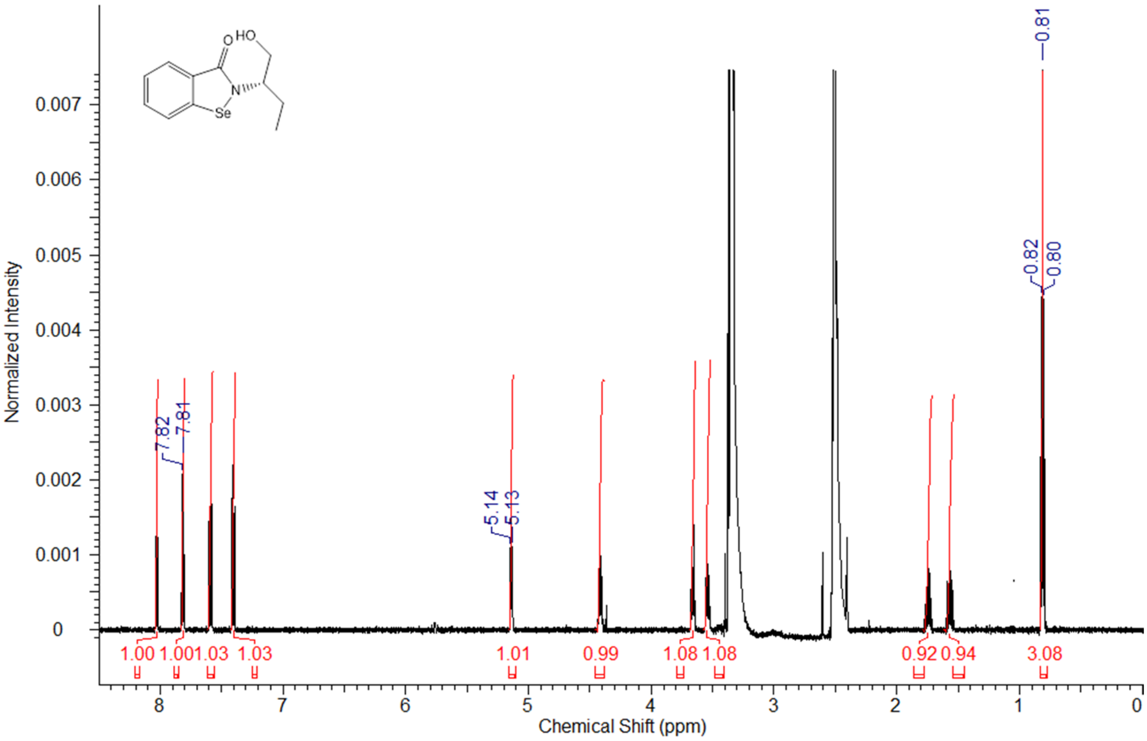

(a)

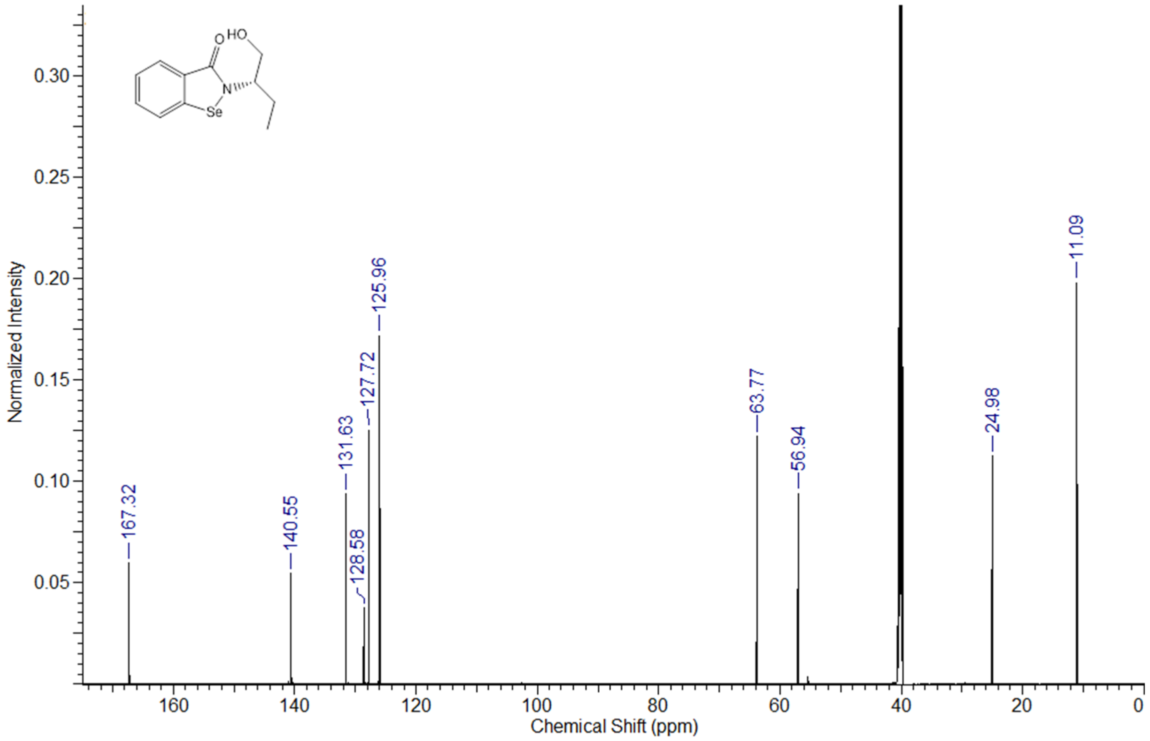

(b)

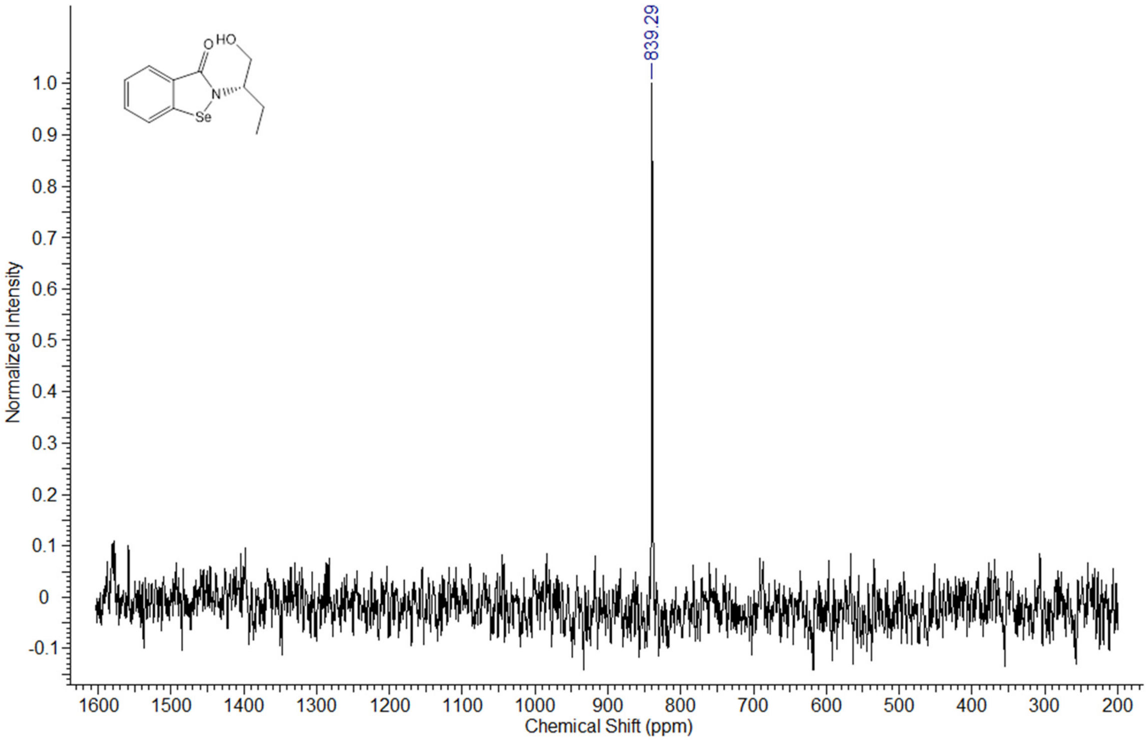

(c)

Figure S3. (**a**) 1H NMR, (**b**) 13C NMR, and (**c**) 77Se NMR spectra of *N*-[(*S*)-(+)-1-hydroxy-2-butanyl]-1,2-benzisoselenazol-3(2*H*)-one **20a.**

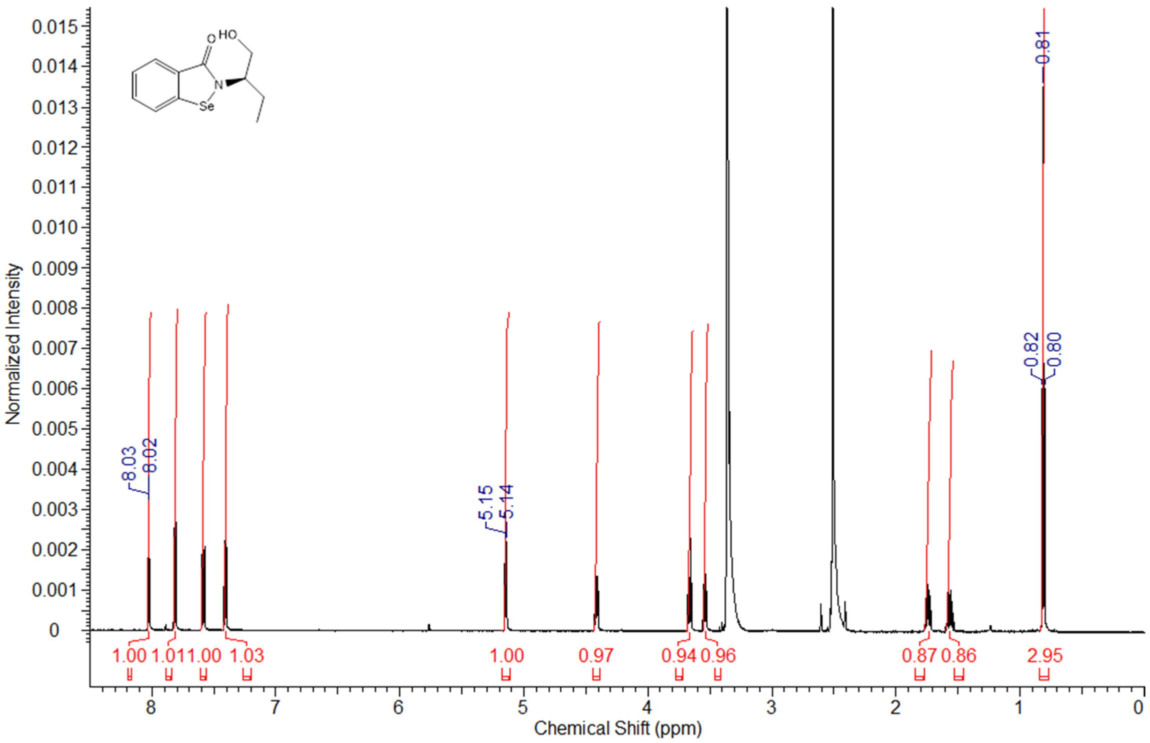

Normalized Intensity
Chemical Shift (ppm)
8.03
8.02
5.15
5.14
0.81
0.82
0.80
1.00 1.01 1.00 1.03
1.00
0.97
0.94 0.96
0.87 0.86
2.95

(a)

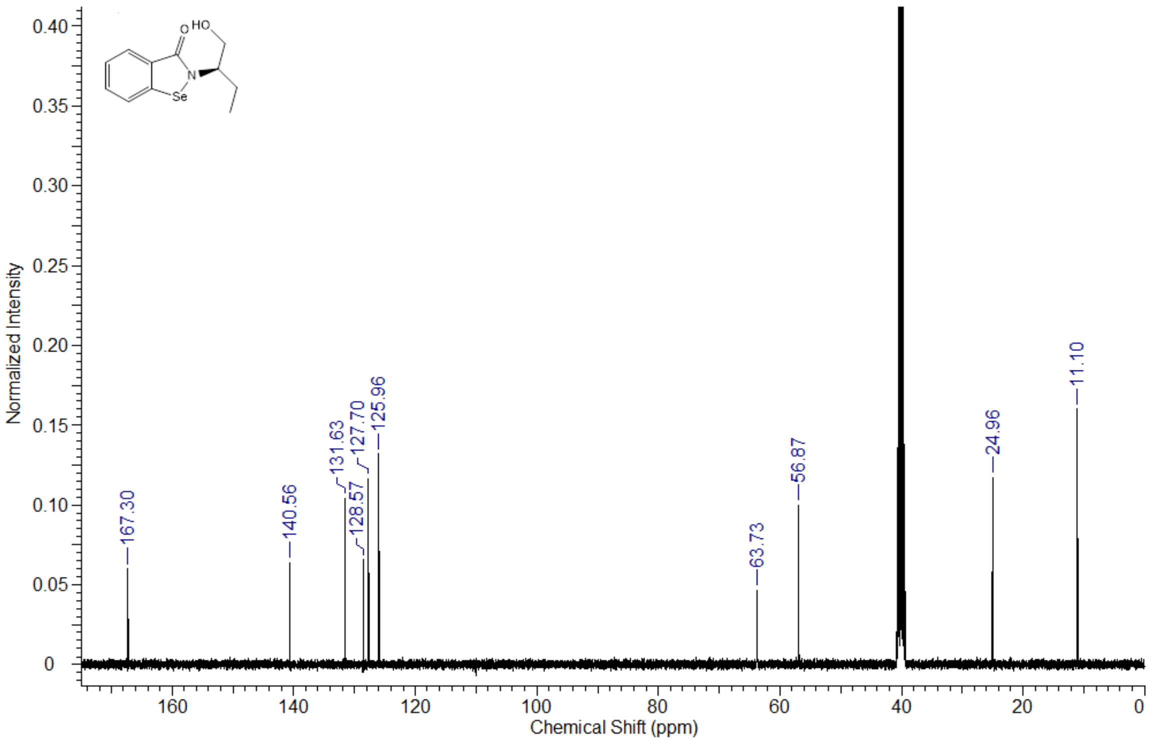

Normalized Intensity
Chemical Shift (ppm)
167.30
140.56
131.63
128.57
127.70
125.96
63.73
56.87
24.96
11.10

(b)

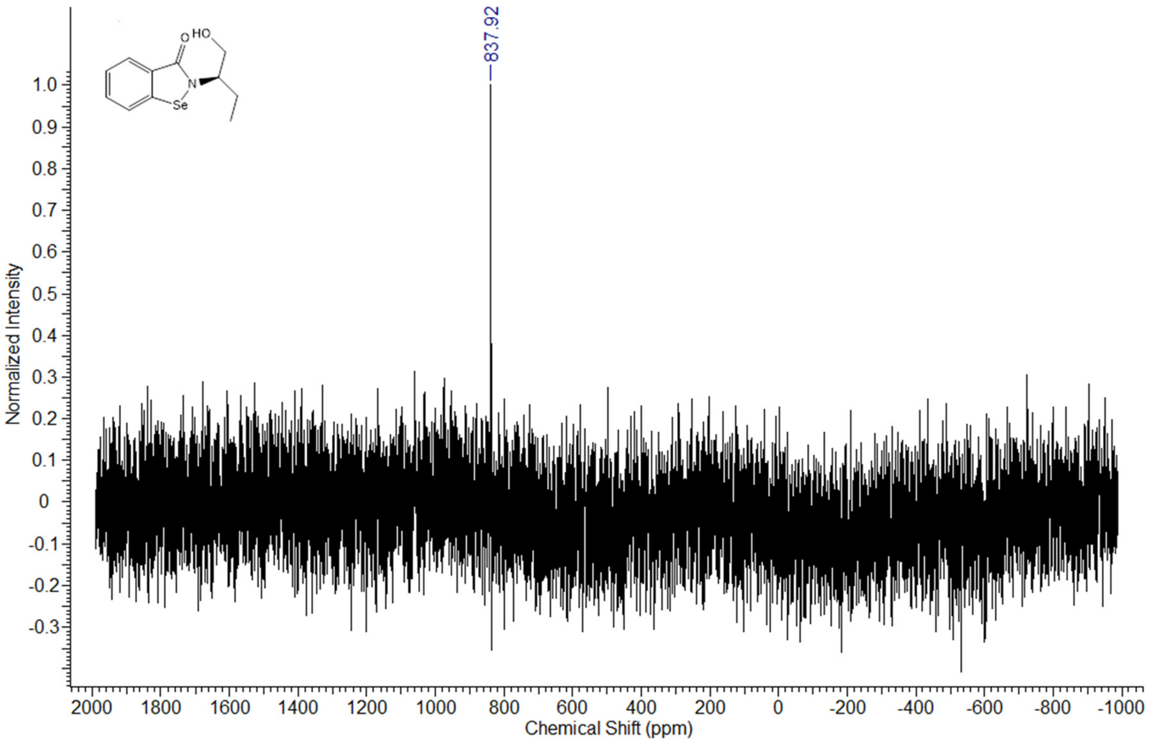

(c)

Figure S4. (**a**) 1H NMR, (**b**) 13C NMR, and (**c**) 77Se NMR spectra of *N*-[(*R*)-(-)-1-hydroxy-2-butanyl]-1,2-benzisoselenazol-3(2*H*)-one **21a.**

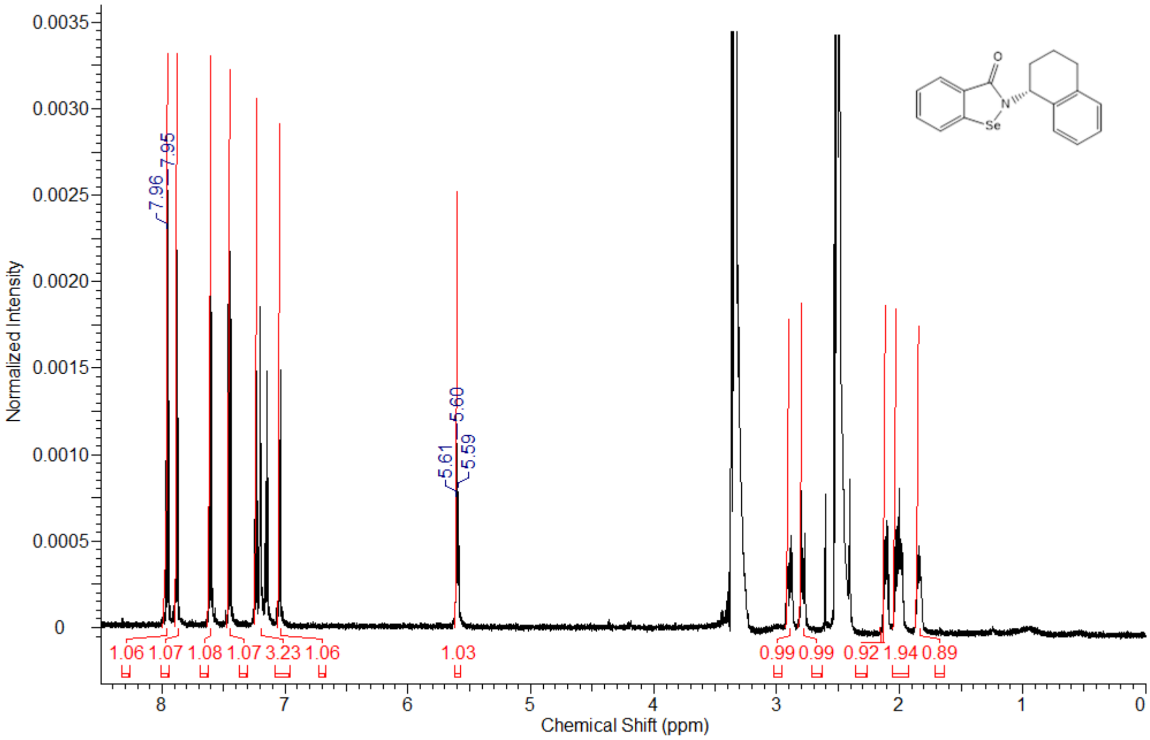

(a)

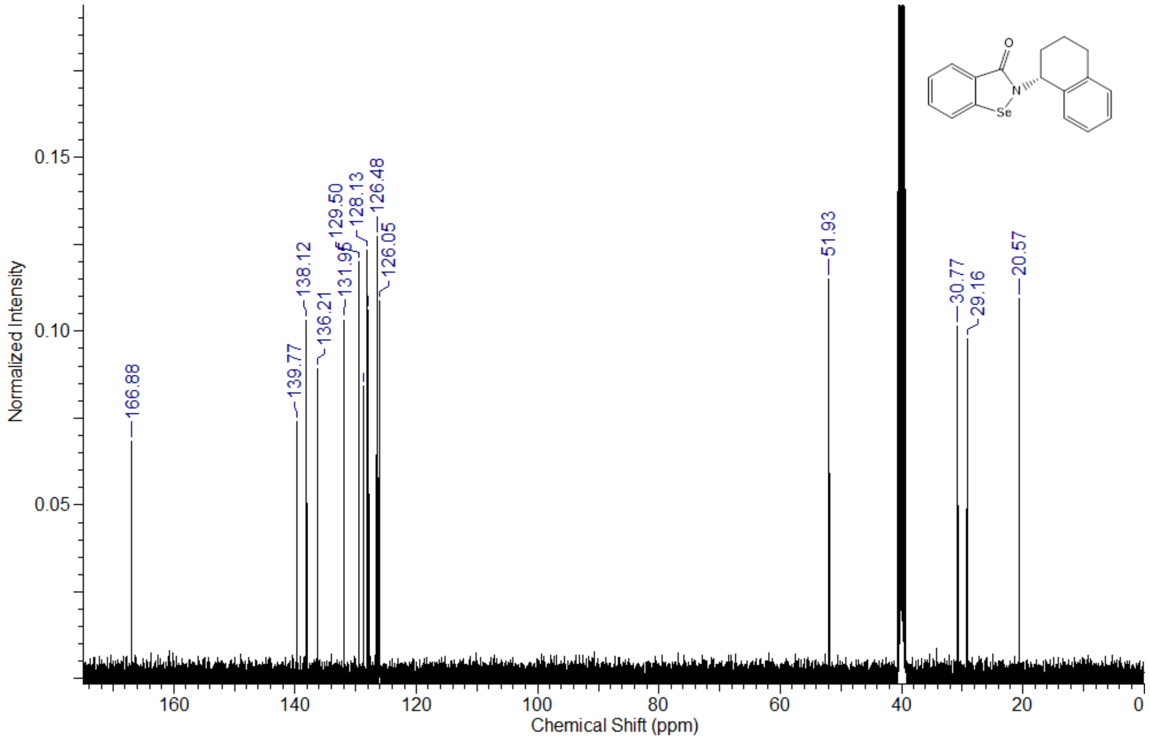

(b)

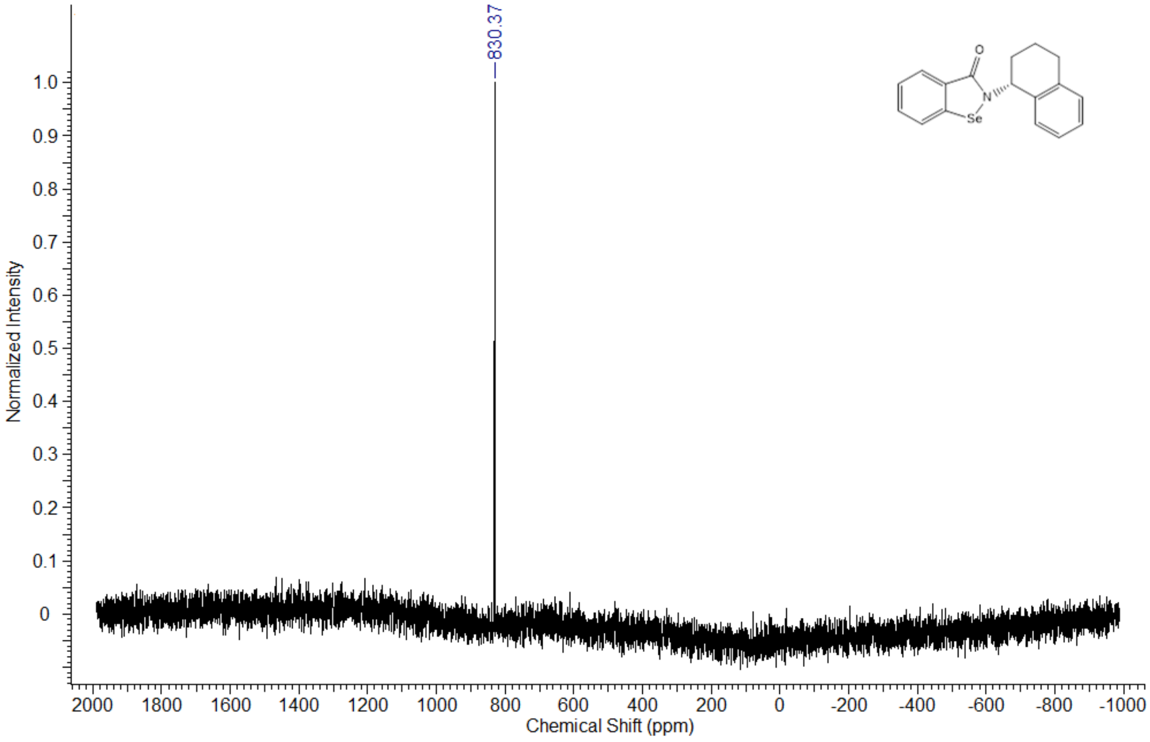

(c)

Figure S5. (**a**) 1H NMR, (**b**) 13C NMR, and (**c**) 77Se NMR spectra of *N*-[(*R*)-(-)-1,2,3,4-tetrahydro-1-napthyl]-1,2-benzisoselenazol-3(2*H*)-one **22a.**

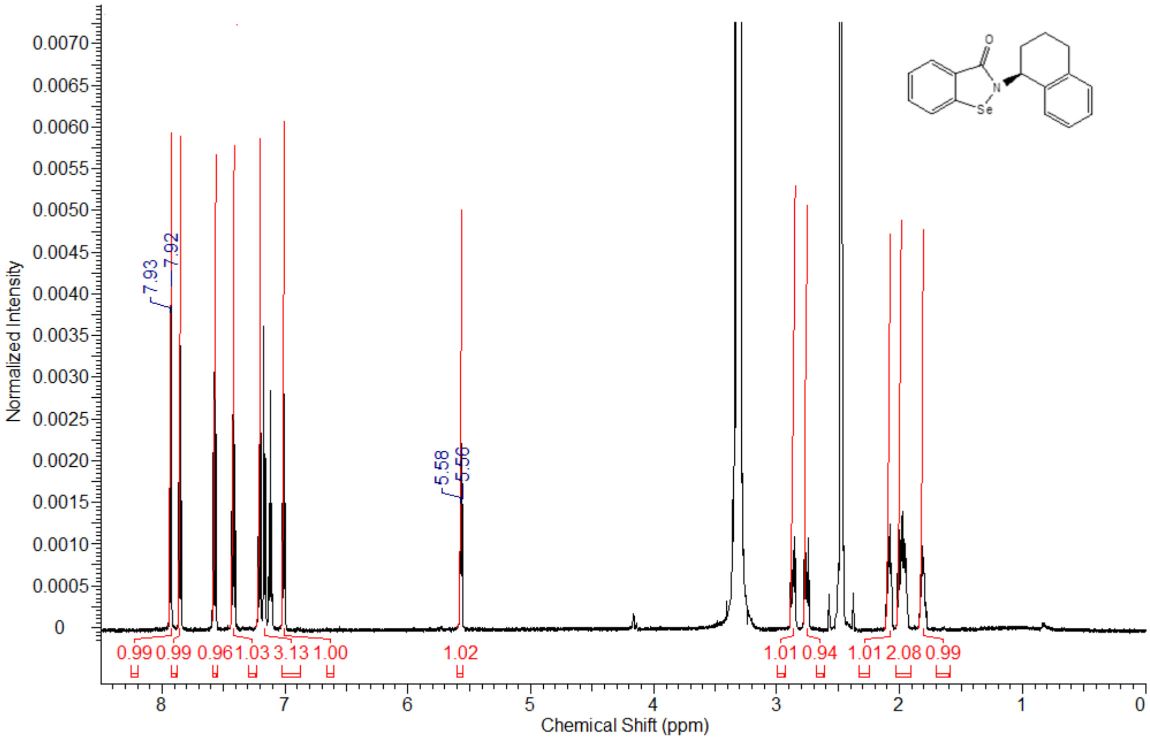
Normalized Intensity
Chemical Shift (ppm)
7.93
7.92
5.58
5.56
0.99 0.99 0.96 1.03 3.13 1.00
1.02
1.01 0.94 1.01 2.08 0.99
O
N
Se

(a)

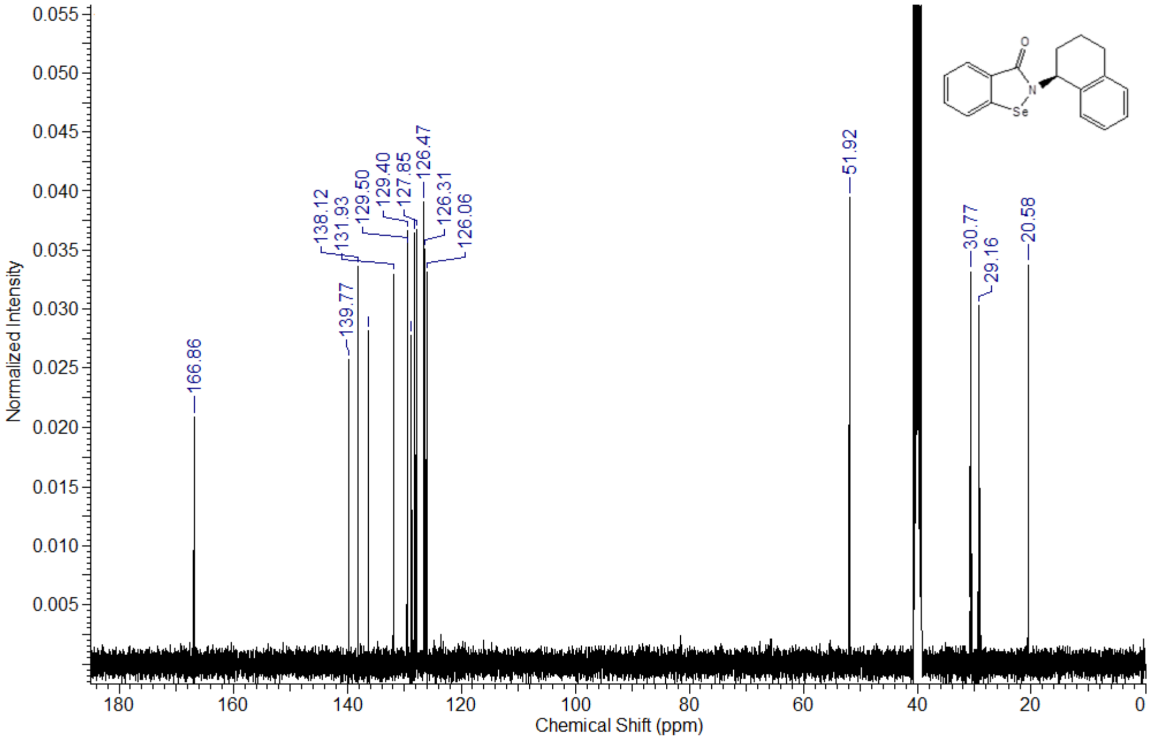
Normalized Intensity
Chemical Shift (ppm)
166.86
139.77
138.12
131.93
129.50
129.40
127.85
126.47
126.31
126.06
51.92
30.77
29.16
20.58
O
N
Se

(b)

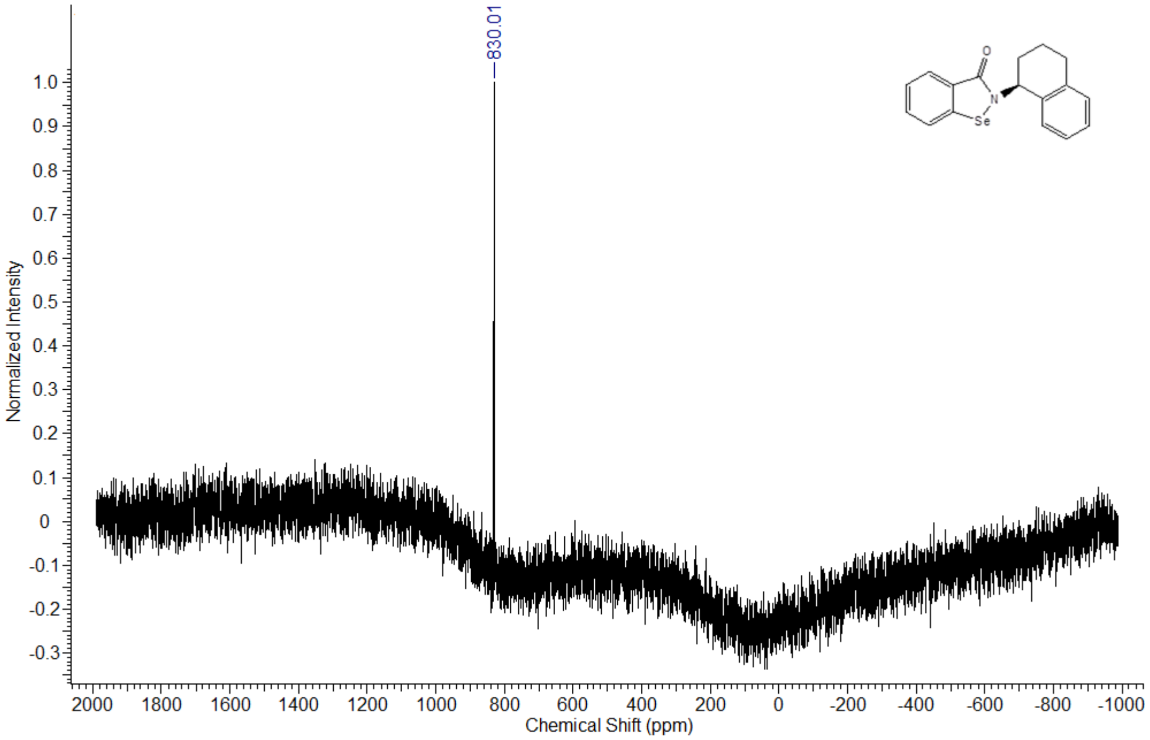

(c)

Figure S6. (**a**) 1H NMR, (**b**) 13C NMR, and (**c**) 77Se NMR spectra of *N*-[(*S*)-(+)-1,2,3,4-tetrahydro-1-napthyl]-1,2-benzisoselenazol-3(2*H*)-one **23a.**

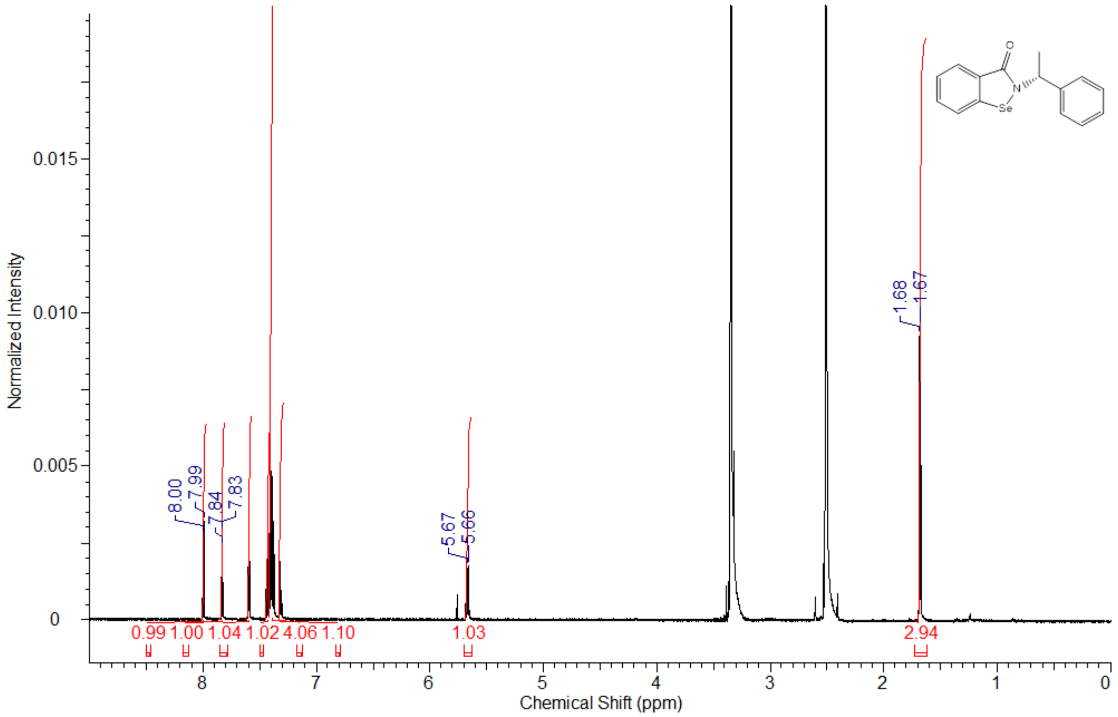

(a)

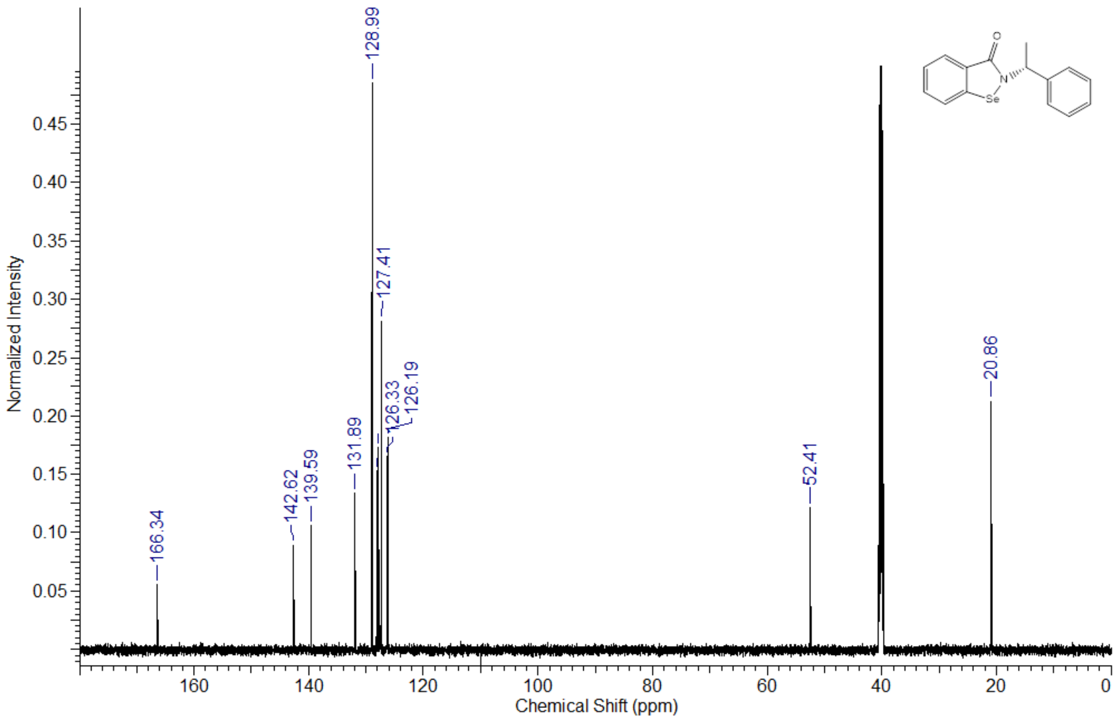

(b)

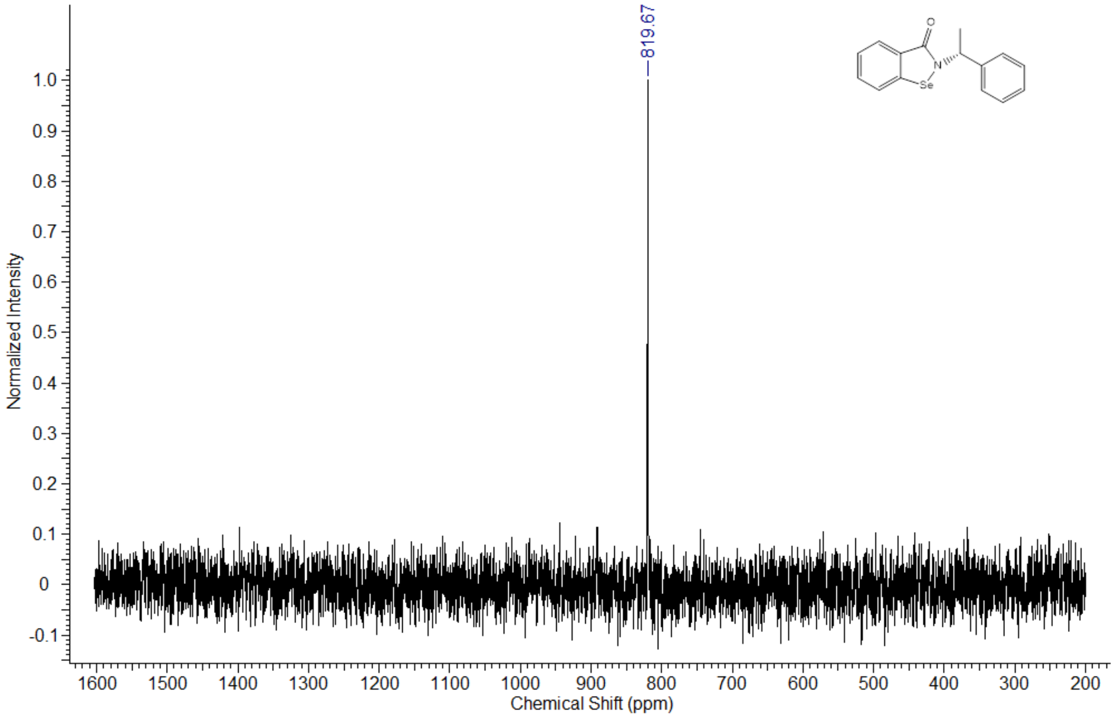

(c)

Figure S7. (**a**) 1H NMR, (**b**) 13C NMR, and (**c**) 77Se NMR spectra of *N*-[(*R*)-(+)-*α*-methylbenzyl]-1,2-benzisoselenazol-3(2*H*)-one **24a.**

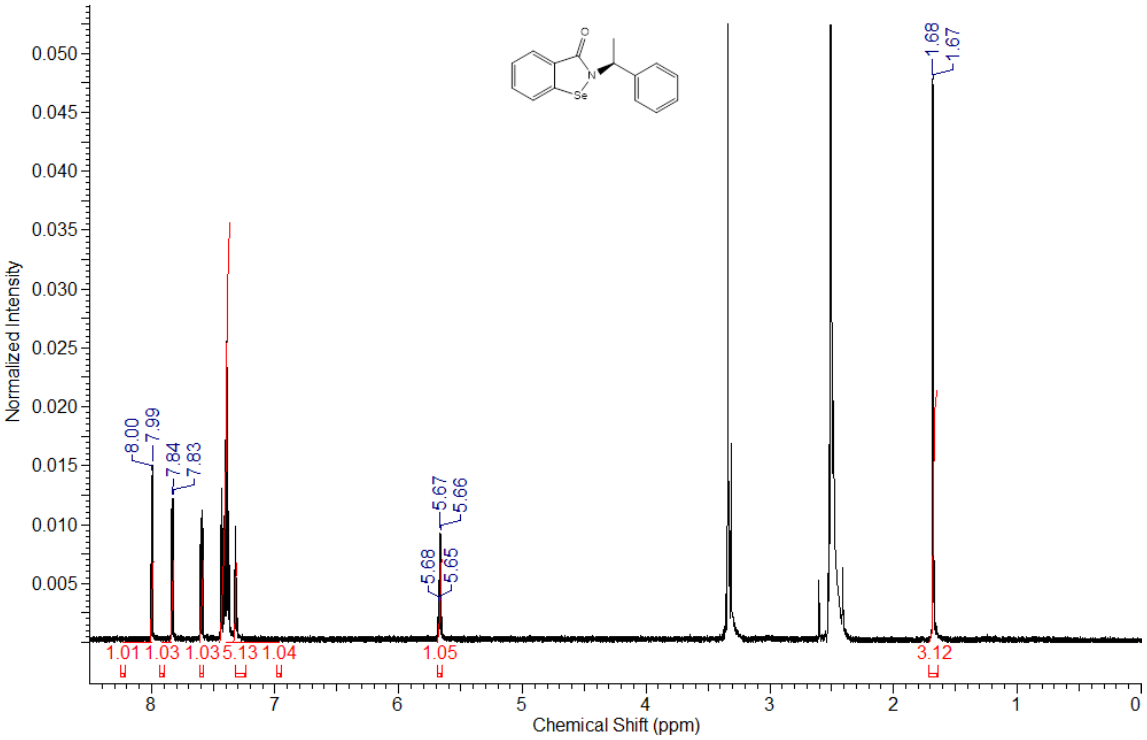
Normalized Intensity
Chemical Shift (ppm)
8.00
7.99
7.84
7.83
5.67
5.66
5.68
5.65
1.68
1.67
1.01 1.03 1.03 5.13 1.04
1.05
3.12
Se
N
O

(a)

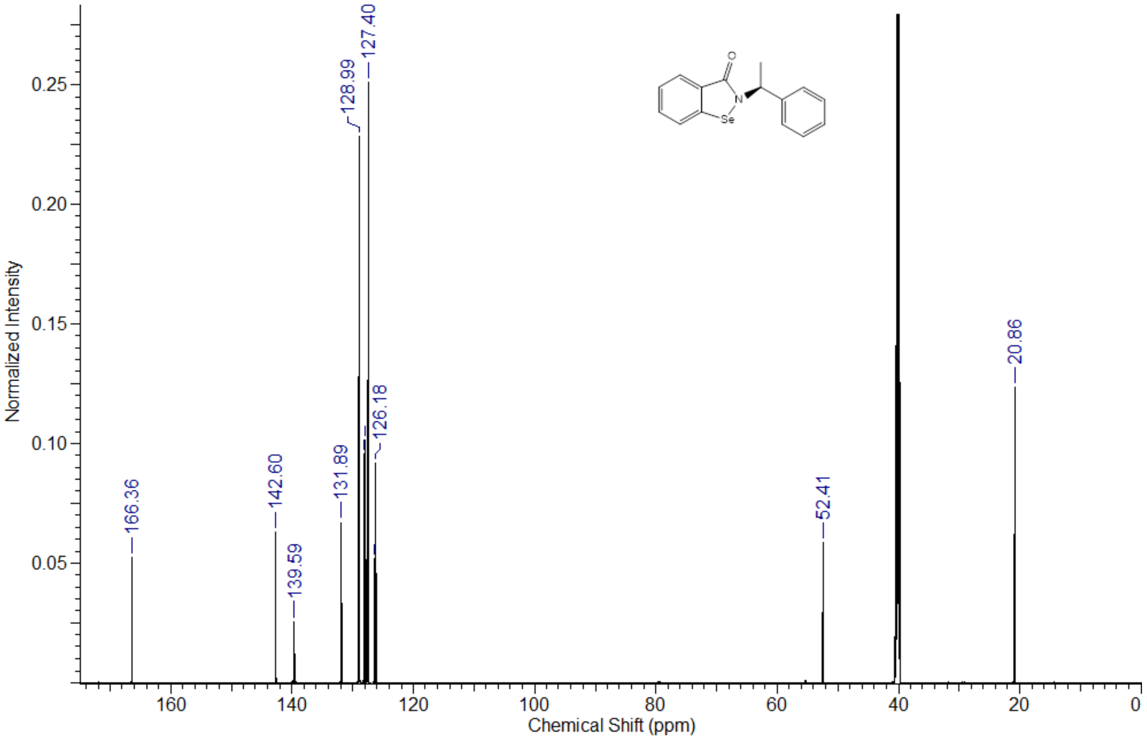
Normalized Intensity
Chemical Shift (ppm)
166.36
142.60
139.59
131.89
128.99
127.40
126.18
52.41
20.86
Se
N
O

(b)

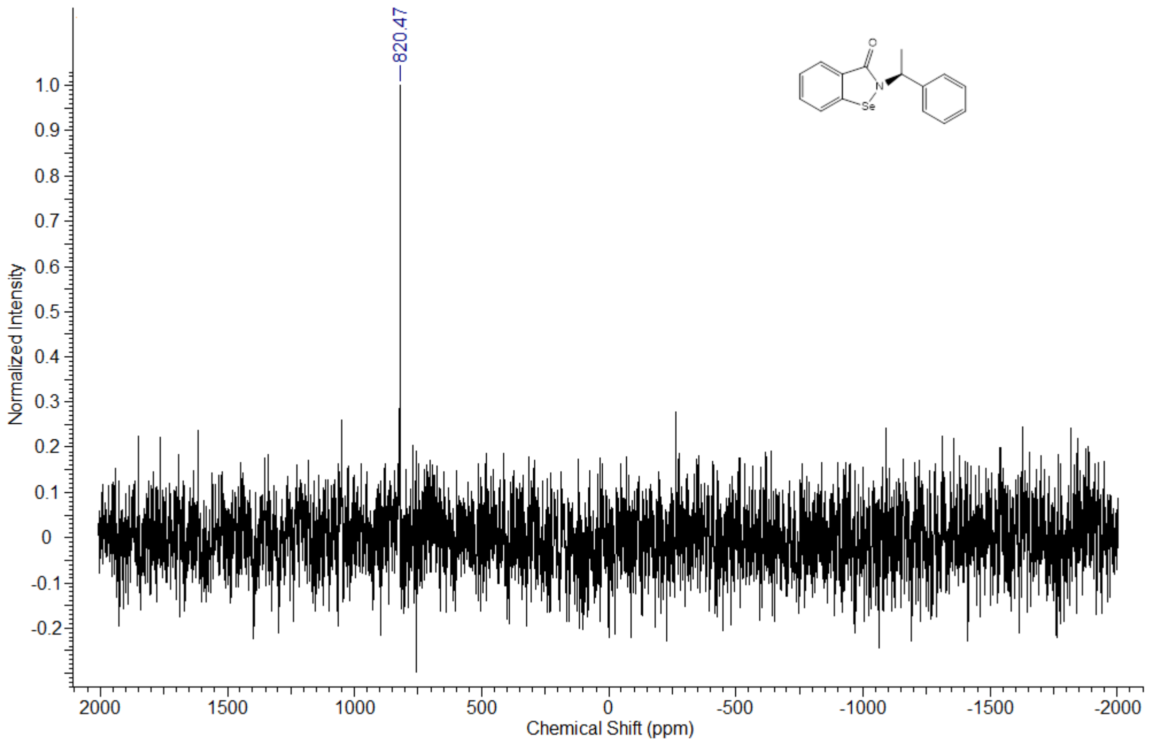

(c)

Figure S8. (**a**) 1H NMR, (**b**) 13C NMR, and (**c**) 77Se NMR spectra of *N*-[(*S*)-(-)-*α*-methylbenzyl]-1,2-benzisoselenazol-3(2*H*)-one **25a.**

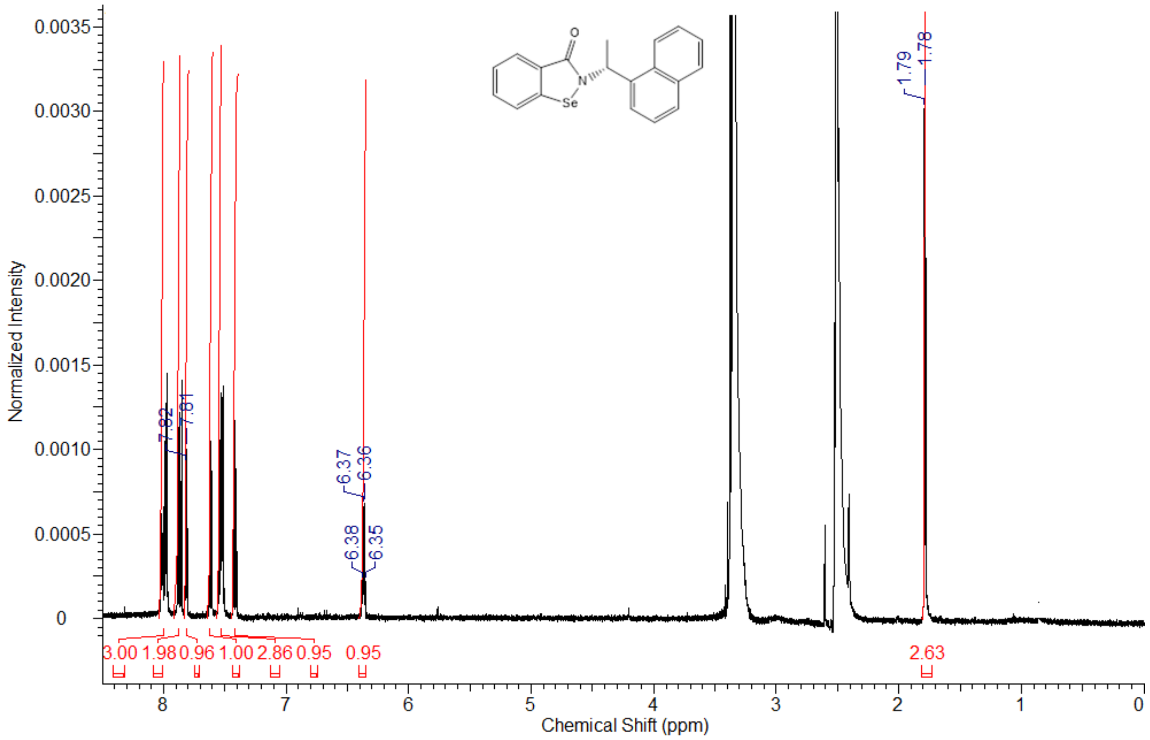

(a)

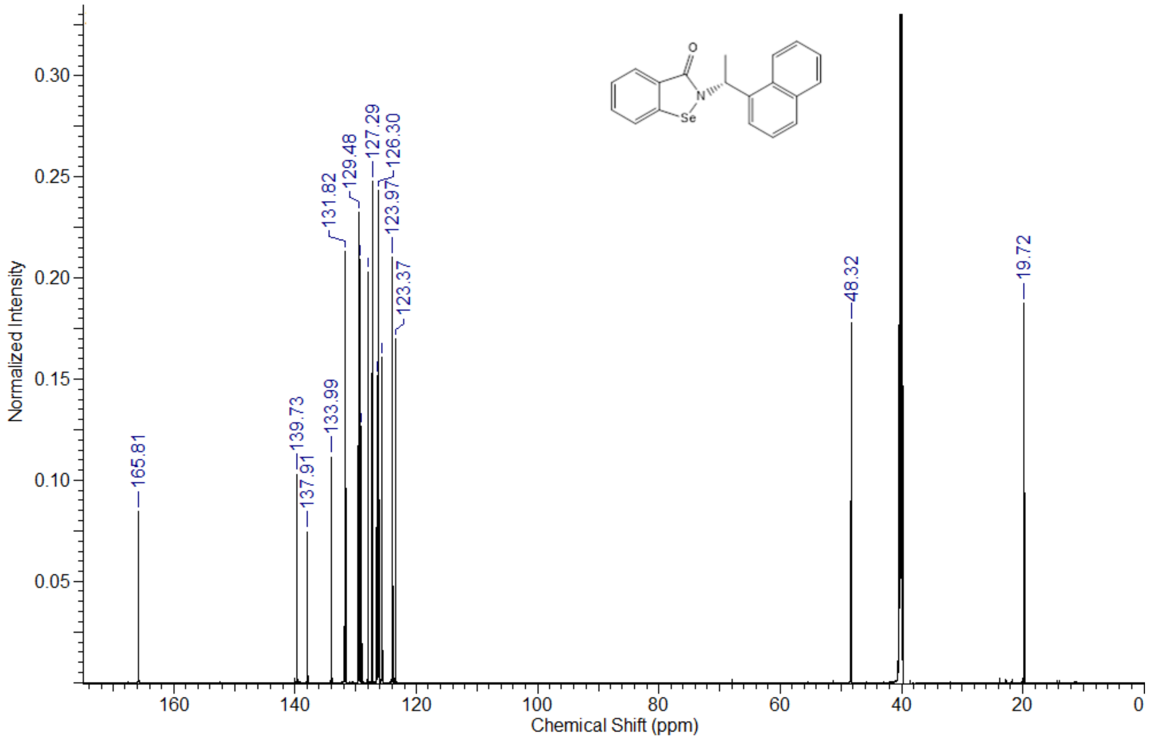

(b)

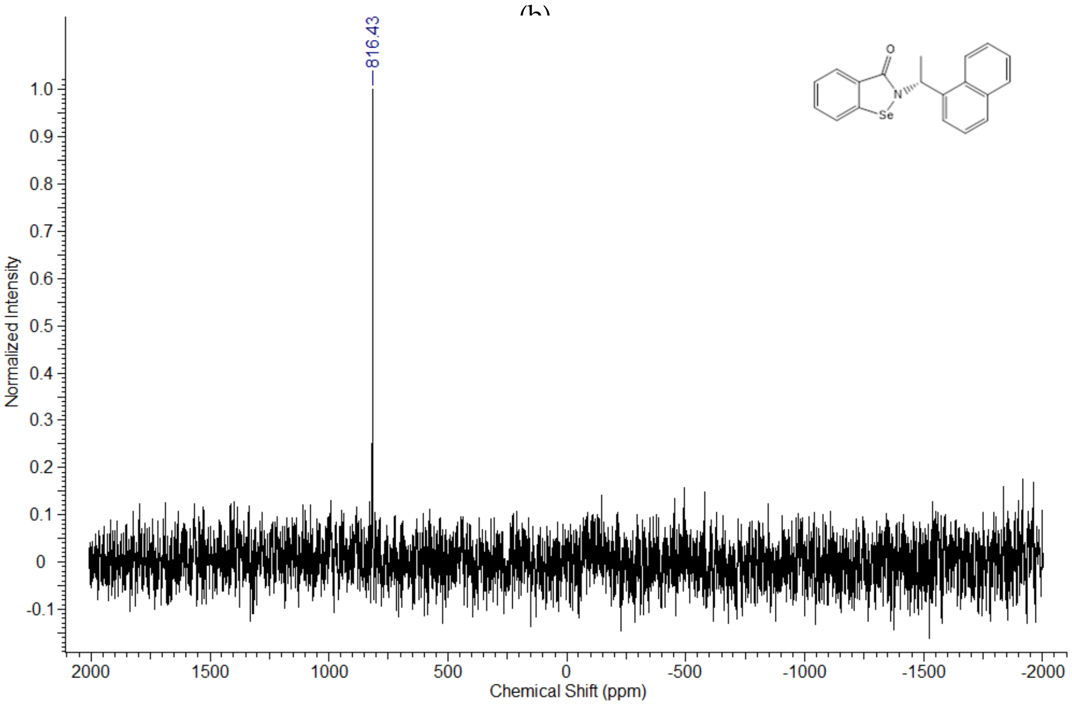

(c)

Figure S9. (**a**) 1H NMR, (**b**) 13C NMR, and (**c**) 77Se NMR spectra of *N*-[(*R*)-(-)-1-(1-napthyl)ethyl]-1,2-benzisoselenazol-3(2*H*)-one **26a**

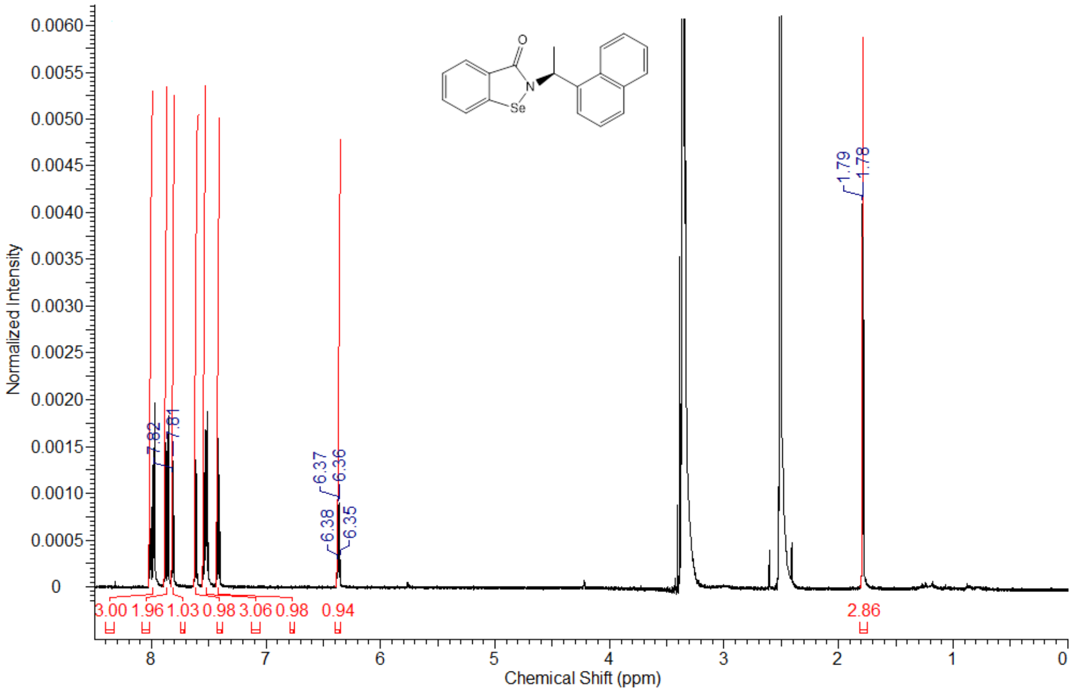

Normalized Intensity
Chemical Shift (ppm)
O
N
Se
7.82
7.81
6.37
6.36
6.38
6.35
1.79
1.78
3.00
1.96
1.03
0.98
3.06
0.98
0.94
2.86

(a)

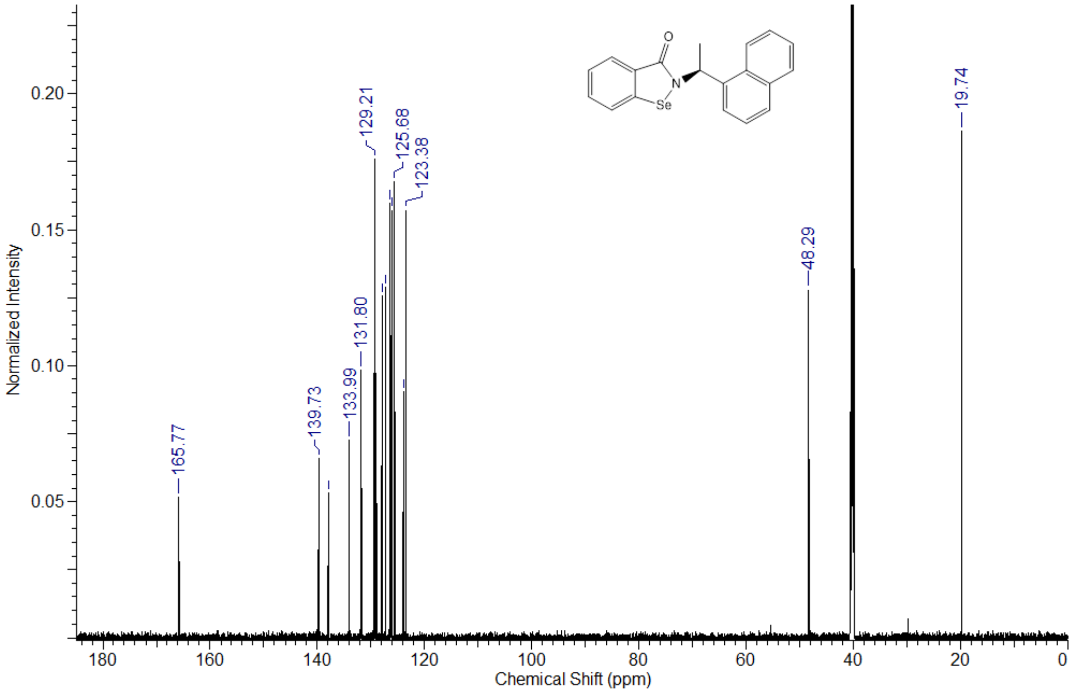

Normalized Intensity
Chemical Shift (ppm)
O
N
Se
165.77
139.73
133.99
131.80
129.21
125.68
123.38
48.29
19.74

(b)

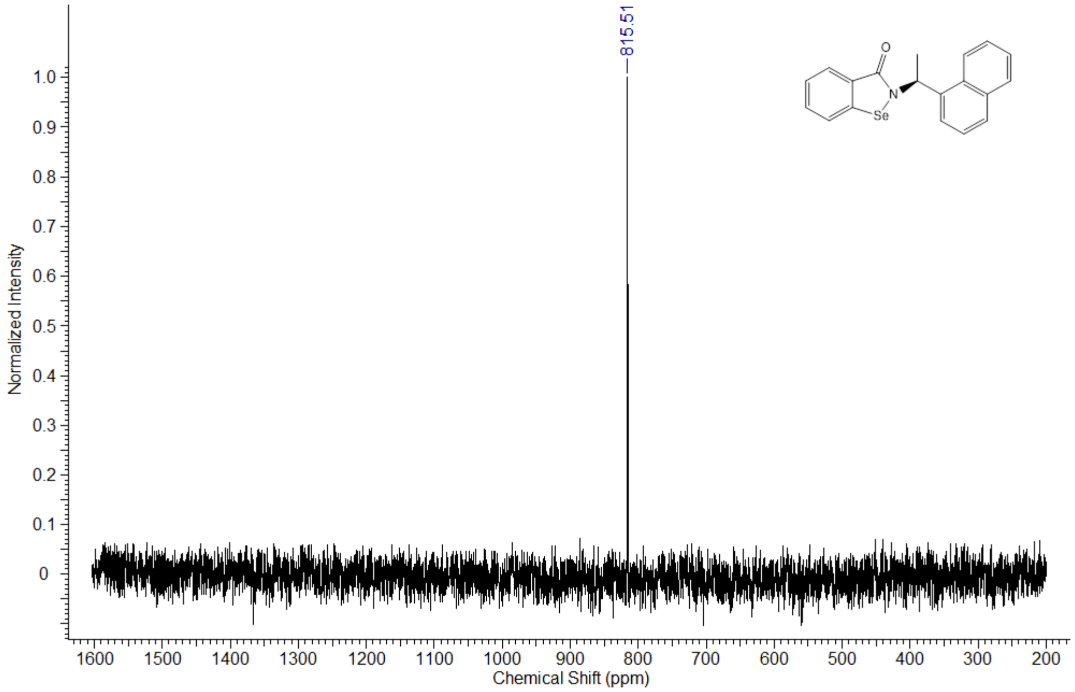

(c)

Figure S10. (**a**) 1H NMR, (**b**) 13C NMR, and (**c**) 77Se NMR spectra of *N*-[(*R*)-(+)-1-(1-napthyl)ethyl]-1,2-benzisoselenazol-3(2*H*)-one **27a.**

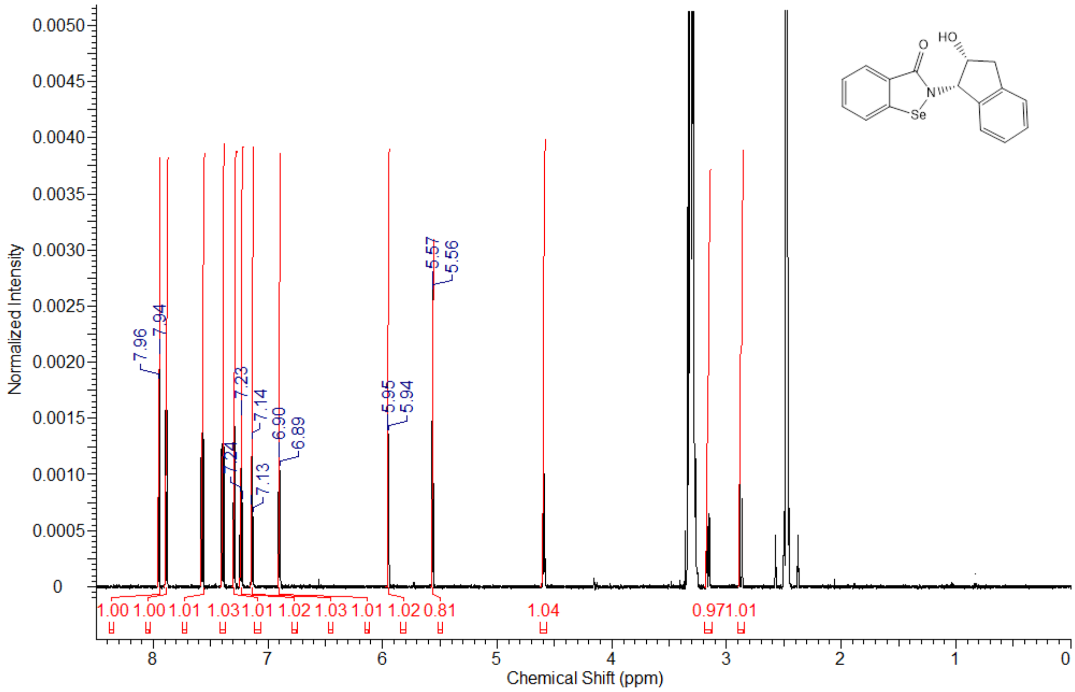

(a)

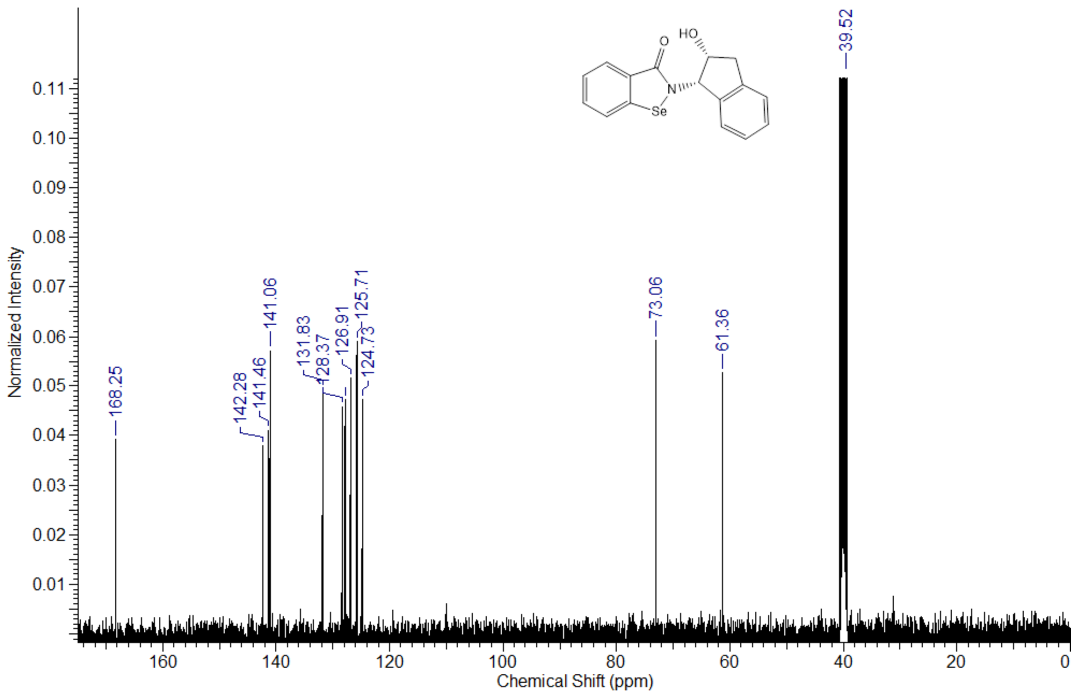

(b)

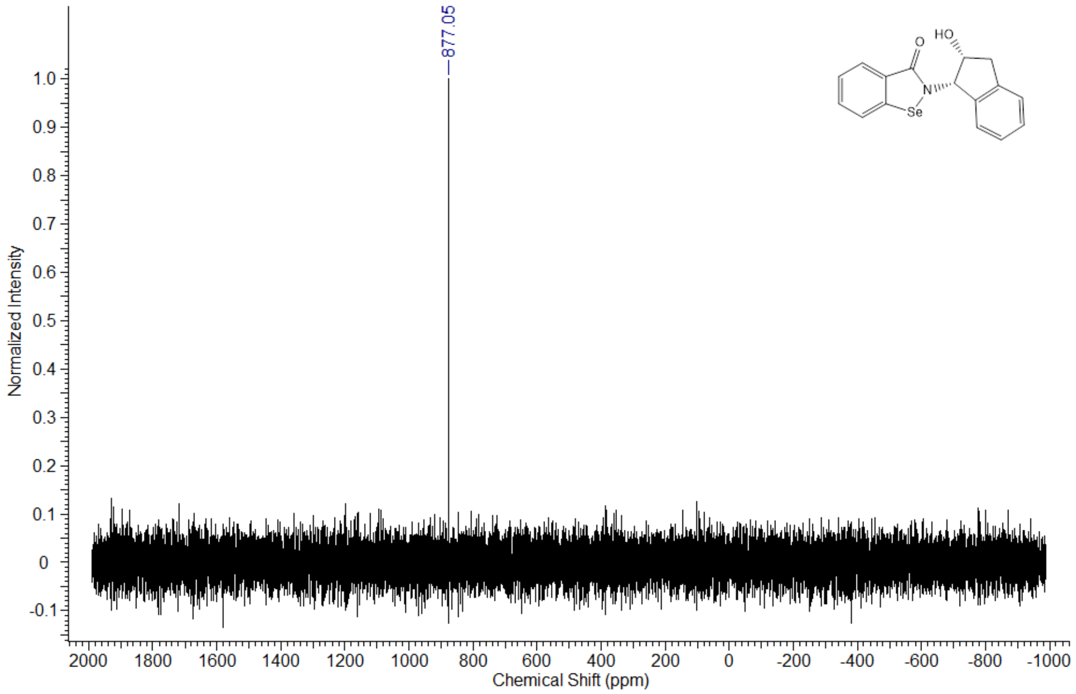

(c)

Figure S11. (**a**) 1H NMR, (**b**) 13C NMR, and (**c**) 77Se NMR spectra of *N*-[(*1S*,*2R*)-(-)-*cis*-2-hydroxy-1-indanyl]-1,2-benzisoselenazol-3(2*H*)-one **28a.**

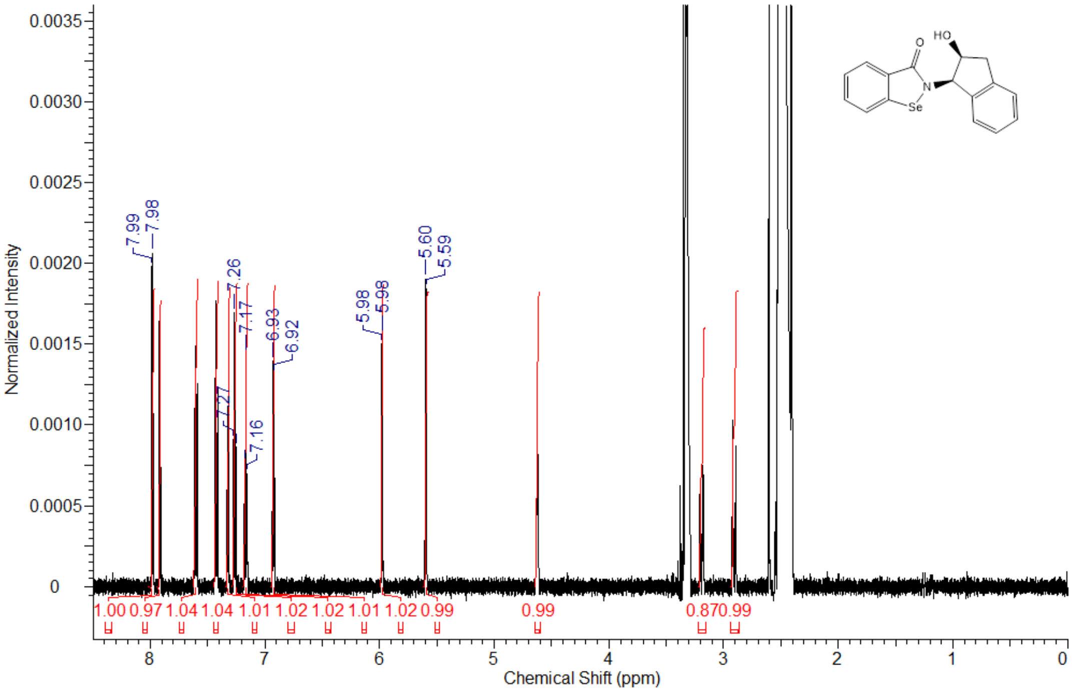
Normalized Intensity
Chemical Shift (ppm)
7.99
7.98
7.26
7.27
7.17
7.16
6.93
6.92
5.98
5.98
5.60
5.59
1.00 0.97 1.04 1.04 1.01 1.02 1.02 1.01 1.02 0.99
0.99
0.87 0.99
HO
O
N
Se

(a)

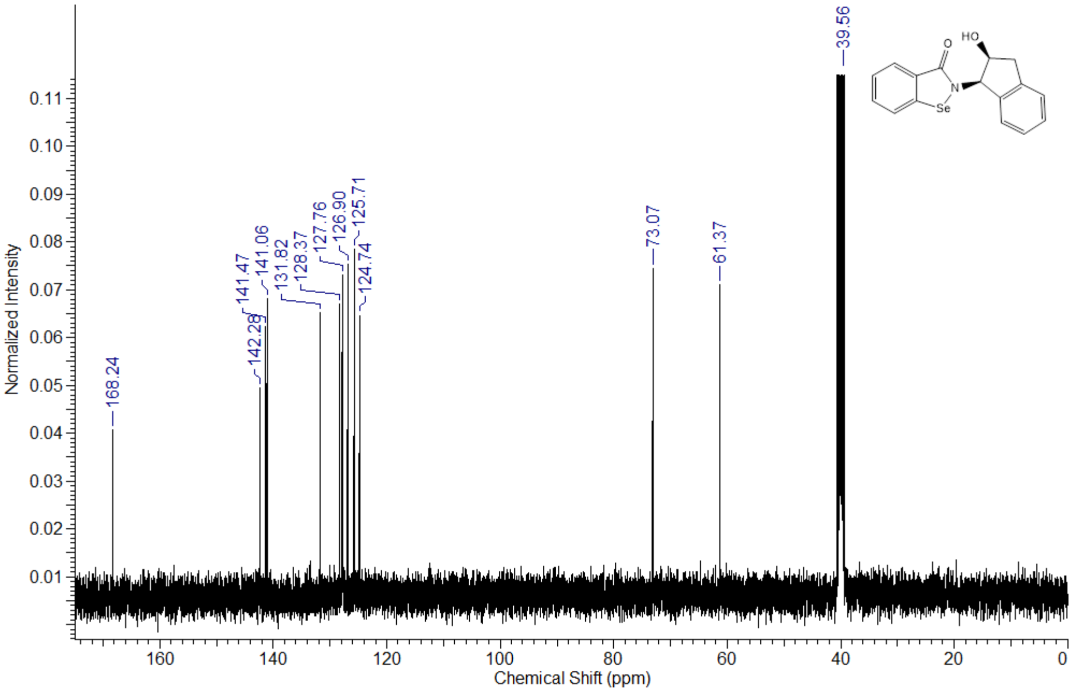
Normalized Intensity
Chemical Shift (ppm)
168.24
142.28
141.47
141.06
131.82
128.37
127.76
126.90
125.71
124.74
73.07
61.37
39.56
HO
O
N
Se

(b)

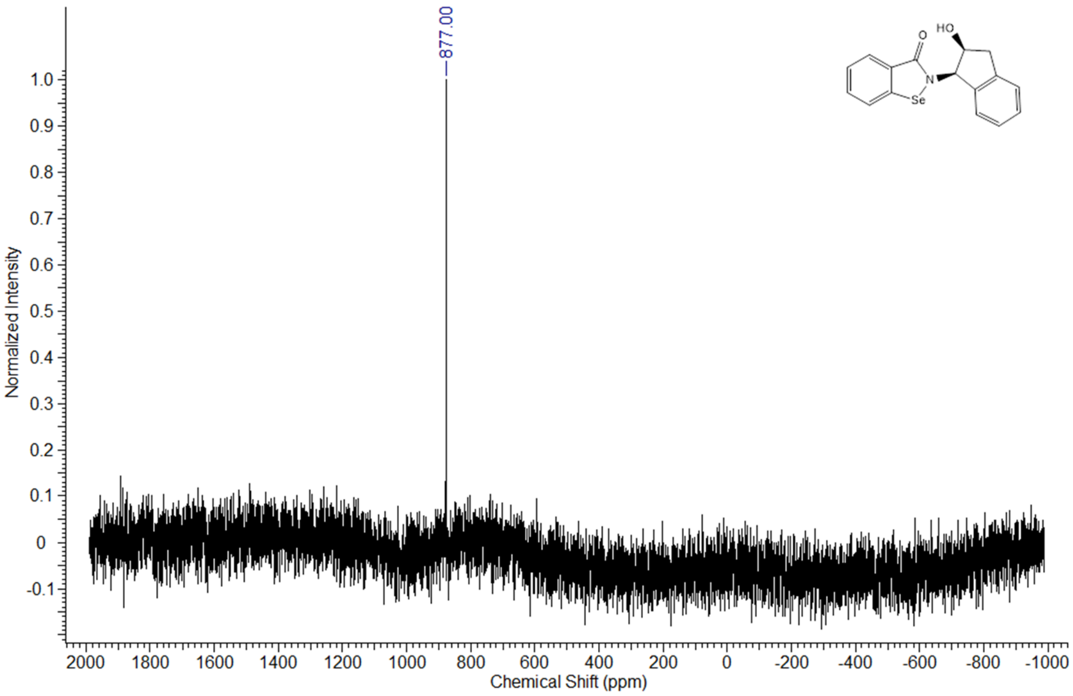

(c)

Figure S12. (**a**) 1H NMR, (**b**) 13C NMR, and (**c**) 77Se NMR spectra of *N*-[(*1R,2S*)-(+)-*cis*-2-hydroxy-1-indanyl]-1,2-benzisoselenazol-3(2*H*)-one **29a.**

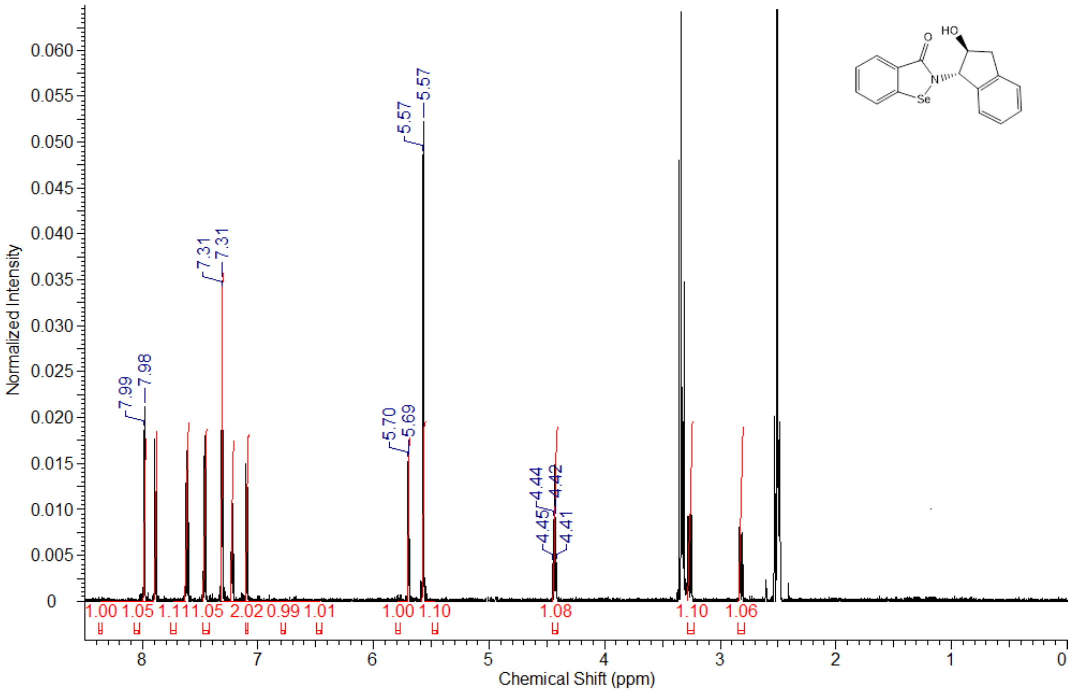

(a)

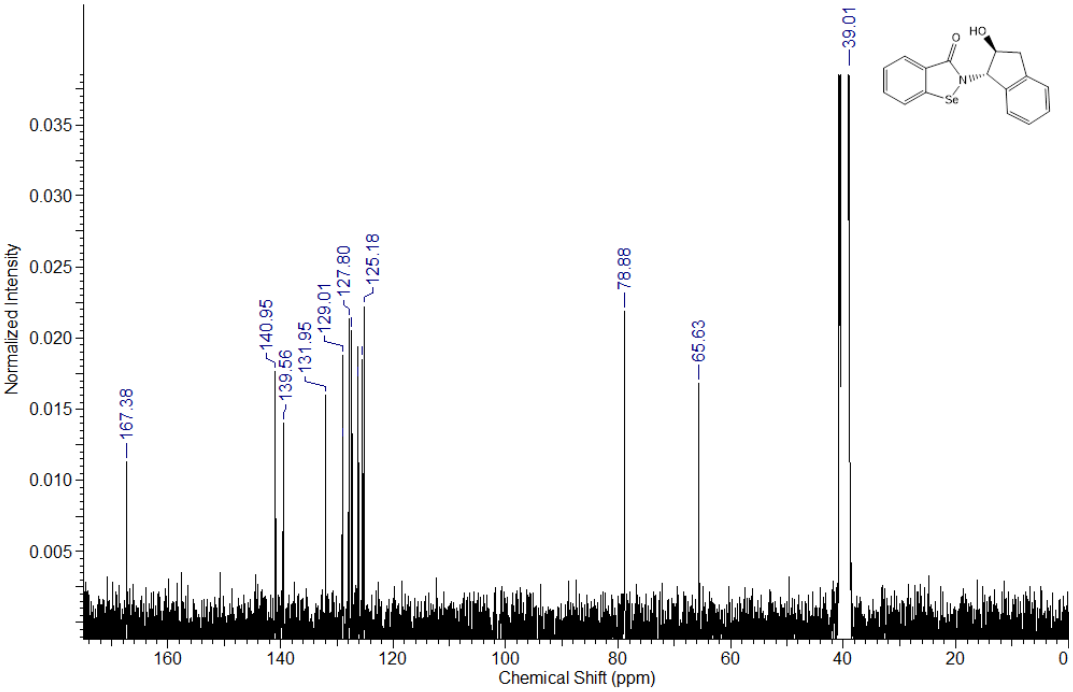

(b)

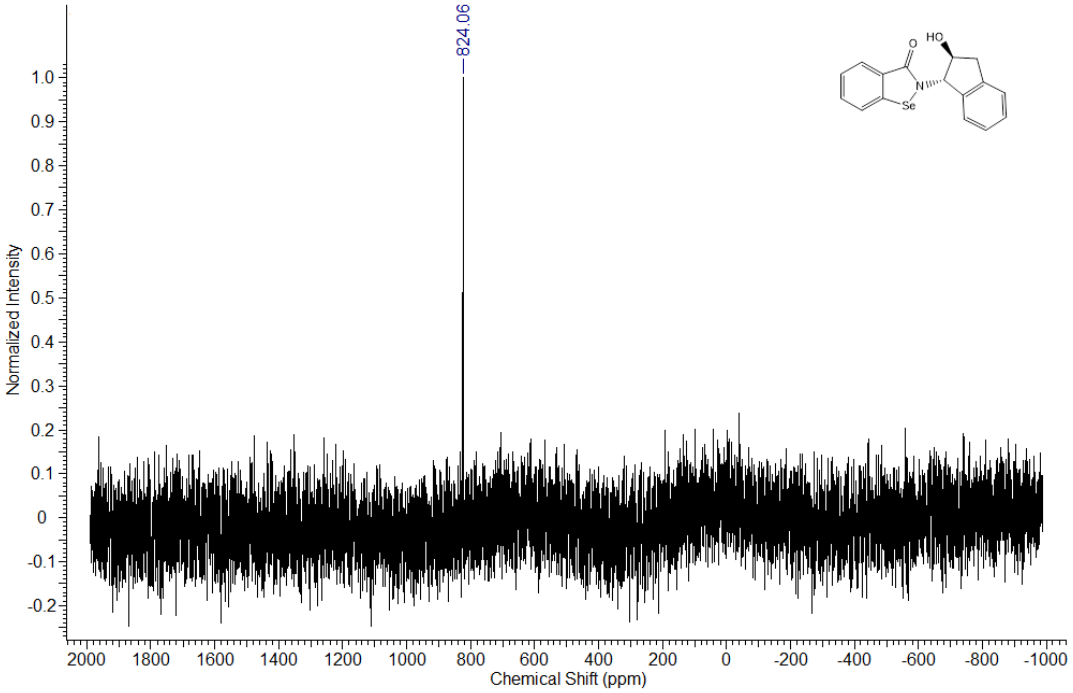

(c)

Figure S13. (**a**) 1H NMR, (**b**) 13C NMR, and (**c**) 77Se NMR spectra of *N*-[(*1S*,*2S*)-(+)-*trans*-2-hydroxy-1-indanyl]-1,2-benzisoselenazol-3(2*H*)-one **30a**

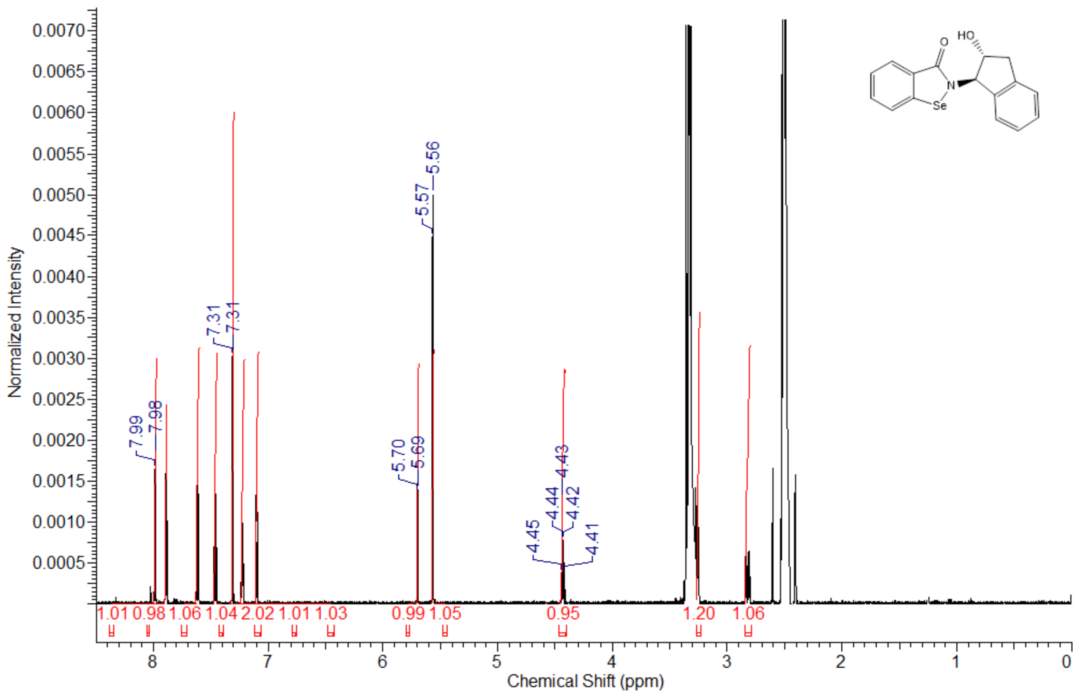
(a)

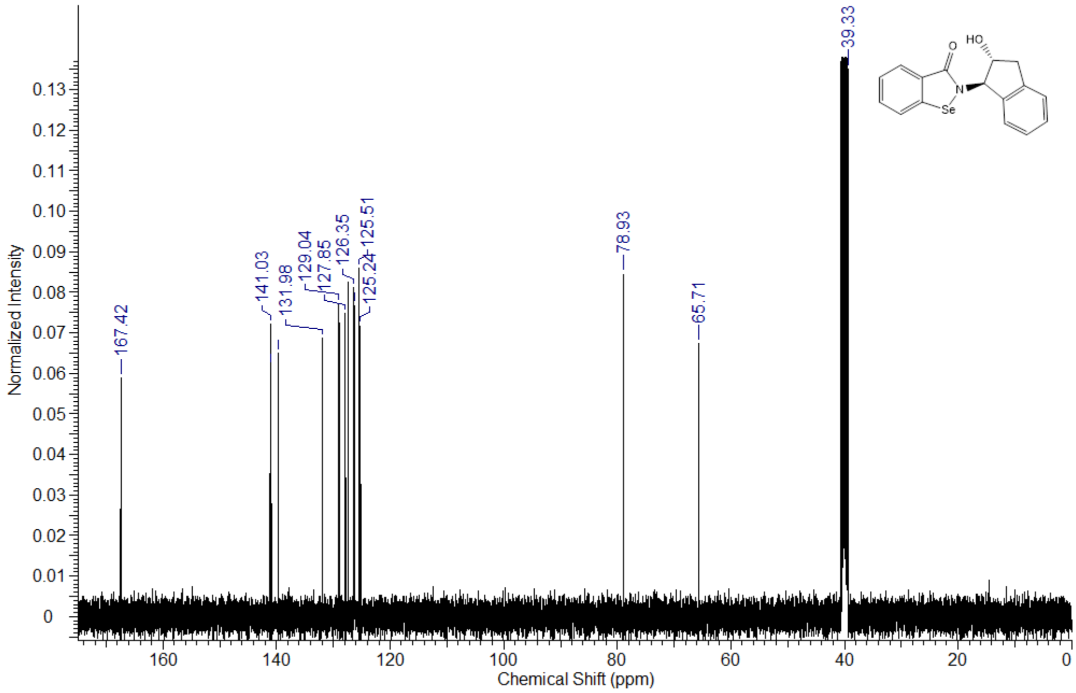
(b)

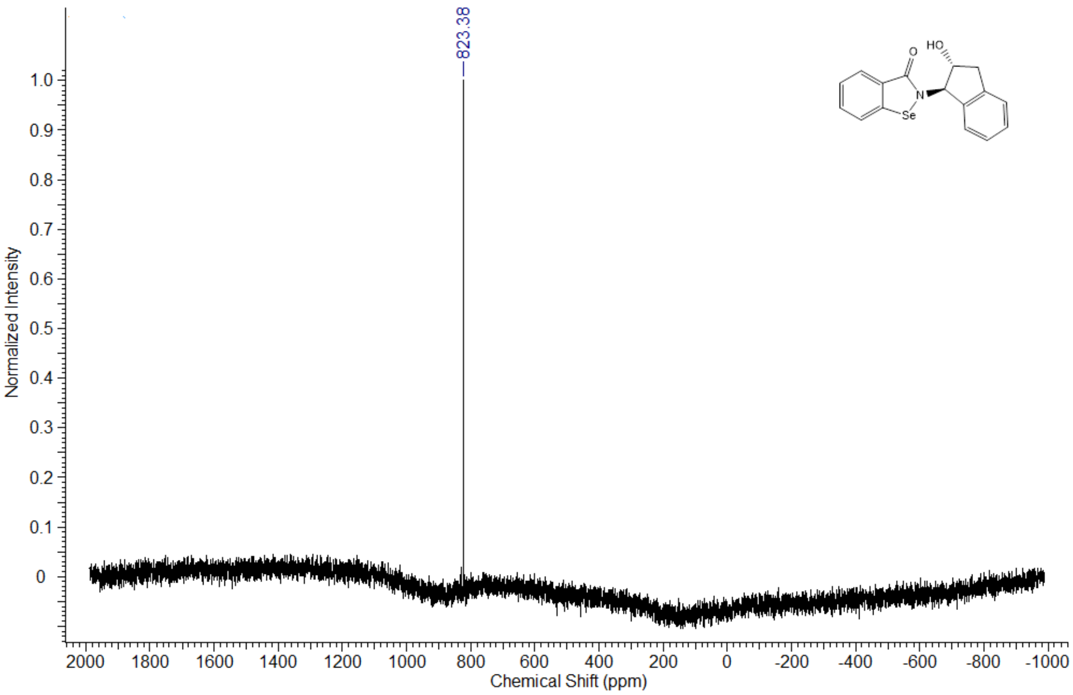

(c)

Figure S14. (**a**) 1H NMR, (**b**) 13C NMR, and (**c**) 77Se NMR spectra of *N*-[(*1R,2R*)-(-)-*trans*-2-hydroxy-1-indanyl]-1,2-benzisoselenazol-3(2*H*)-one **31a.**

2. NMR spectra of diselenides 18b-31b

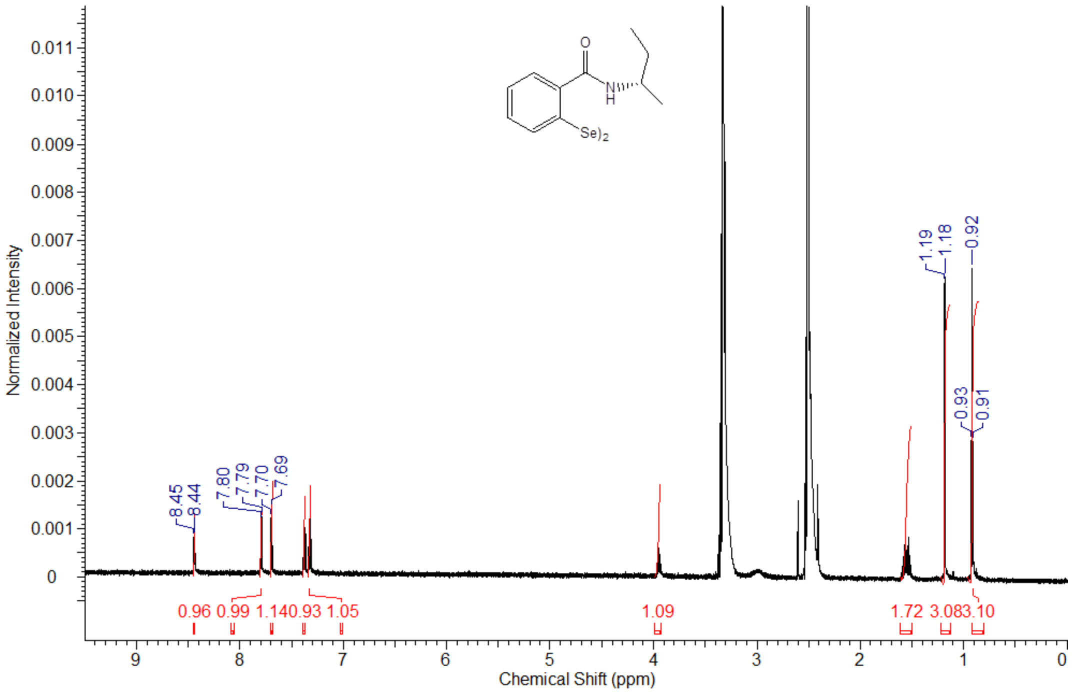

(a)

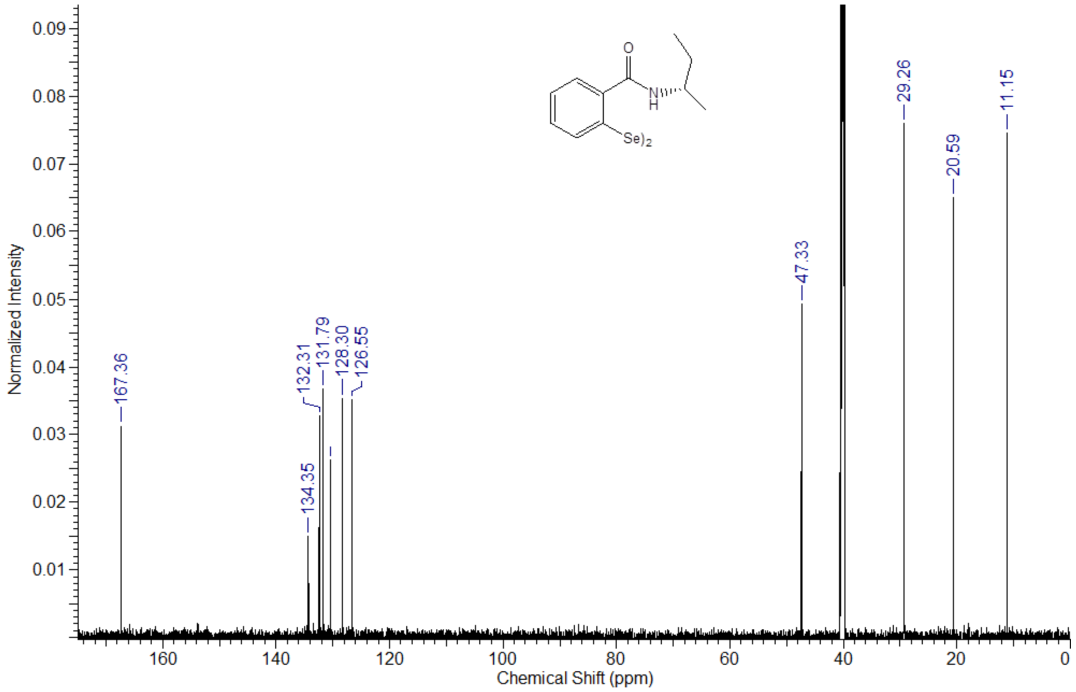

(b)

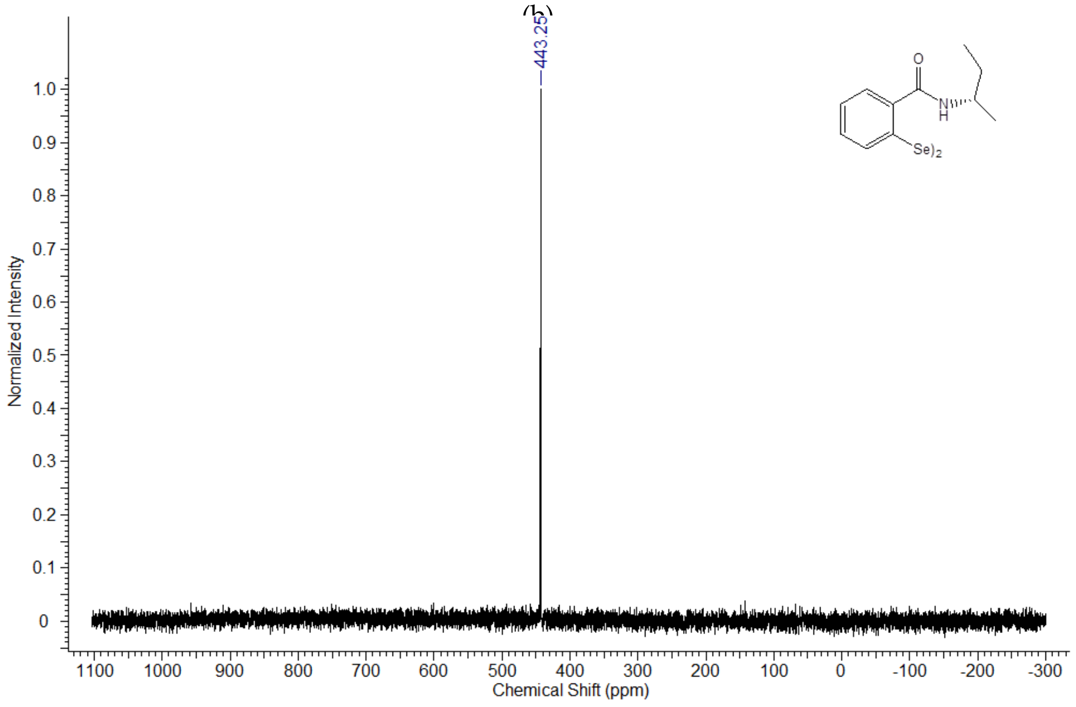

(c)

Figure S15. (**a**) 1H NMR, (**b**) 13C NMR, and (**c**) 77Se NMR spectra of 2,2′-diselenobis[*N*-(*S*)-(+)-*sec*-butylbezamide] **18b**

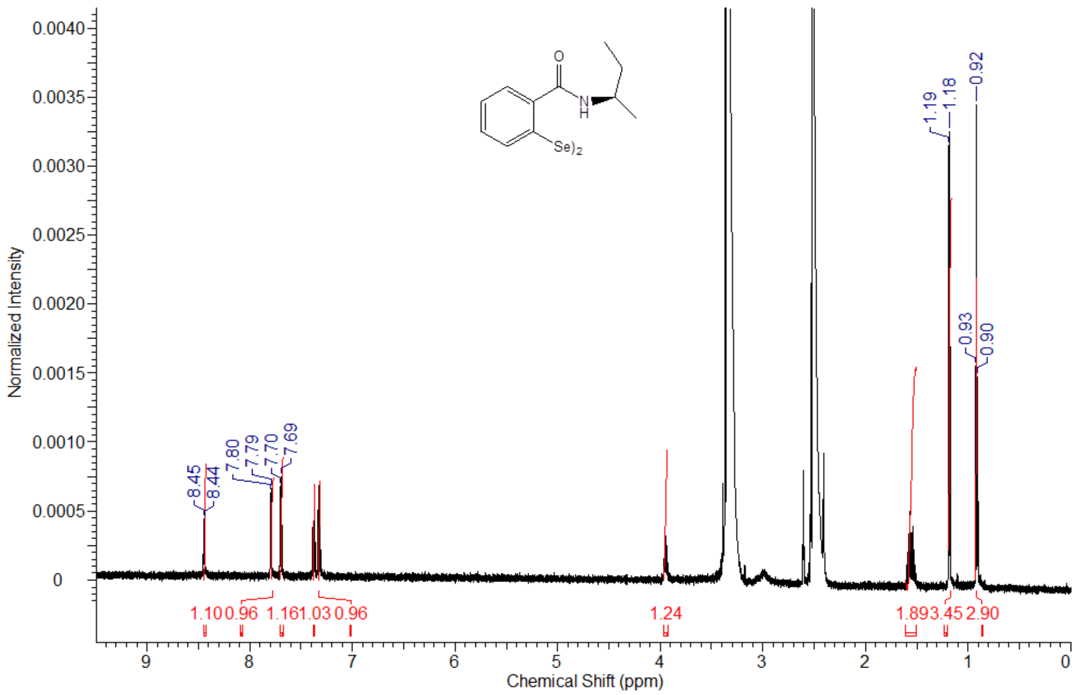

Normalized Intensity
Chemical Shift (ppm)
8.45
8.44
7.80
7.79
7.70
7.69
1.19
1.18
0.92
0.93
0.90
1.10
0.96
1.16
1.03
0.96
1.24
1.89
3.45
2.90
Se)2

(a)

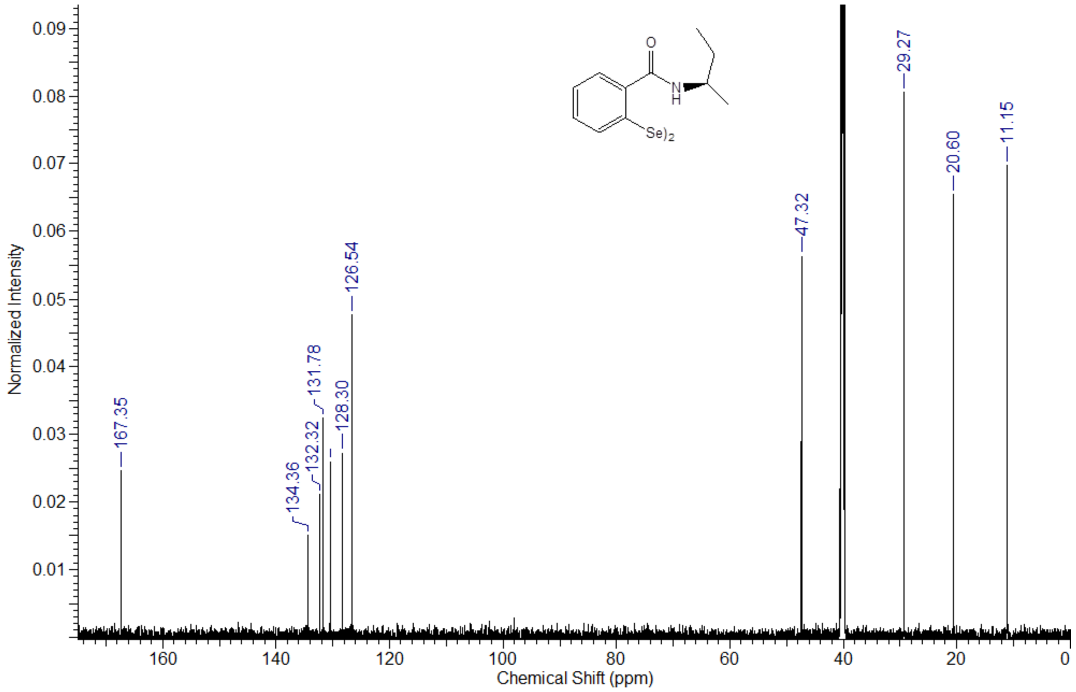

Normalized Intensity
Chemical Shift (ppm)
167.35
134.36
132.32
131.78
128.30
126.54
47.32
29.27
20.60
11.15
Se)2

(b)

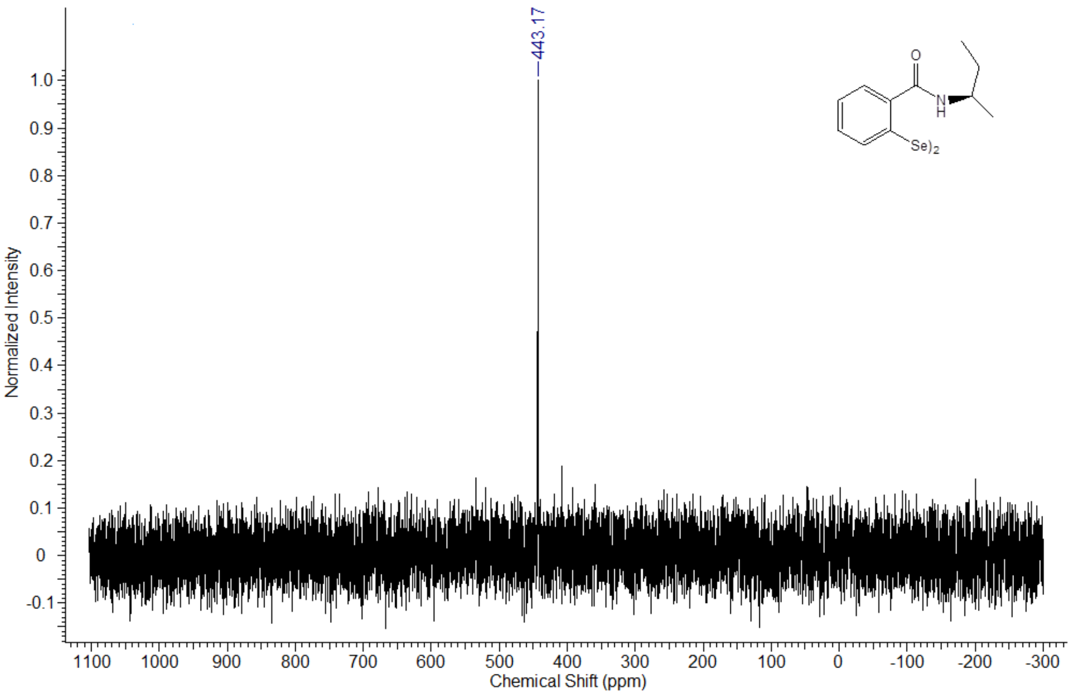

(c)

Figure S16. (**a**) 1H NMR, (**b**) 13C NMR, and (**c**) 77Se NMR spectra of 2,2′-diselenobis[*N*-(*R*)-(-)-*sec*-butylbezamide] **19b**

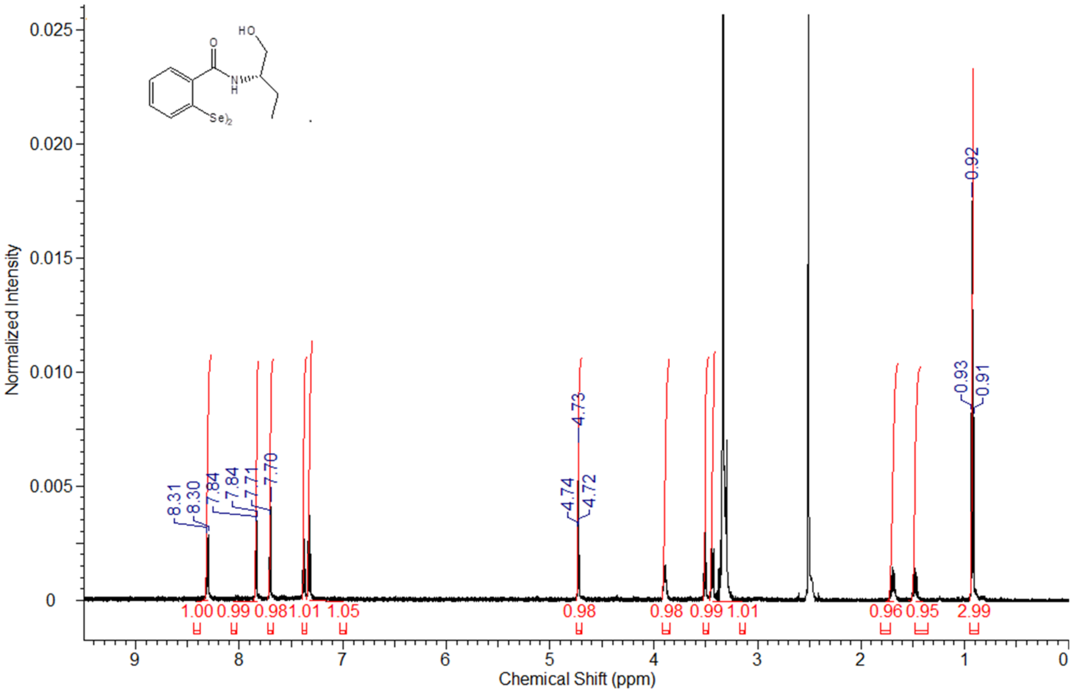

(a)

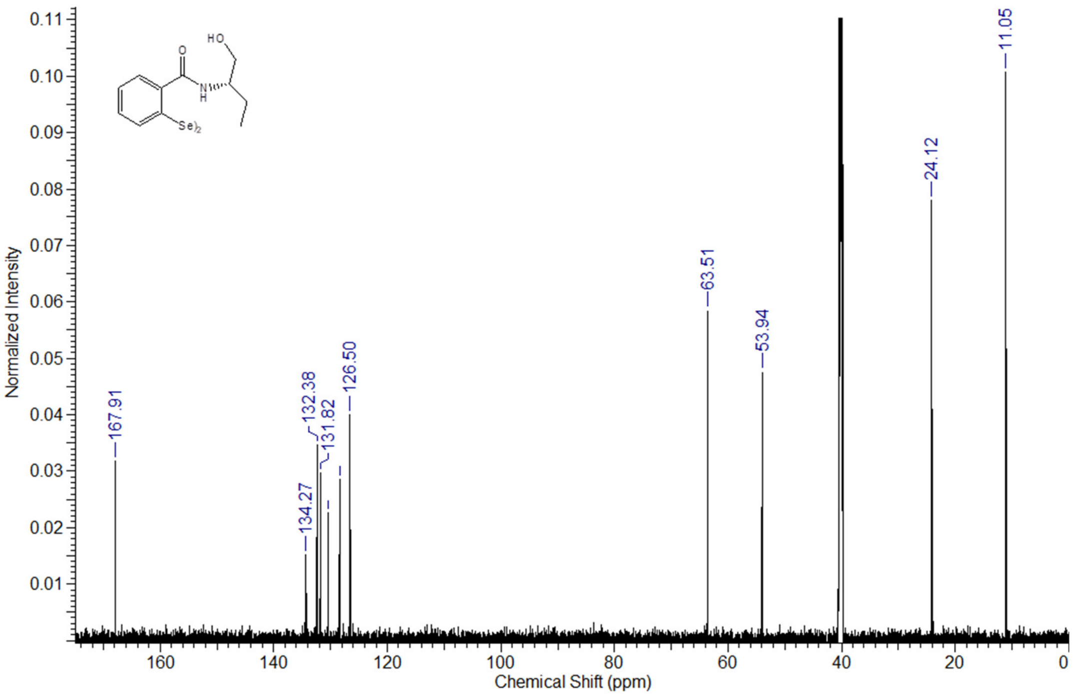

(b)

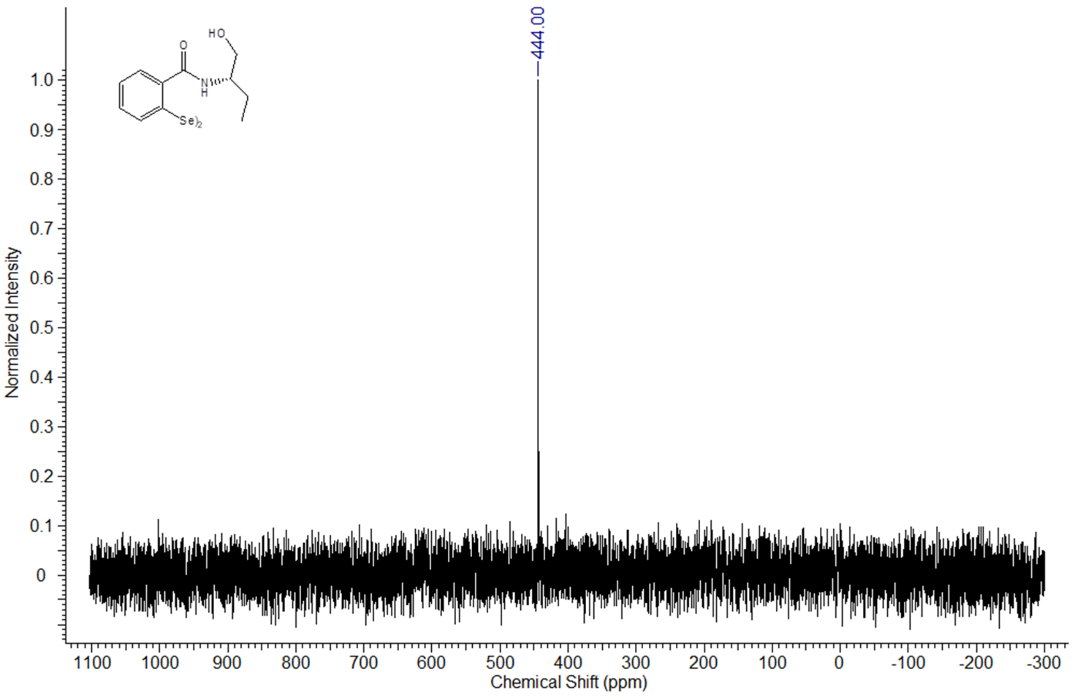

(c)

Figure S17. (**a**) 1H NMR, (**b**) 13C NMR, and (**c**) 77Se NMR spectra of 2,2′-diselenobis[*N*-(*S*)-(+)-1-hydroxy-2-butanylbezamide] **20b**

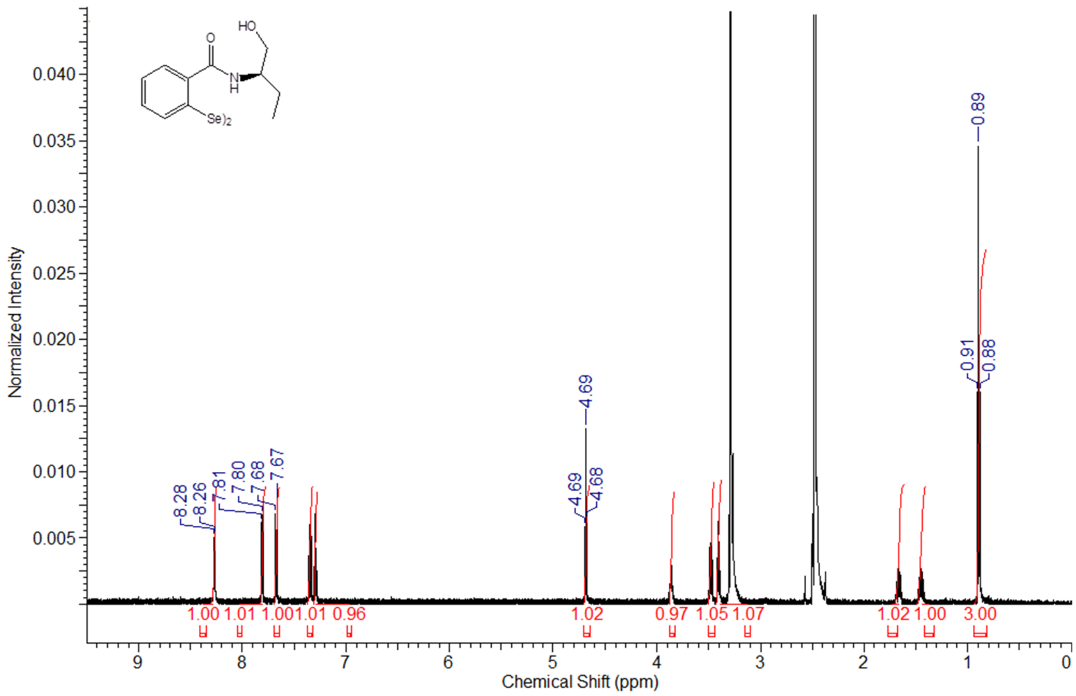
HO
O
N
H
Se)2
Normalized Intensity
0.040
0.035
0.030
0.025
0.020
0.015
0.010
0.005
8.28
8.26
7.81
7.80
7.68
7.67
4.69
4.69
4.68
0.91
0.89
0.88
1.00
1.01
1.00
1.01
0.96
1.02
0.97
1.05
1.07
1.02
1.00
3.00
9
8
7
6
5
4
3
2
1
0
Chemical Shift (ppm)

(a)

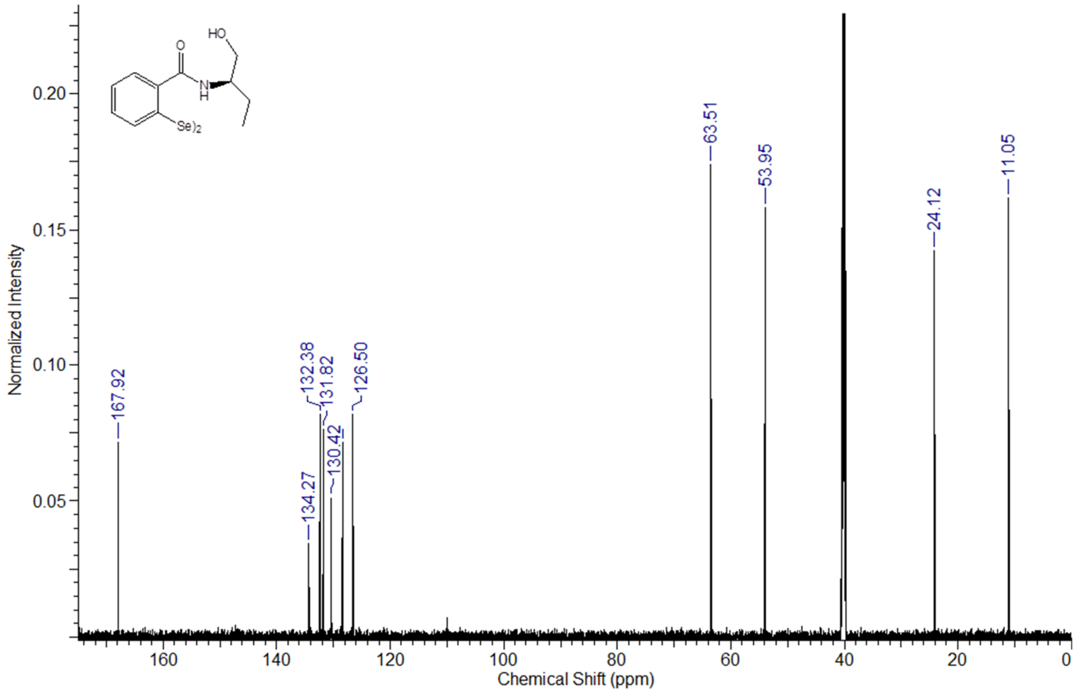
HO
O
N
H
Se)2
Normalized Intensity
0.20
0.15
0.10
0.05
167.92
134.27
132.38
131.82
130.42
126.50
63.51
53.95
24.12
11.05
160
140
120
100
80
60
40
20
0
Chemical Shift (ppm)

(b)

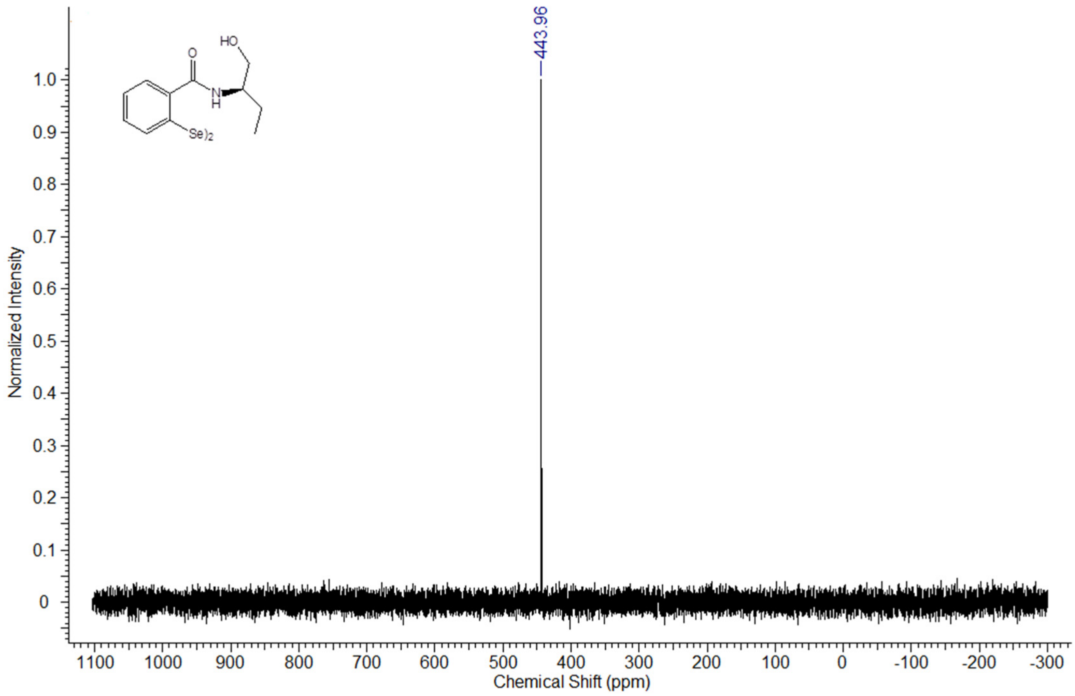

(c)

Figure S18. (**a**) 1H NMR, (**b**) 13C NMR, and (**c**) 77Se NMR spectra of 2,2′-diselenobis[*N*-(*R*)-(-)-1-hydroxy-2-butanylbezamide] **21b.**

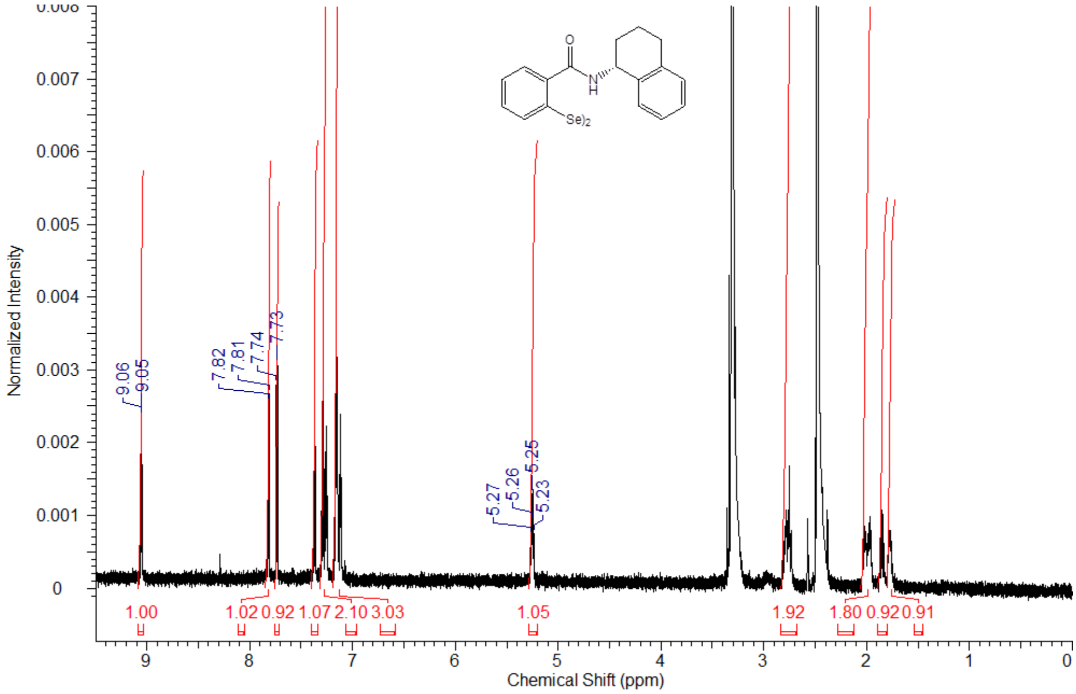

(a)

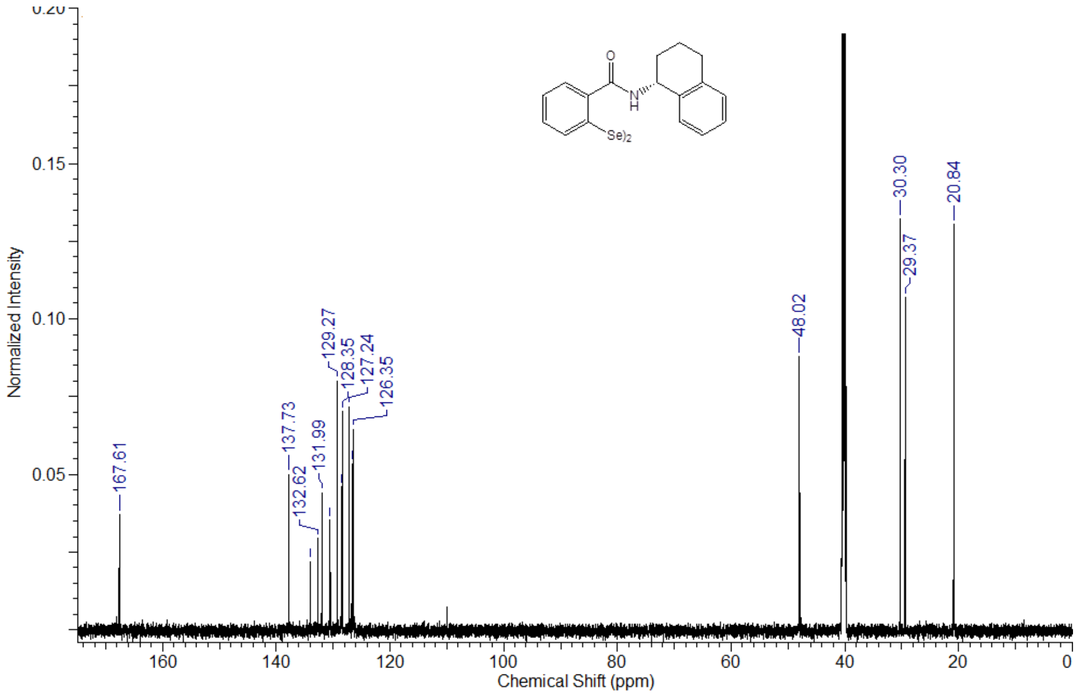

(b)

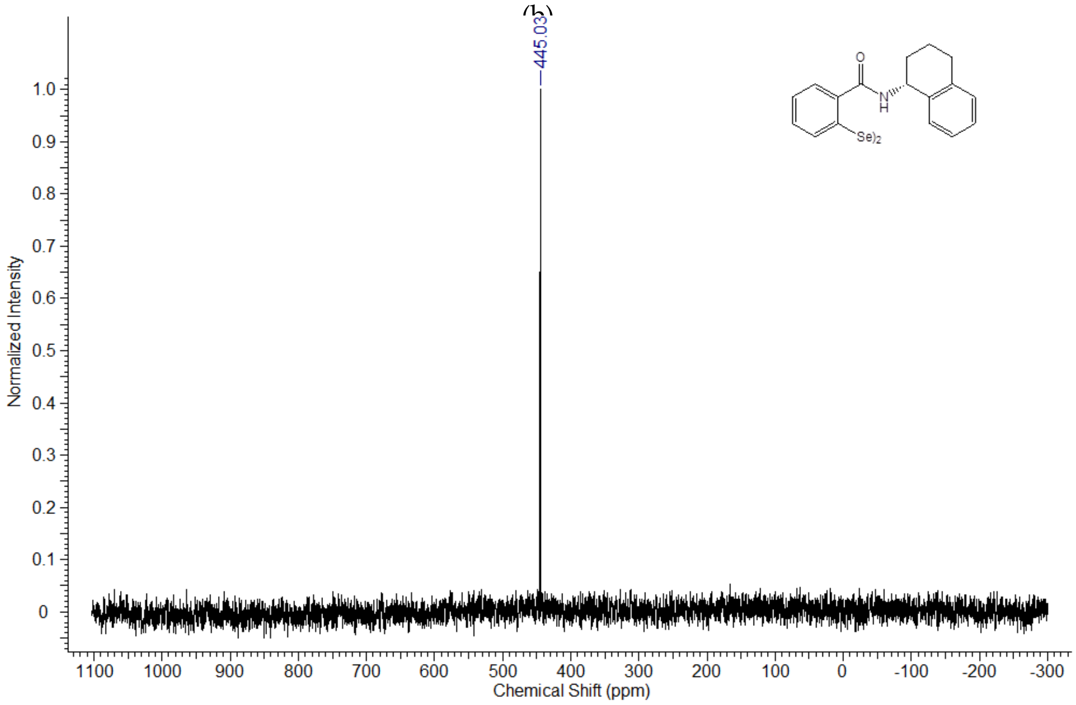

(c)

Figure S19. (**a**) 1H NMR, (**b**) 13C NMR, and (**c**) 77Se NMR spectra of 2,2′-diselenobis[*N*-(*R*)-(-)-1,2,3,4-tetrahydro-1-napthylbezamide] **22b**

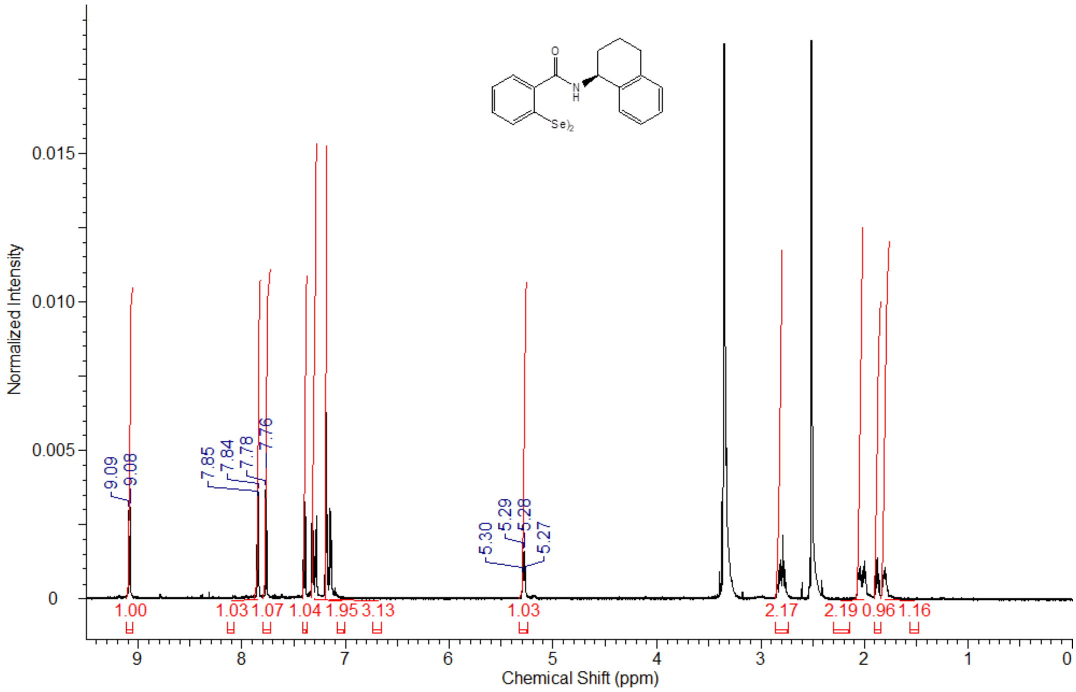

Normalized Intensity
0.015
0.010
0.005
0
9.09
9.08
7.85
7.84
7.78
7.76
5.30
5.29
5.28
5.27
1.00
1.03
1.07
1.04
1.95
3.13
1.03
2.17
2.19
0.96
1.16
Se)2
O
N
H
Chemical Shift (ppm)

(a)

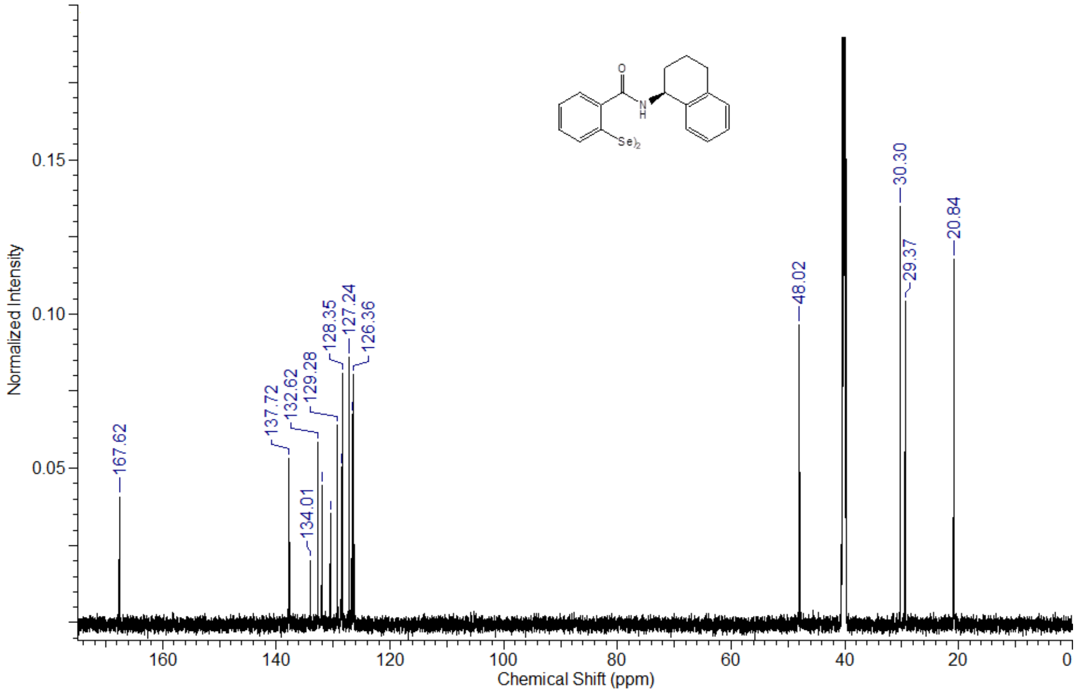

Normalized Intensity
0.15
0.10
0.05
167.62
137.72
132.62
129.28
128.35
127.24
126.36
134.01
48.02
30.30
29.37
20.84
Se)2
O
N
H
Chemical Shift (ppm)

(b)

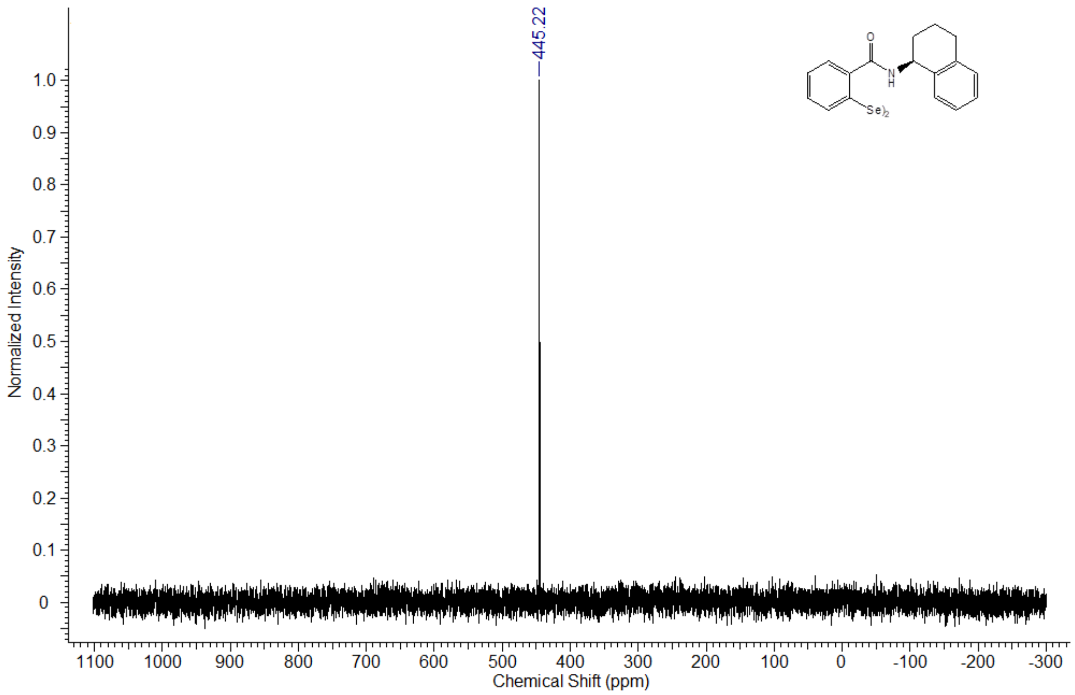

(c)

Figure S20. (**a**) 1H NMR, (**b**) 13C NMR, and (**c**) 77Se NMR spectra of 2,2′-diselenobis[*N*-(*S*)-(+)-1,2,3,4-tetrahydro-1-napthylbezamide] **23b**

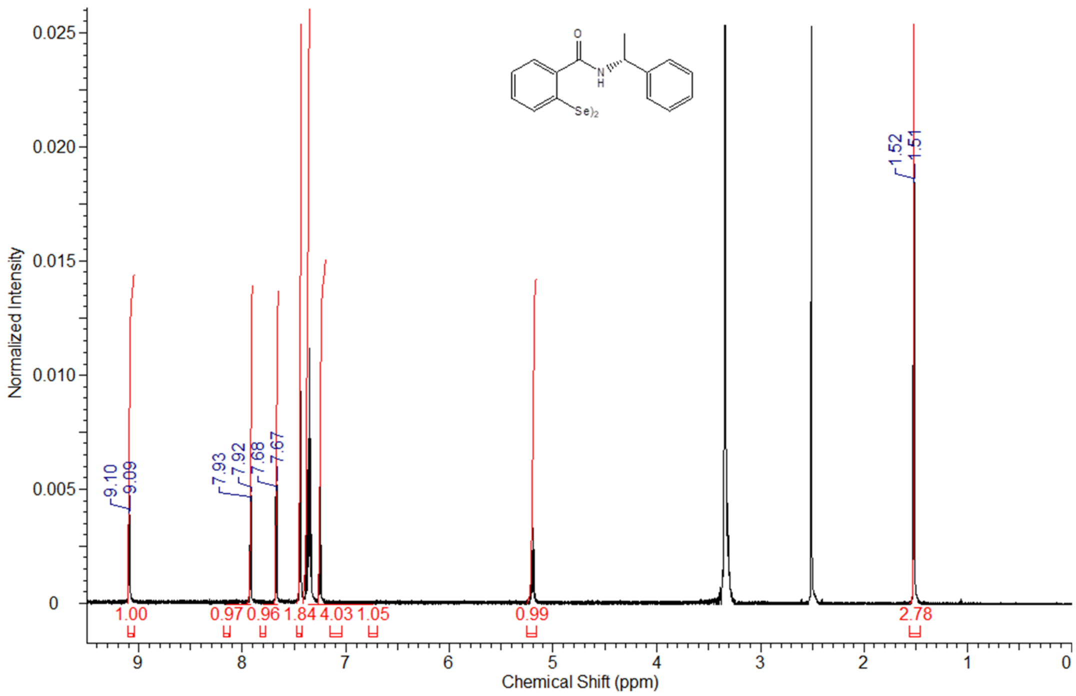

(a)

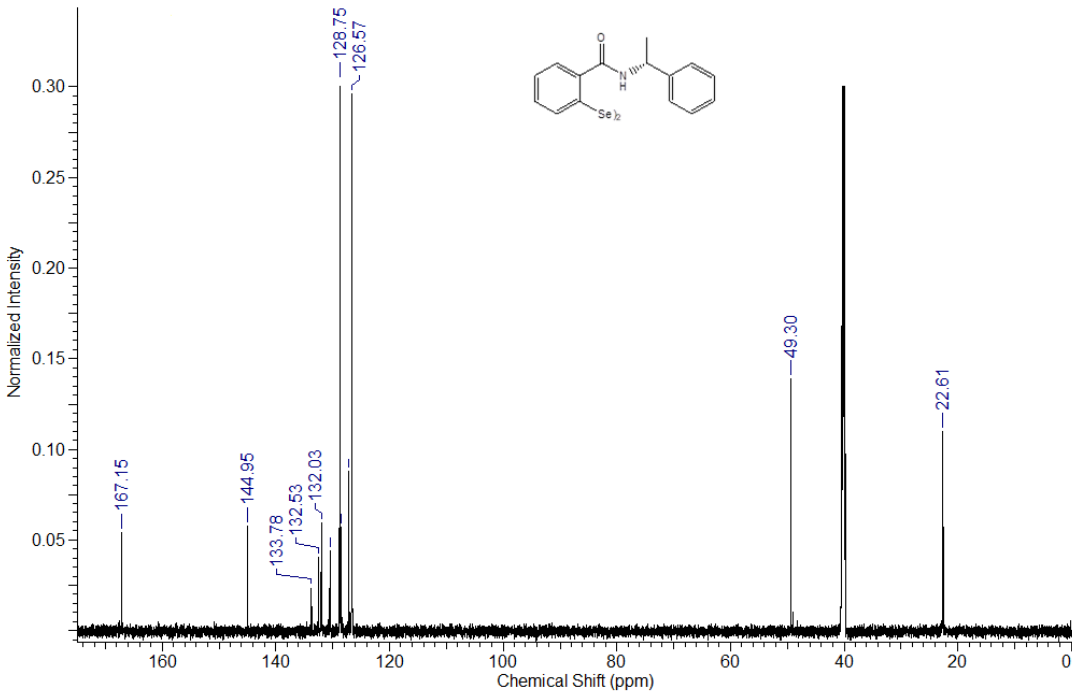

(b)

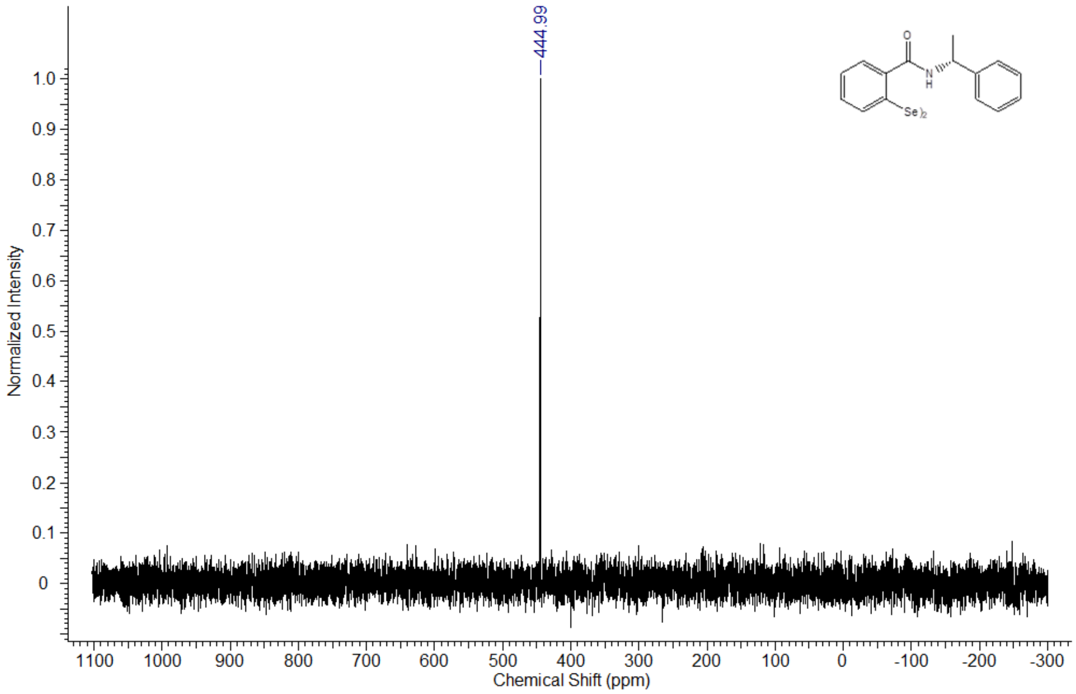

(c)

Figure S21. (**a**) 1H NMR, (**b**) 13C NMR, and (**c**) 77Se NMR spectra of 2,2′-diselenobis[*N*-(*R*)-(+)-*α*-methylbenzylbezamide] **24b.**

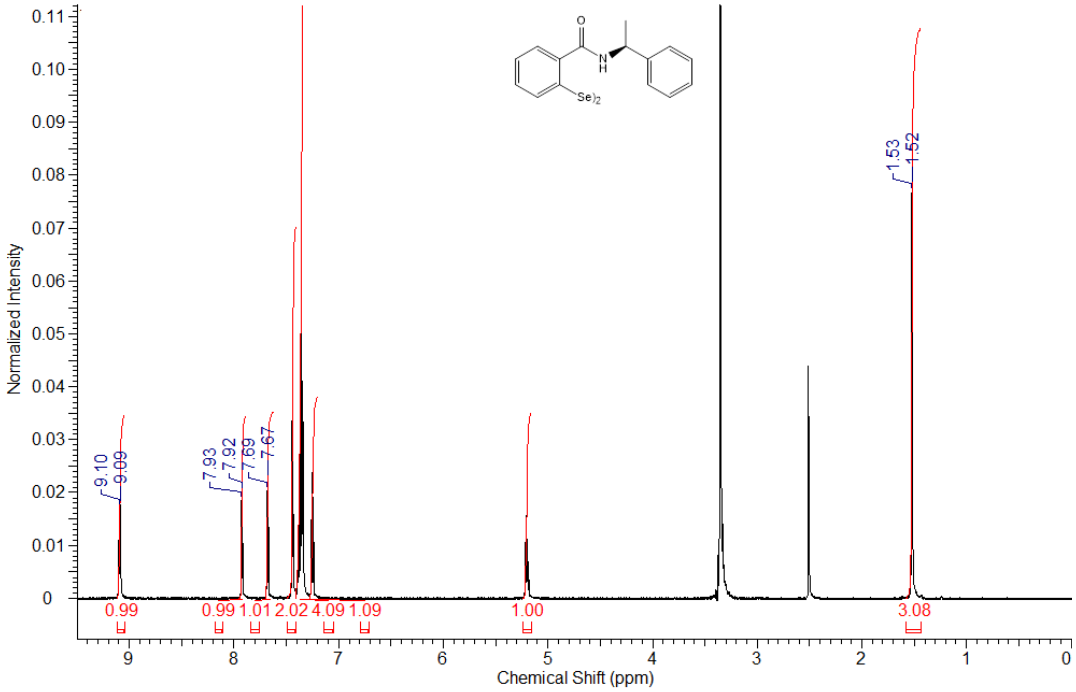
Se)2
0.11
0.10
0.09
0.08
0.07
0.06
0.05
0.04
0.03
0.02
0.01
0
Normalized Intensity
9.10
9.09
7.93
7.92
7.69
7.67
1.53
1.52
0.99
0.99
1.01
2.02
4.09
1.09
1.00
3.08
9
8
7
6
5
4
3
2
1
0
Chemical Shift (ppm)

(a)

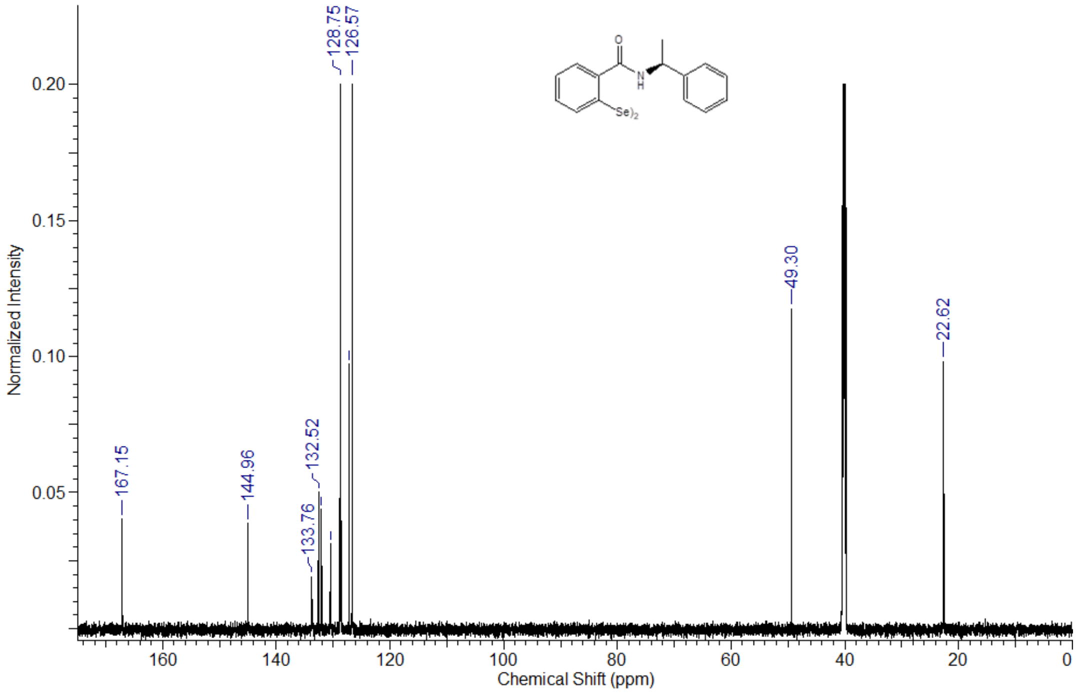
Se)2
0.20
0.15
0.10
0.05
Normalized Intensity
167.15
144.96
133.76
132.52
128.75
126.57
49.30
22.62
160
140
120
100
80
60
40
20
0
Chemical Shift (ppm)

(b)

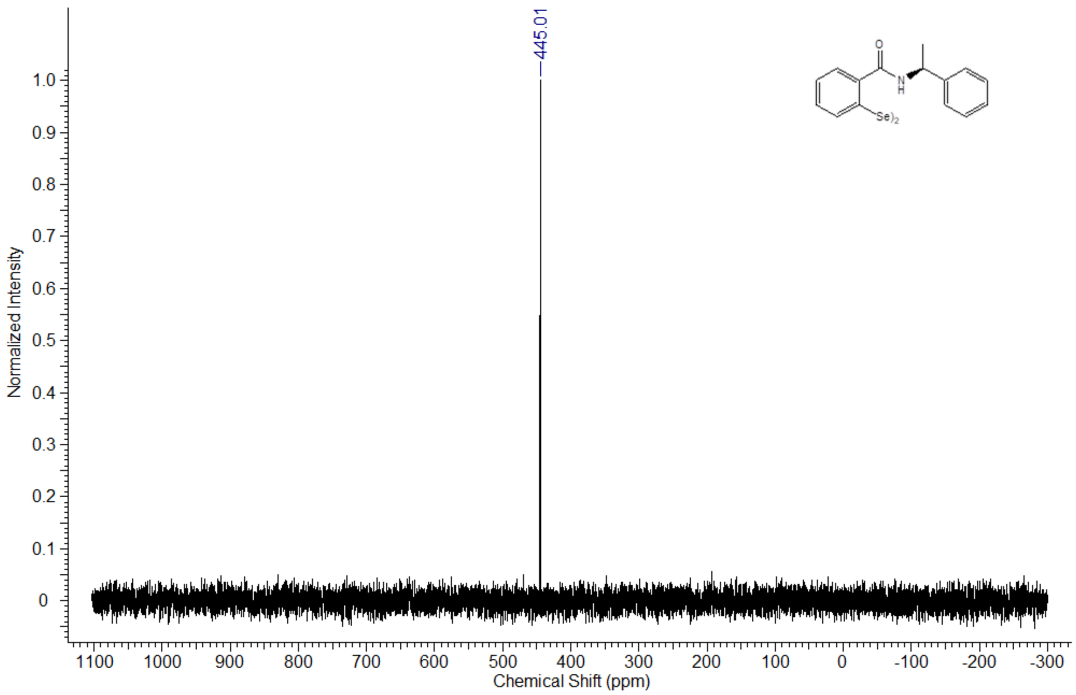

(c)

Figure S22. (**a**) 1H NMR, (**b**) 13C NMR, and (**c**) 77Se NMR spectra of 2,2′-diselenobis[*N*-(*S*)-(-)-*α*-methylbenzylbezamide] **25b.**

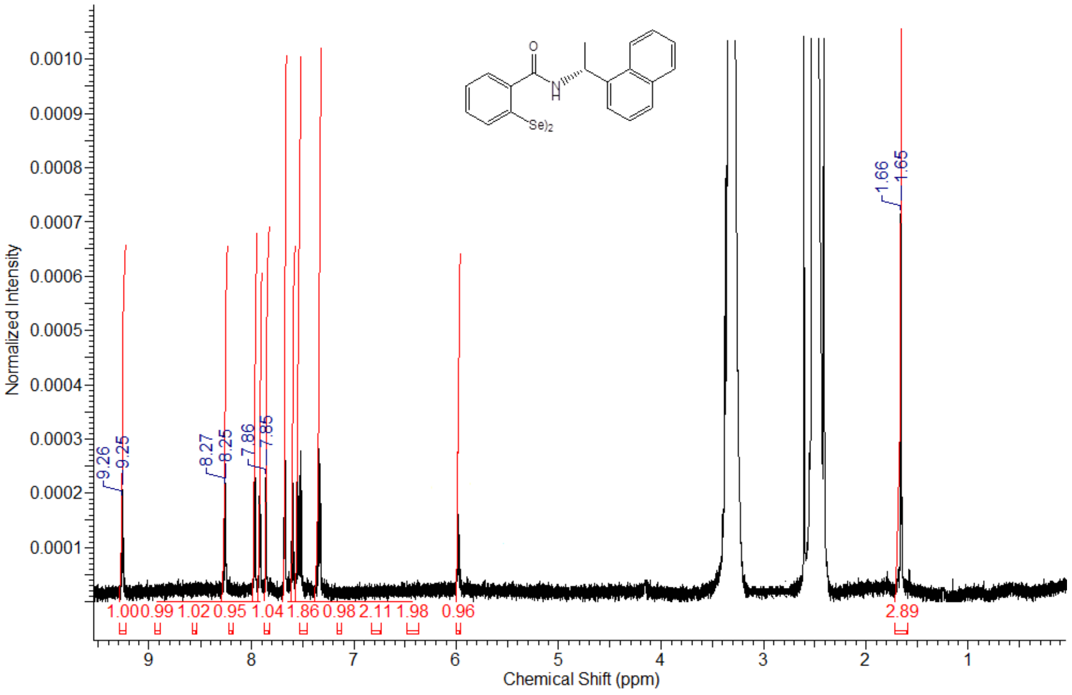

(a)

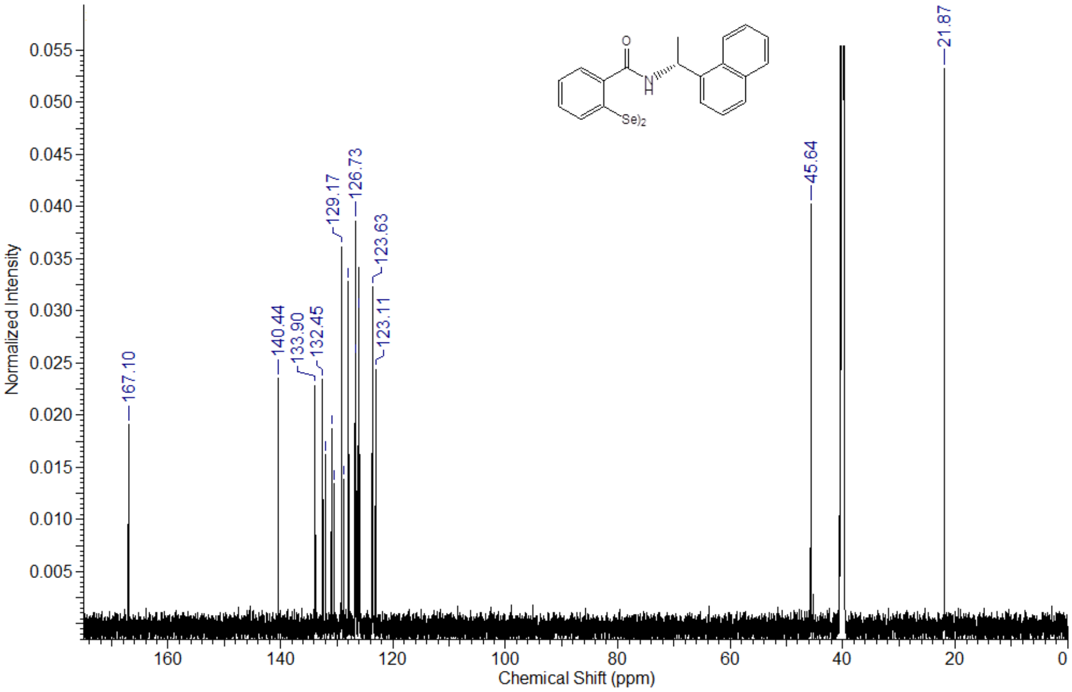

(b)

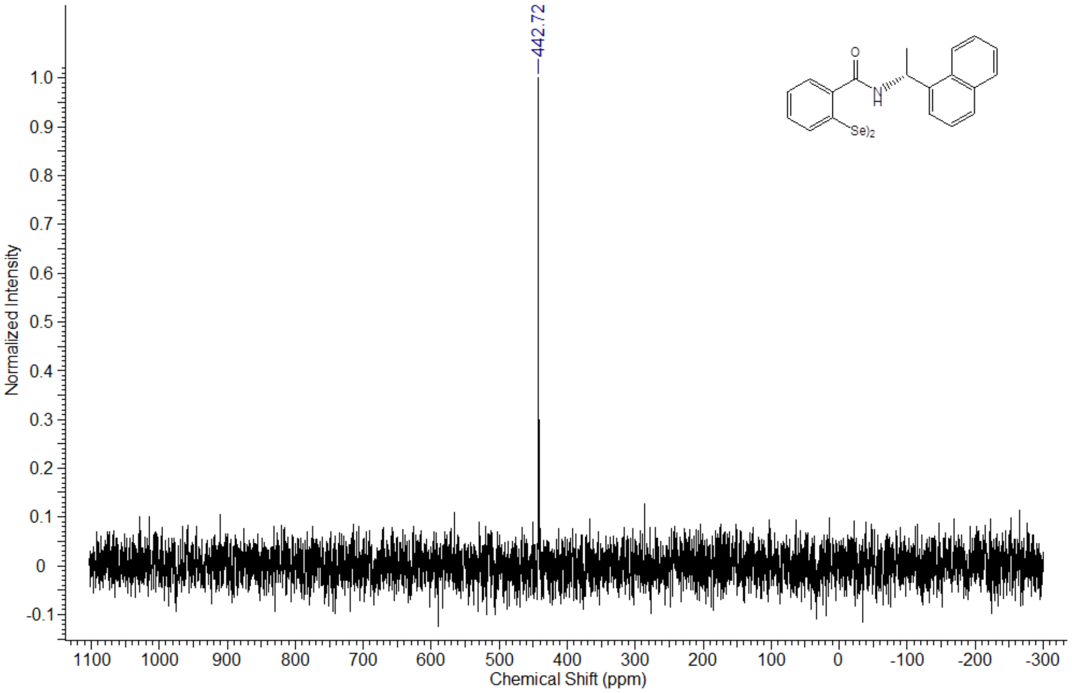

(c)

Figure S23. (**a**) 1H NMR, (**b**) 13C NMR, and (**c**) 77Se NMR spectra of 2,2′-diselenobis[*N*-(*S*)-(-)-1-(1-napthyl)ethylbezamide] **26b.**

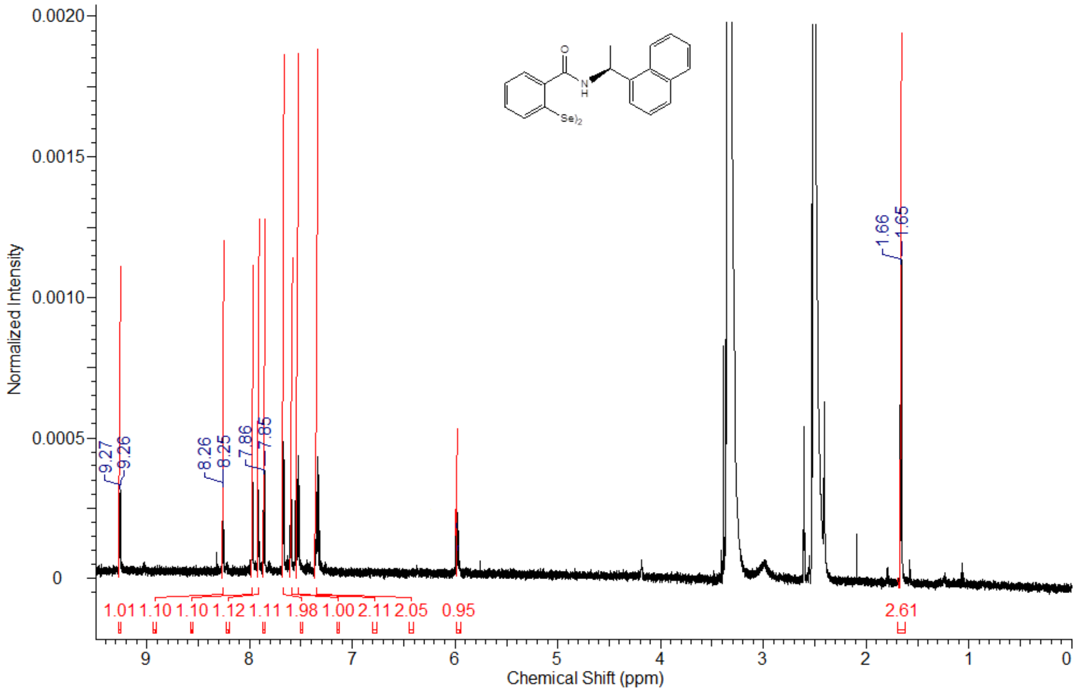
Normalized Intensity
Chemical Shift (ppm)
9.27
9.26
8.26
8.25
7.86
7.85
1.66
1.65
1.01 1.10 1.10 1.12 1.11 1.98 1.00 2.11 2.05 0.95
2.61

(a)

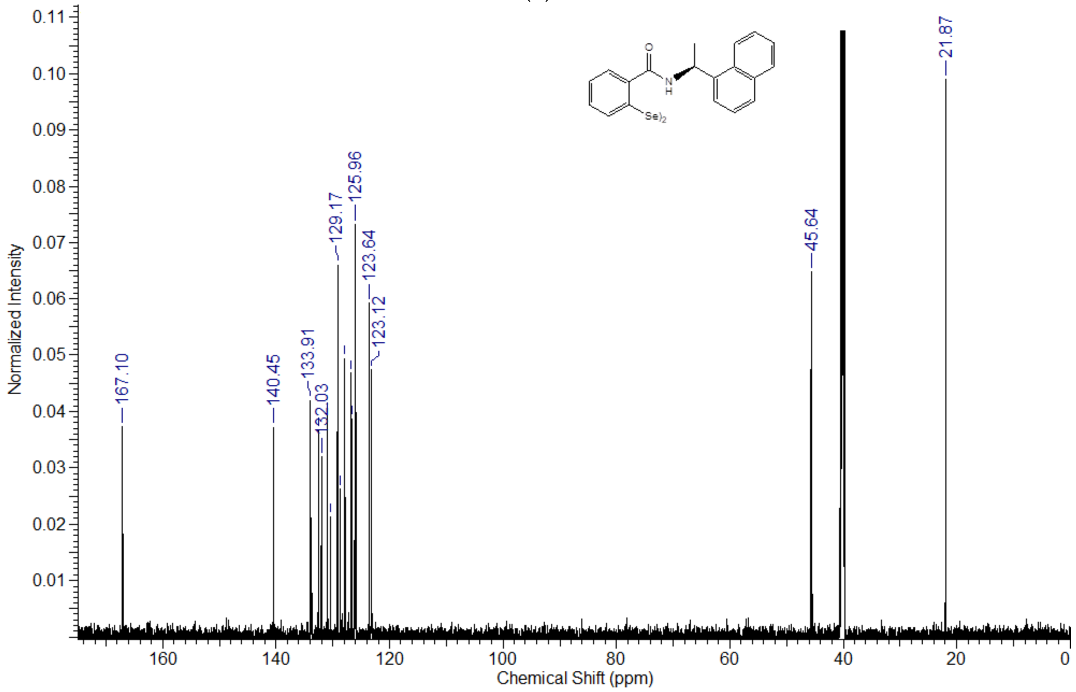
Normalized Intensity
Chemical Shift (ppm)
167.10
140.45
133.91
132.03
129.17
125.96
123.64
123.12
45.64
21.87

(b)

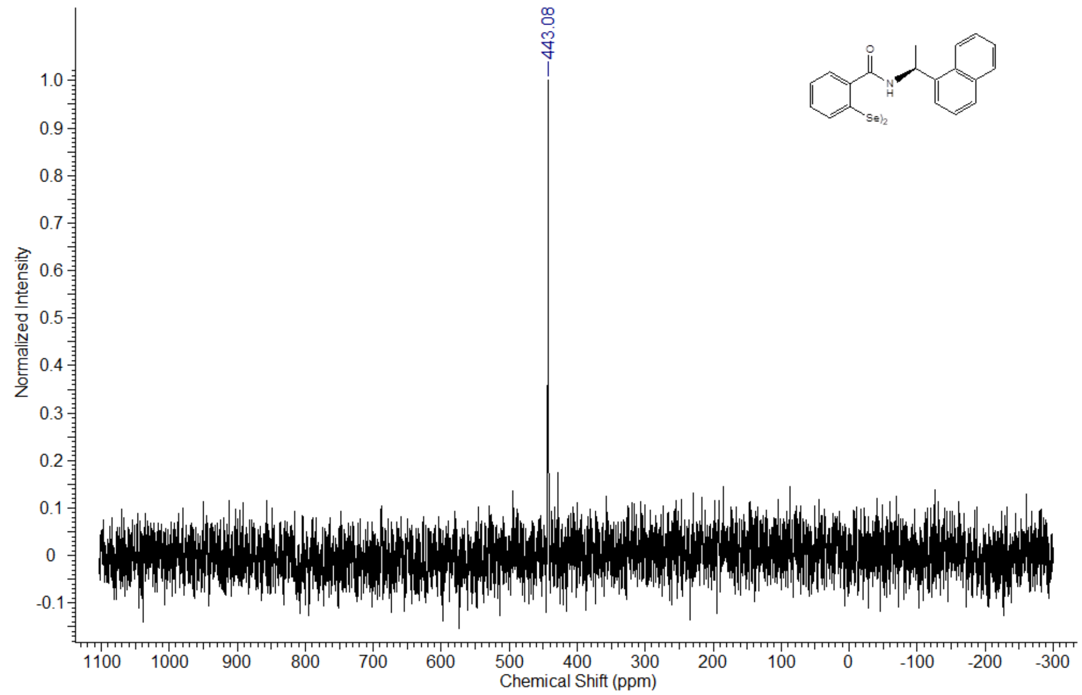

(c)

Figure S24. (**a**) 1H NMR, (**b**) 13C NMR, and (**c**) 77Se NMR spectra of 2,2′-diselenobis[*N*-(*R*)-(+)-1-(1-napthyl)ethylbezamide] **27b.**

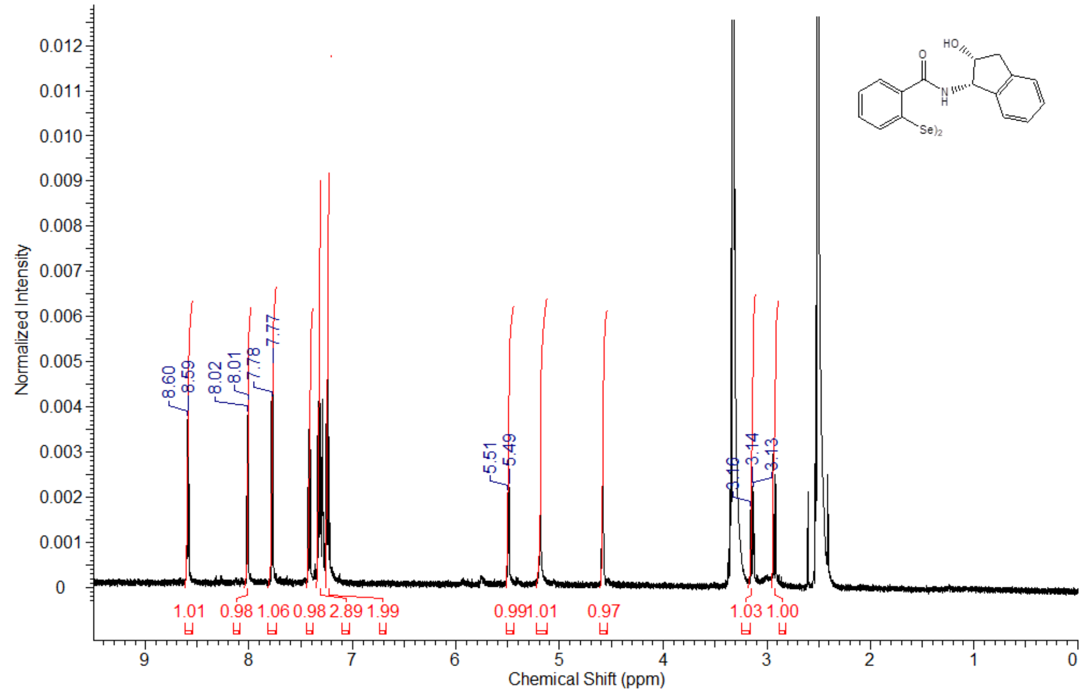

(a)

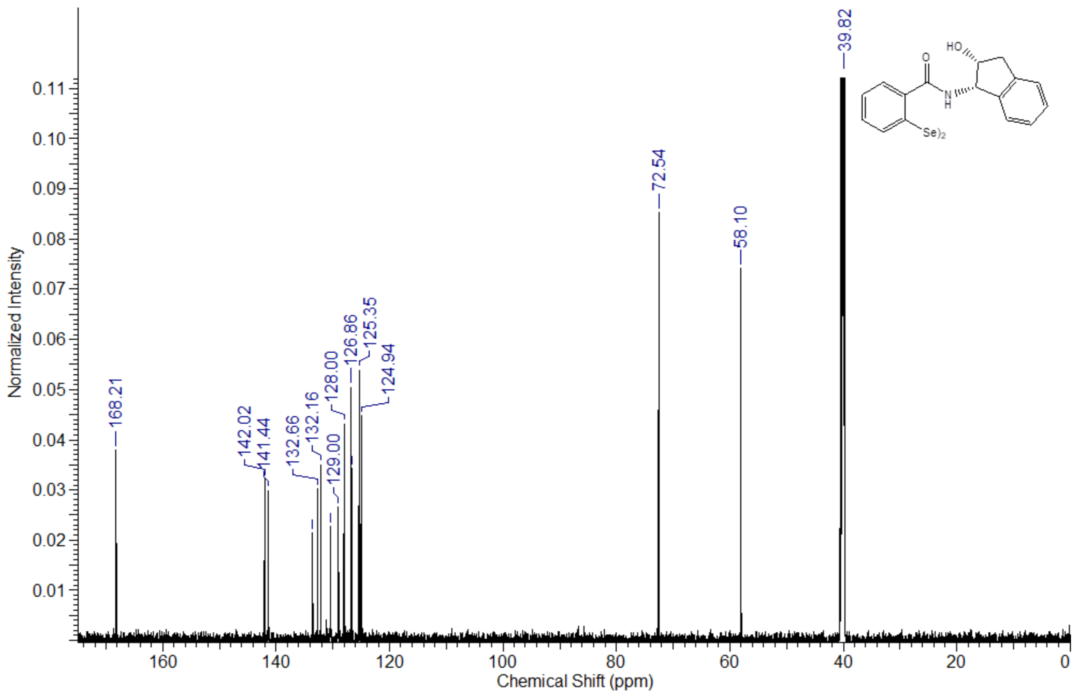

(b)

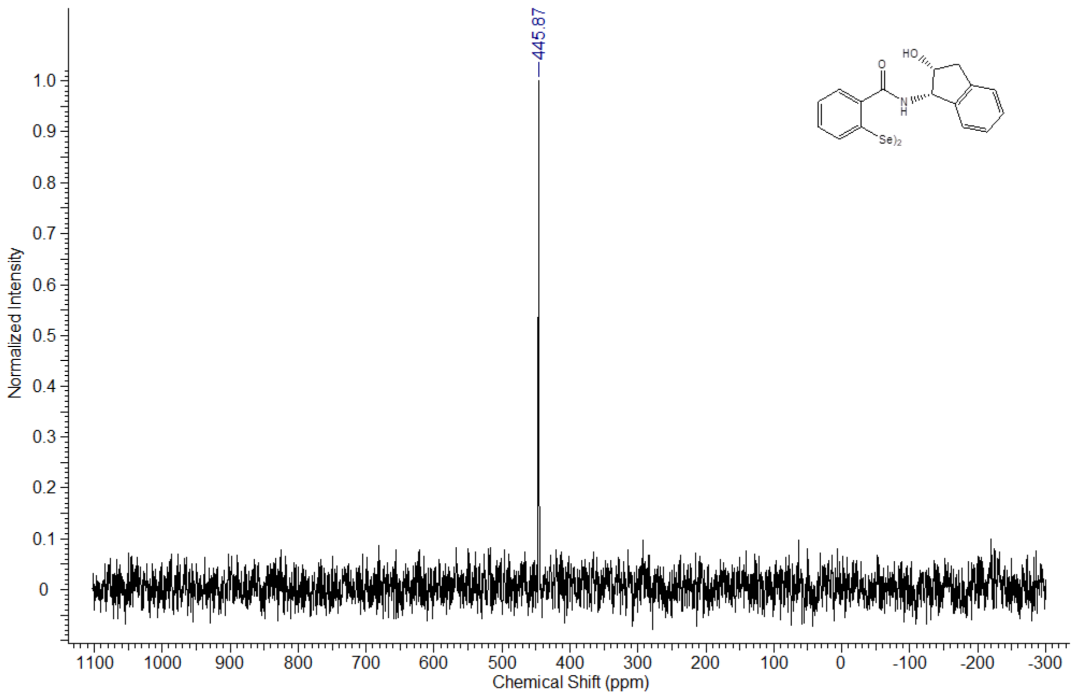

(c)

Figure S25. (**a**) 1H NMR, (**b**) 13C NMR, and (**c**) 77Se NMR spectra of 2,2′-diselenobis[*N*-(1*S*,2*R*)-(-)-*cis*-2-hydroxy-1-indanylbezamide] **28b.**

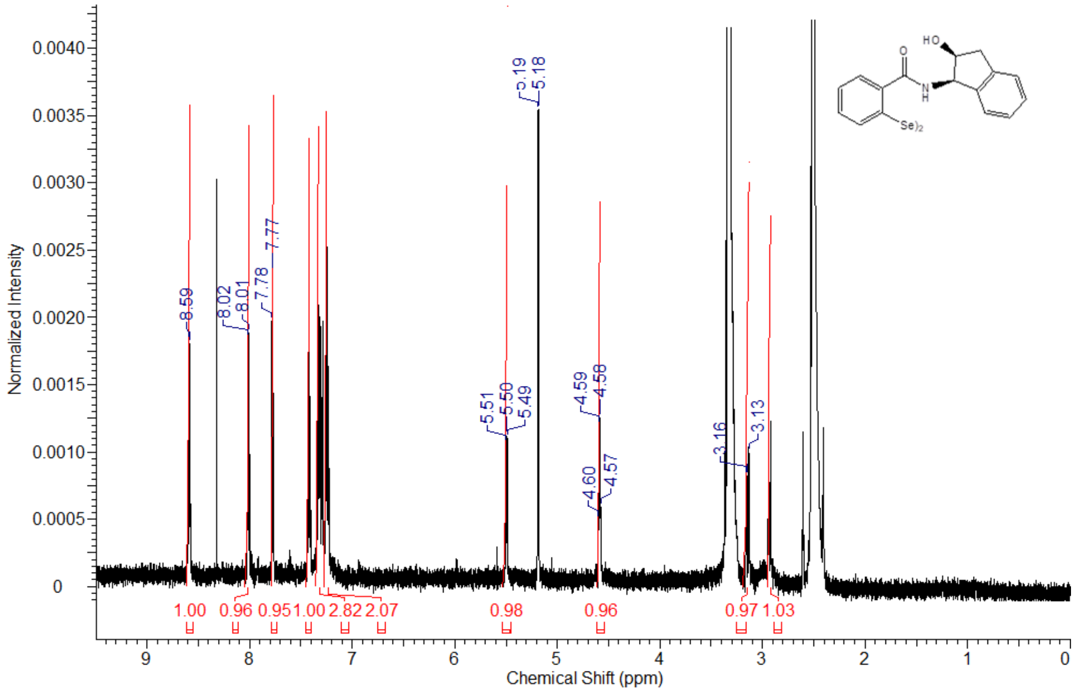
Normalized Intensity
Chemical Shift (ppm)
HO
O
N
H
Se)2

(a)

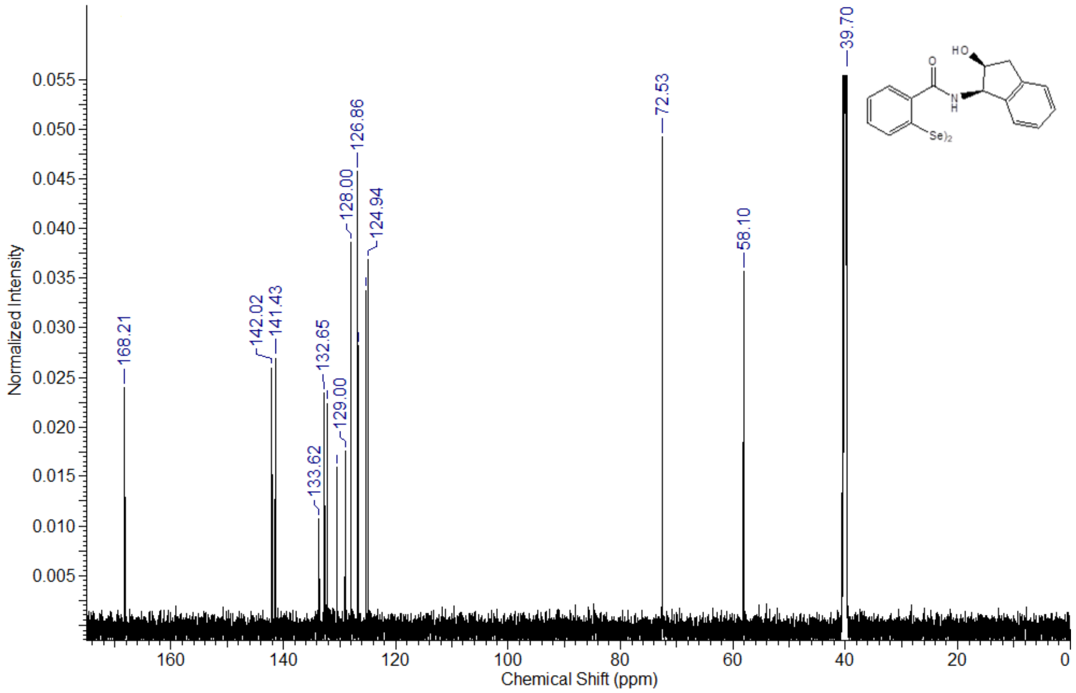
Normalized Intensity
Chemical Shift (ppm)
HO
O
N
H
Se)2

(b)

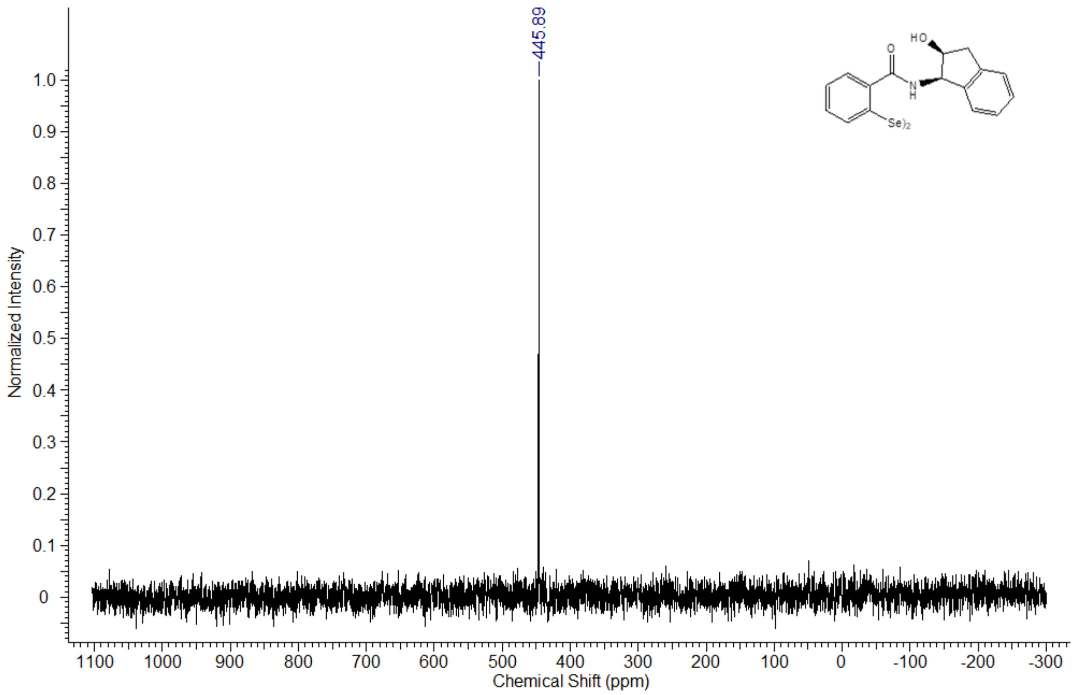

(c)

Figure S26. (**a**) 1H NMR, (**b**) 13C NMR, and (**c**) 77Se NMR spectra of 2,2′-diselenobis[*N*-(*1R,2S*)-(+)-*cis*-2-hydroxy-1-indanylbezamide] **29b.**

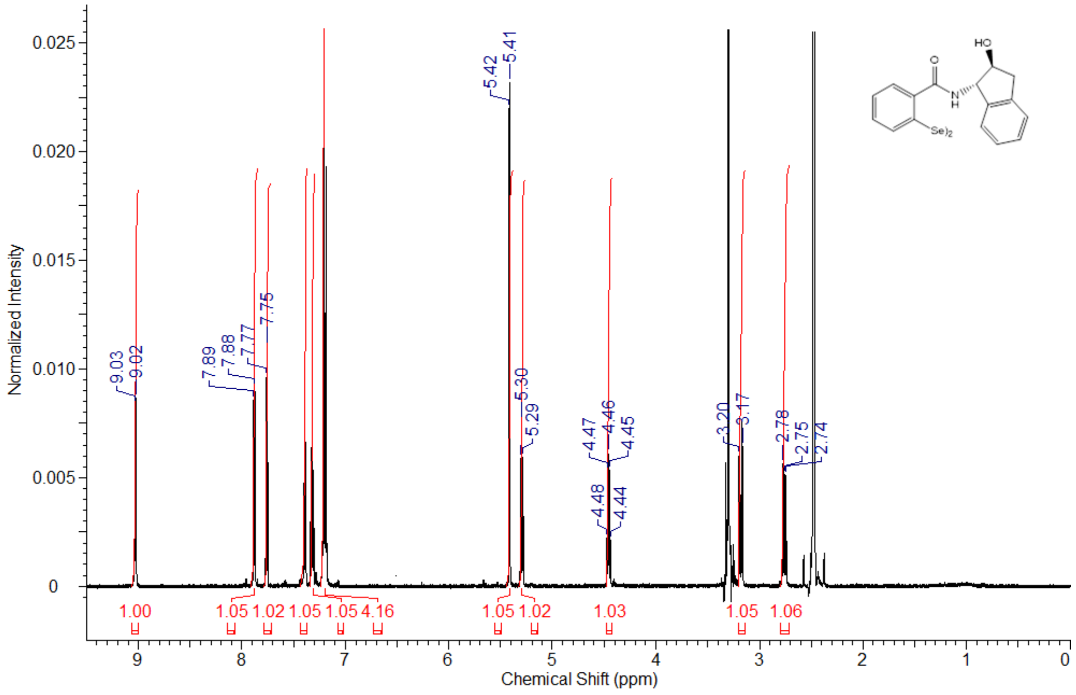

(a)

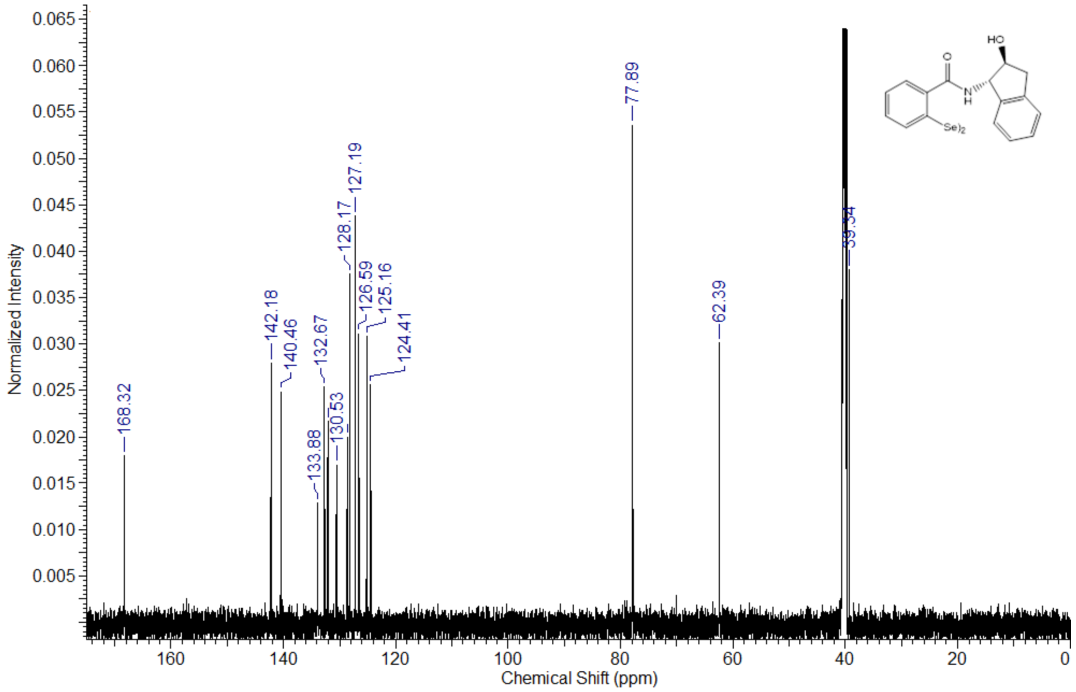

(b)

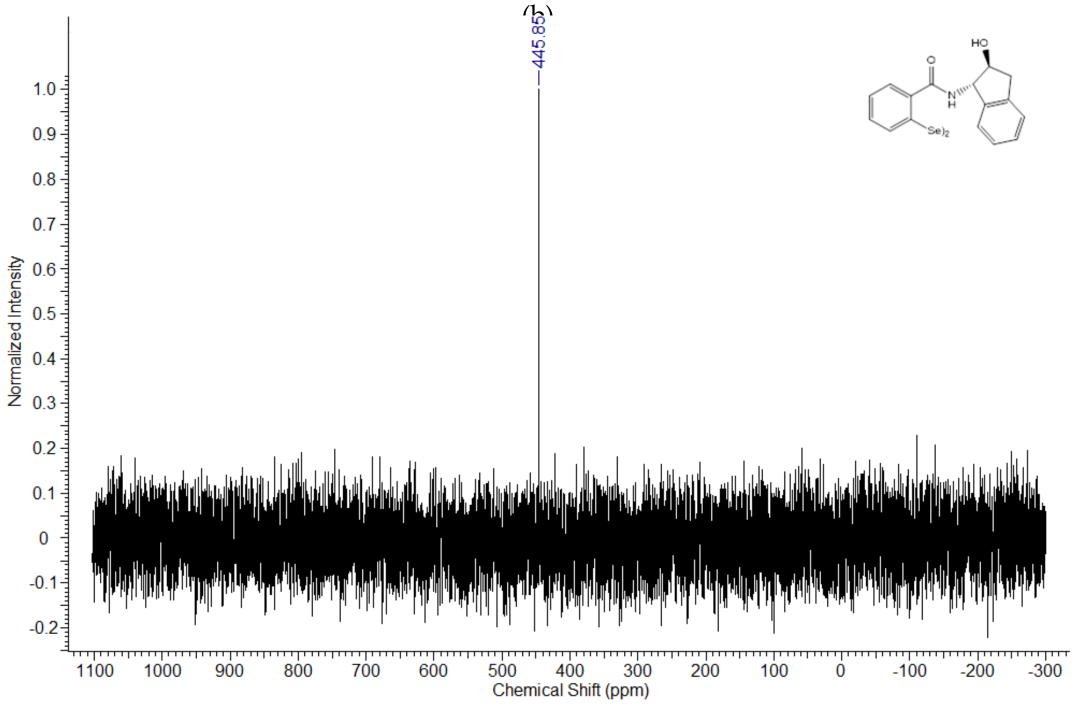

(c)

Figure S27. (**a**) 1H NMR, (**b**) 13C NMR, and (**c**) 77Se NMR spectra of 2,2′-diselenobis[*N*-(1*S*,2*S*)-(-)-*trans*-2-hydroxy-1-indanylbezamide] **30b.**

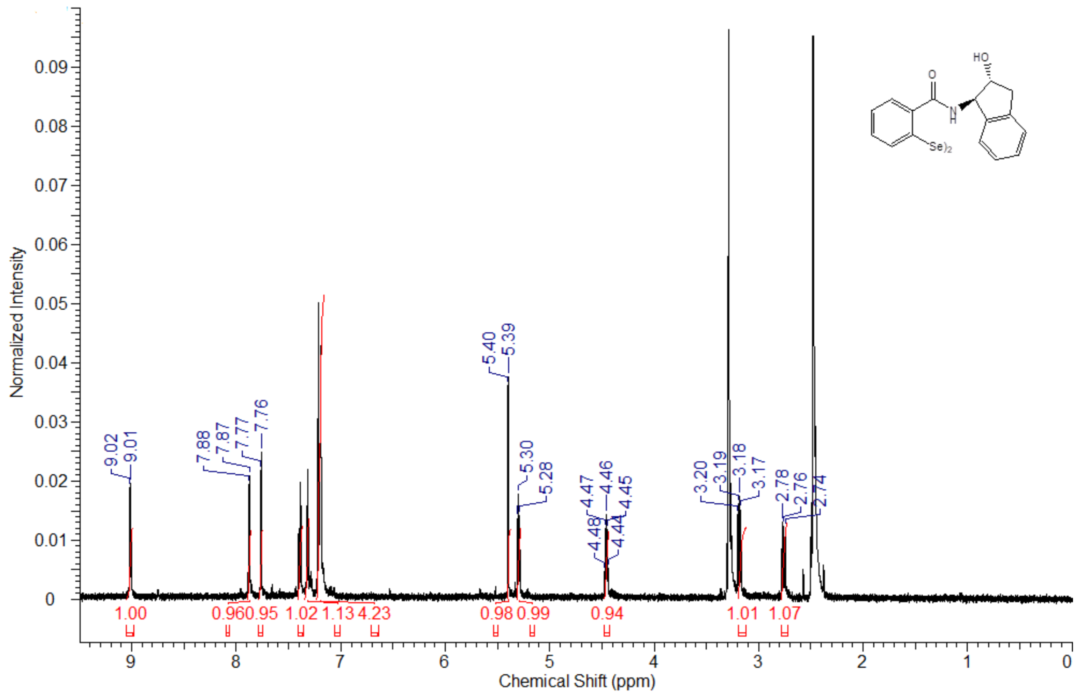

Normalized Intensity
Chemical Shift (ppm)
9.02
9.01
7.88
7.87
7.77
7.76
5.40
5.39
5.30
5.28
4.47
4.46
4.45
3.20
3.19
3.18
3.17
2.78
2.76
2.74
1.00
0.96
0.95
1.02
1.13
4.23
0.98
0.99
0.94
1.01
1.07
HO
O
N
H
Se)2

(a)

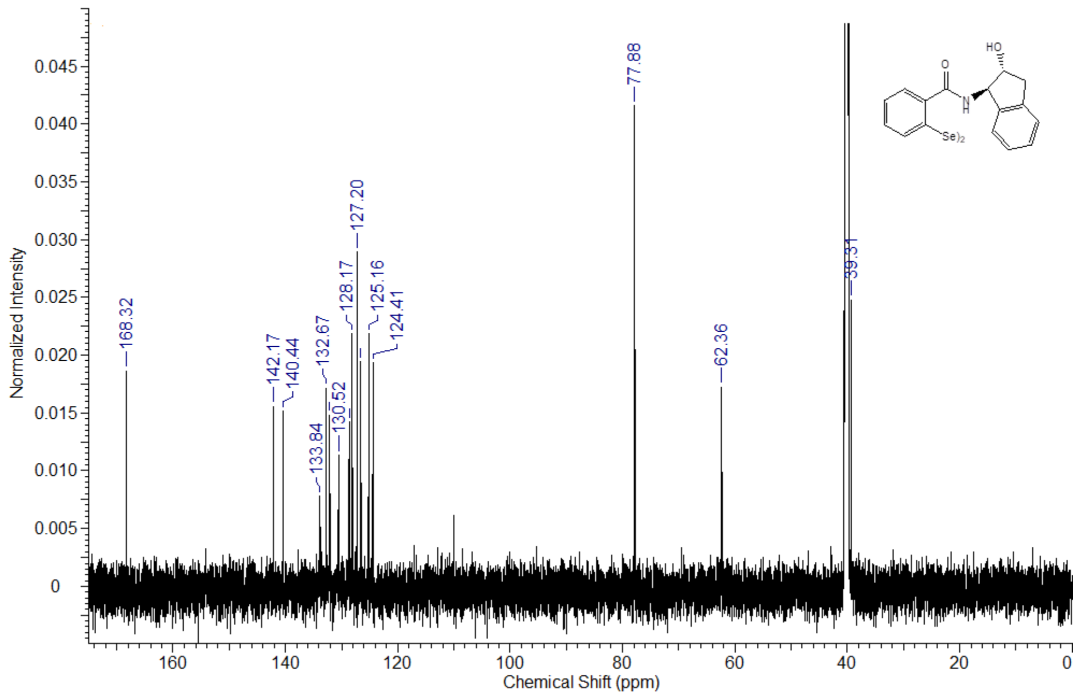

Normalized Intensity
Chemical Shift (ppm)
168.32
142.17
140.44
133.84
132.67
130.52
128.17
127.20
125.16
124.41
77.88
62.36
39.31
HO
O
N
H
Se)2

(b)

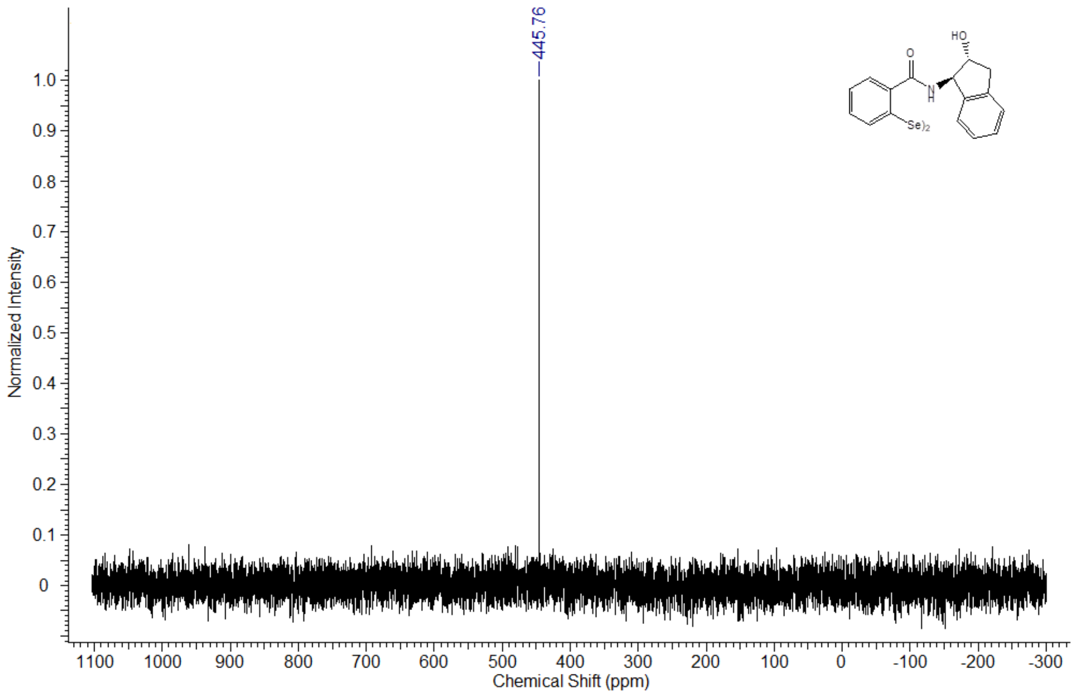

(c)

Figure S28. (**a**) 1H NMR, (**b**) 13C NMR, and (**c**) 77Se NMR spectra of 2,2′-diselenobis[*N*-(*1R,2R*)-(-)-*trans*-2-hydroxy-1-indanylbezamide] **31b.**

3. Crystallographic data of benzisoselenazolo-3(2H)-ones 24a and 25a

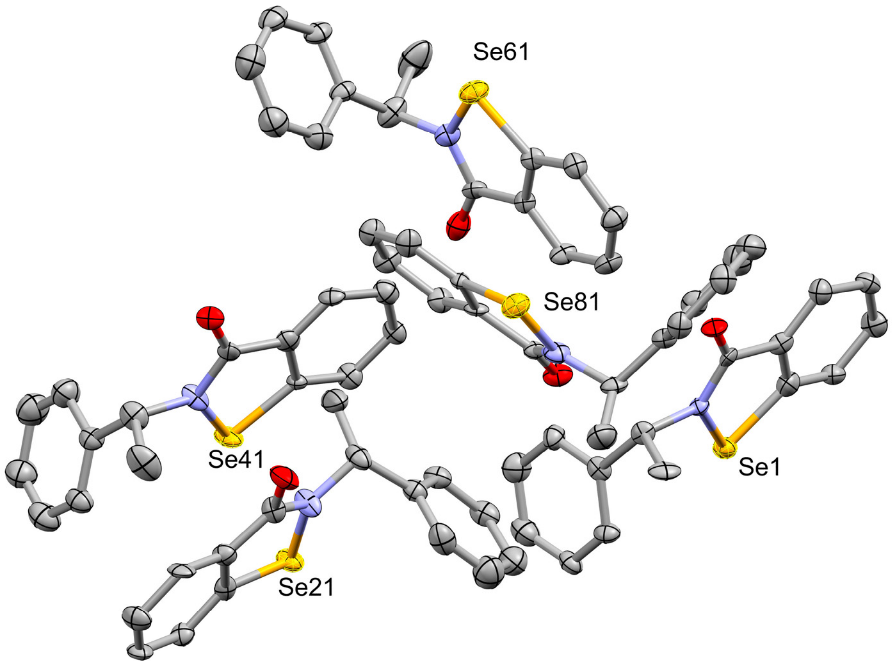

Figure S29. Asymmetric part of the structure **24a**. Selenium atoms are labelled to identify molecules 1-5. ADPs are plotted at 50% probability level. Hydrogen atoms are omitted for clarity.

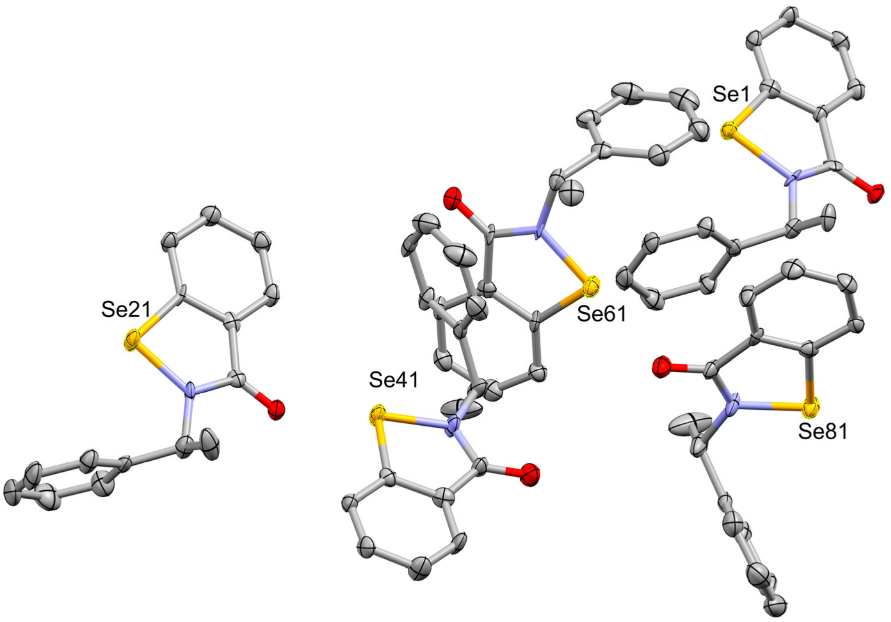

Figure S30. Asymmetric part of the structure **25a**. Selenium atoms are labelled to identify molecules 1-5. ADPs are plotted at 50% probability level. Hydrogen atoms are omitted for clarity.

Table S1. Crystal data and structure refinement for **24a**.

Identification code	**24a**
Empirical formula	C15 H13 N O Se
Formula weight	302.22
Temperature	100(2) K
Wavelength	1.54184 Å
Crystal system	Orthorhombic
Space group	$P2_12_12_1$
Unit cell dimensions	a = 10.0996(2) Å
	b = 52.3461(11) Å
	c = 12.6231(3) Å
Volume	6673.5(2) Å^3
Z	20
Density (calculated)	1.504 Mg/m^3
Absorption coefficient	3.696 mm^{-1}
F(000)	3040
Crystal size	0.138 x 0.110 x 0.065 mm^3
Theta range for data collection	3.377 to 77.944°.
Index ranges	-12<=h<=12, -66<=k<=47, -15<=l<=15
Reflections collected	85045
Independent reflections	13902 [R(int) = 0.0918]
Completeness to theta = 67.684°	99.9 %
Absorption correction	Semi-empirical from equivalents

Max. and min. transmission	1.00000 and 0.62936
Refinement method	Full-matrix least-squares on F^2
Data / restraints / parameters	13902 / 540 / 811
Goodness-of-fit on F^2	1.140
Final R indices [I>2sigma(I)]	R1 = 0.1055, wR2 = 0.2987
R indices (all data)	R1 = 0.1079, wR2 = 0.3013
Absolute structure parameter	0.042(10)
Extinction coefficient	n/a
Largest diff. peak and hole	3.609 and -2.148 e.Å^{-3}

Table S2. Bond lengths [Å] and angles [°] for **24a**.

Se(1)-C(10)	1.91(2)
Se(1)-N(2)	1.932(14)
N(2)-C(3)	1.31(2)
N(2)-C(11)	1.51(2)
C(3)-O(4)	1.19(2)
C(3)-C(5)	1.52(3)
C(5)-C(10)	1.39(3)
C(5)-C(6)	1.40(3)
C(6)-C(7)	1.39(3)
C(7)-C(8)	1.39(3)
C(8)-C(9)	1.40(3)
C(9)-C(10)	1.37(3)
C(11)-C(12)	1.53(3)
C(11)-C(13)	1.54(3)
C(13)-C(14)	1.37(3)
C(13)-C(18)	1.40(3)
C(14)-C(15)	1.40(3)
C(15)-C(16)	1.40(3)
C(16)-C(17)	1.35(3)
C(17)-C(18)	1.37(3)
Se(21)-C(30)	1.882(19)
Se(21)-N(22)	1.900(15)
N(22)-C(23)	1.36(2)
N(22)-C(31)	1.47(3)
C(23)-O(24)	1.23(3)
C(23)-C(25)	1.43(3)
C(25)-C(26)	1.31(3)
C(25)-C(30)	1.47(3)
C(26)-C(27)	1.42(3)
C(27)-C(28)	1.39(3)
C(28)-C(29)	1.38(3)
C(29)-C(30)	1.38(3)
C(31)-C(32)	1.52(4)
C(31)-C(33)	1.52(3)
C(33)-C(34)	1.36(3)
C(33)-C(38)	1.39(3)

C(34)-C(35)	1.40(3)
C(35)-C(36)	1.39(4)
C(36)-C(37)	1.39(4)
C(37)-C(38)	1.33(4)
Se(41)-N(42)	1.858(17)
Se(41)-C(50)	1.887(17)
N(42)-C(43)	1.33(2)
N(42)-C(51)	1.48(3)
C(43)-O(44)	1.26(2)
C(43)-C(45)	1.46(3)
C(45)-C(46)	1.36(3)
C(45)-C(50)	1.44(3)
C(46)-C(47)	1.40(3)
C(47)-C(48)	1.40(3)
C(48)-C(49)	1.39(3)
C(49)-C(50)	1.38(2)
C(51)-C(52)	1.50(3)
C(51)-C(53)	1.55(3)
C(53)-C(54)	1.38(3)
C(53)-C(58)	1.40(3)
C(54)-C(55)	1.38(3)
C(55)-C(56)	1.35(4)
C(56)-C(57)	1.39(4)
C(57)-C(58)	1.35(4)
Se(61)-N(62)	1.891(16)
Se(61)-C(70)	1.905(18)
N(62)-C(63)	1.33(2)
N(62)-C(71)	1.47(3)
C(63)-O(64)	1.23(2)
C(63)-C(65)	1.49(2)
C(65)-C(70)	1.35(2)
C(65)-C(66)	1.38(3)
C(66)-C(67)	1.41(3)
C(67)-C(68)	1.39(3)
C(68)-C(69)	1.41(3)
C(69)-C(70)	1.40(3)
C(71)-C(73)	1.50(3)
C(71)-C(72)	1.55(4)
C(73)-C(74)	1.36(3)
C(73)-C(78)	1.40(3)
C(74)-C(75)	1.39(3)
C(75)-C(76)	1.35(4)
C(76)-C(77)	1.37(4)
C(77)-C(78)	1.37(3)
Se(81)-C(90)	1.877(17)
Se(81)-N(82)	1.879(17)
N(82)-C(83)	1.34(2)
N(82)-C(91)	1.44(3)
C(83)-O(84)	1.27(2)

C(83)-C(85)	1.51(2)
C(85)-C(86)	1.38(3)
C(85)-C(90)	1.45(2)
C(86)-C(87)	1.45(3)
C(87)-C(88)	1.36(3)
C(88)-C(89)	1.40(3)
C(89)-C(90)	1.37(3)
C(91)-C(92)	1.52(3)
C(91)-C(93)	1.53(3)
C(93)-C(98)	1.39(3)
C(93)-C(94)	1.40(3)
C(94)-C(95)	1.38(3)
C(95)-C(96)	1.37(3)
C(96)-C(97)	1.40(4)
C(97)-C(98)	1.35(3)
C(10)-Se(1)-N(2)	83.0(8)
C(3)-N(2)-C(11)	121.2(15)
C(3)-N(2)-Se(1)	120.6(13)
C(11)-N(2)-Se(1)	118.3(11)
O(4)-C(3)-N(2)	125.1(19)
O(4)-C(3)-C(5)	127.7(18)
N(2)-C(3)-C(5)	107.1(16)
C(10)-C(5)-C(6)	120.7(19)
C(10)-C(5)-C(3)	117.6(18)
C(6)-C(5)-C(3)	121.7(17)
C(7)-C(6)-C(5)	118(2)
C(8)-C(7)-C(6)	121(2)
C(7)-C(8)-C(9)	120(2)
C(10)-C(9)-C(8)	119(2)
C(9)-C(10)-C(5)	121(2)
C(9)-C(10)-Se(1)	127.0(17)
C(5)-C(10)-Se(1)	111.6(16)
N(2)-C(11)-C(12)	110.4(16)
N(2)-C(11)-C(13)	111.0(14)
C(12)-C(11)-C(13)	114.0(16)
C(14)-C(13)-C(18)	118.6(18)
C(14)-C(13)-C(11)	119.9(18)
C(18)-C(13)-C(11)	121.5(17)
C(13)-C(14)-C(15)	121(2)
C(16)-C(15)-C(14)	118(2)
C(17)-C(16)-C(15)	120(2)
C(16)-C(17)-C(18)	121(2)
C(17)-C(18)-C(13)	120.5(19)
C(30)-Se(21)-N(22)	84.8(8)
C(23)-N(22)-C(31)	125.1(16)
C(23)-N(22)-Se(21)	116.3(13)
C(31)-N(22)-Se(21)	118.5(12)
O(24)-C(23)-N(22)	121.6(19)
O(24)-C(23)-C(25)	125.6(18)

N(22)-C(23)-C(25)	112.8(17)
C(26)-C(25)-C(23)	129(2)
C(26)-C(25)-C(30)	117.5(19)
C(23)-C(25)-C(30)	113.5(17)
C(25)-C(26)-C(27)	123(2)
C(28)-C(27)-C(26)	119(2)
C(29)-C(28)-C(27)	120.3(19)
C(30)-C(29)-C(28)	120.0(19)
C(29)-C(30)-C(25)	120.1(17)
C(29)-C(30)-Se(21)	127.6(15)
C(25)-C(30)-Se(21)	112.3(14)
N(22)-C(31)-C(32)	110.5(19)
N(22)-C(31)-C(33)	109.0(17)
C(32)-C(31)-C(33)	115.5(19)
C(34)-C(33)-C(38)	118(2)
C(34)-C(33)-C(31)	123.2(19)
C(38)-C(33)-C(31)	118(2)
C(33)-C(34)-C(35)	121(2)
C(36)-C(35)-C(34)	119(2)
C(37)-C(36)-C(35)	118(2)
C(38)-C(37)-C(36)	122(3)
C(37)-C(38)-C(33)	121(2)
N(42)-Se(41)-C(50)	85.4(7)
C(43)-N(42)-C(51)	120.7(17)
C(43)-N(42)-Se(41)	117.2(14)
C(51)-N(42)-Se(41)	122.1(13)
O(44)-C(43)-N(42)	124.1(18)
O(44)-C(43)-C(45)	123.1(18)
N(42)-C(43)-C(45)	112.6(18)
C(46)-C(45)-C(50)	119.0(18)
C(46)-C(45)-C(43)	127.4(19)
C(50)-C(45)-C(43)	113.5(17)
C(45)-C(46)-C(47)	120.6(18)
C(46)-C(47)-C(48)	119.8(19)
C(49)-C(48)-C(47)	120.7(19)
C(50)-C(49)-C(48)	118.8(18)
C(49)-C(50)-C(45)	120.9(17)
C(49)-C(50)-Se(41)	127.8(14)
C(45)-C(50)-Se(41)	111.2(13)
N(42)-C(51)-C(52)	109.7(19)
N(42)-C(51)-C(53)	107.7(16)
C(52)-C(51)-C(53)	115(2)
C(54)-C(53)-C(58)	118(2)
C(54)-C(53)-C(51)	124(2)
C(58)-C(53)-C(51)	118.1(19)
C(53)-C(54)-C(55)	120(2)
C(56)-C(55)-C(54)	122(2)
C(55)-C(56)-C(57)	119(2)
C(55)-C(56)-H(56A)	120.3

C(57)-C(56)-H(56A)	120.3
C(58)-C(57)-C(56)	120(3)
C(57)-C(58)-C(53)	121(2)
N(62)-Se(61)-C(70)	83.7(7)
C(63)-N(62)-C(71)	122.0(17)
C(63)-N(62)-Se(61)	118.3(13)
C(71)-N(62)-Se(61)	119.0(13)
O(64)-C(63)-N(62)	125.6(18)
O(64)-C(63)-C(65)	125.6(17)
N(62)-C(63)-C(65)	108.7(16)
C(70)-C(65)-C(66)	118.9(17)
C(70)-C(65)-C(63)	117.0(17)
C(66)-C(65)-C(63)	124.1(17)
C(65)-C(66)-C(67)	120.6(18)
C(68)-C(67)-C(66)	119.0(19)
C(67)-C(68)-C(69)	121(2)
C(70)-C(69)-C(68)	117.1(19)
C(65)-C(70)-C(69)	123.5(18)
C(65)-C(70)-Se(61)	112.2(13)
C(69)-C(70)-Se(61)	124.1(14)
N(62)-C(71)-C(73)	110.3(17)
N(62)-C(71)-C(72)	107(2)
C(73)-C(71)-C(72)	114(2)
C(74)-C(73)-C(78)	118(2)
C(74)-C(73)-C(71)	125(2)
C(78)-C(73)-C(71)	117.4(19)
C(73)-C(74)-C(75)	120(2)
C(76)-C(75)-C(74)	123(2)
C(75)-C(76)-C(77)	118(2)
C(76)-C(77)-C(78)	120(3)
C(77)-C(78)-C(73)	121(2)
C(90)-Se(81)-N(82)	86.5(7)
C(83)-N(82)-C(91)	122.6(16)
C(83)-N(82)-Se(81)	117.7(13)
C(91)-N(82)-Se(81)	119.5(13)
O(84)-C(83)-N(82)	126.6(16)
O(84)-C(83)-C(85)	122.9(15)
N(82)-C(83)-C(85)	110.4(15)
C(86)-C(85)-C(90)	121.8(17)
C(86)-C(85)-C(83)	124.0(16)
C(90)-C(85)-C(83)	114.2(15)
C(85)-C(86)-C(87)	115.0(18)
C(88)-C(87)-C(86)	123(2)
C(87)-C(88)-C(89)	121(2)
C(90)-C(89)-C(88)	119.0(19)
C(89)-C(90)-C(85)	120.3(17)
C(89)-C(90)-Se(81)	128.6(14)
C(85)-C(90)-Se(81)	111.1(12)
N(82)-C(91)-C(92)	111.1(18)

N(82)-C(91)-C(93)	109.8(15)
C(92)-C(91)-C(93)	114.3(18)
C(98)-C(93)-C(94)	117.7(19)
C(98)-C(93)-C(91)	118.6(19)
C(94)-C(93)-C(91)	123.5(18)
C(95)-C(94)-C(93)	121(2)
C(96)-C(95)-C(94)	118(2)
C(95)-C(96)-C(97)	122(2)
C(98)-C(97)-C(96)	119(2)
C(97)-C(98)-C(93)	122(2)

Table S3. Torsion angles [°] for **24a**.

C(11)-N(2)-C(3)-O(4)	-3(3)
Se(1)-N(2)-C(3)-O(4)	176.7(15)
C(11)-N(2)-C(3)-C(5)	-179.0(14)
Se(1)-N(2)-C(3)-C(5)	0.5(19)
O(4)-C(3)-C(5)-C(10)	-178.6(19)
N(2)-C(3)-C(5)-C(10)	-3(2)
O(4)-C(3)-C(5)-C(6)	2(3)
N(2)-C(3)-C(5)-C(6)	177.9(17)
C(10)-C(5)-C(6)-C(7)	-1(3)
C(3)-C(5)-C(6)-C(7)	178.8(17)
C(5)-C(6)-C(7)-C(8)	2(3)
C(6)-C(7)-C(8)-C(9)	0(3)
C(7)-C(8)-C(9)-C(10)	-2(3)
C(8)-C(9)-C(10)-C(5)	3(3)
C(8)-C(9)-C(10)-Se(1)	177.8(16)
C(6)-C(5)-C(10)-C(9)	-2(3)
C(3)-C(5)-C(10)-C(9)	178.7(17)
C(6)-C(5)-C(10)-Se(1)	-177.1(14)
C(3)-C(5)-C(10)-Se(1)	3(2)
C(3)-N(2)-C(11)-C(12)	-78(2)
Se(1)-N(2)-C(11)-C(12)	102.1(15)
C(3)-N(2)-C(11)-C(13)	154.3(16)
Se(1)-N(2)-C(11)-C(13)	-25.3(19)
N(2)-C(11)-C(13)-C(14)	-86(2)
C(12)-C(11)-C(13)-C(14)	148.5(19)
N(2)-C(11)-C(13)-C(18)	90(2)
C(12)-C(11)-C(13)-C(18)	-35(2)
C(18)-C(13)-C(14)-C(15)	-1(3)
C(11)-C(13)-C(14)-C(15)	175.5(19)
C(13)-C(14)-C(15)-C(16)	1(3)
C(14)-C(15)-C(16)-C(17)	-2(3)
C(15)-C(16)-C(17)-C(18)	2(3)
C(16)-C(17)-C(18)-C(13)	-2(3)
C(14)-C(13)-C(18)-C(17)	1(3)
C(11)-C(13)-C(18)-C(17)	-174.9(18)
C(31)-N(22)-C(23)-O(24)	-3(3)
Se(21)-N(22)-C(23)-O(24)	175.0(17)

C(31)-N(22)-C(23)-C(25)	176(2)
Se(21)-N(22)-C(23)-C(25)	-7(2)
O(24)-C(23)-C(25)-C(26)	1(4)
N(22)-C(23)-C(25)-C(26)	-177(2)
O(24)-C(23)-C(25)-C(30)	-177(2)
N(22)-C(23)-C(25)-C(30)	5(3)
C(23)-C(25)-C(26)-C(27)	-177(2)
C(30)-C(25)-C(26)-C(27)	1(3)
C(25)-C(26)-C(27)-C(28)	2(4)
C(26)-C(27)-C(28)-C(29)	-5(3)
C(27)-C(28)-C(29)-C(30)	5(3)
C(28)-C(29)-C(30)-C(25)	-1(3)
C(28)-C(29)-C(30)-Se(21)	176.3(15)
C(26)-C(25)-C(30)-C(29)	-1(3)
C(23)-C(25)-C(30)-C(29)	177.1(18)
C(26)-C(25)-C(30)-Se(21)	-179.5(16)
C(23)-C(25)-C(30)-Se(21)	-1(2)
N(22)-Se(21)-C(30)-C(29)	180.0(19)
N(22)-Se(21)-C(30)-C(25)	-2.1(14)
C(23)-N(22)-C(31)-C(32)	-100(3)
Se(21)-N(22)-C(31)-C(32)	83(2)
C(23)-N(22)-C(31)-C(33)	132(2)
Se(21)-N(22)-C(31)-C(33)	-45(2)
N(22)-C(31)-C(33)-C(34)	115(2)
C(32)-C(31)-C(33)-C(34)	-10(3)
N(22)-C(31)-C(33)-C(38)	-60(3)
C(32)-C(31)-C(33)-C(38)	175(2)
C(38)-C(33)-C(34)-C(35)	3(3)
C(31)-C(33)-C(34)-C(35)	-173(2)
C(33)-C(34)-C(35)-C(36)	-4(3)
C(34)-C(35)-C(36)-C(37)	4(4)
C(35)-C(36)-C(37)-C(38)	-2(4)
C(36)-C(37)-C(38)-C(33)	1(4)
C(34)-C(33)-C(38)-C(37)	-1(3)
C(31)-C(33)-C(38)-C(37)	175(2)
C(50)-Se(41)-N(42)-C(43)	0.6(15)
C(50)-Se(41)-N(42)-C(51)	-179.3(16)
C(51)-N(42)-C(43)-O(44)	-5(3)
Se(41)-N(42)-C(43)-O(44)	175.2(15)
C(51)-N(42)-C(43)-C(45)	179.4(18)
Se(41)-N(42)-C(43)-C(45)	0(2)
O(44)-C(43)-C(45)-C(46)	3(3)
N(42)-C(43)-C(45)-C(46)	178.8(19)
O(44)-C(43)-C(45)-C(50)	-175.7(17)
N(42)-C(43)-C(45)-C(50)	0(2)
C(50)-C(45)-C(46)-C(47)	-1(3)
C(43)-C(45)-C(46)-C(47)	-179.4(19)
C(45)-C(46)-C(47)-C(48)	1(3)
C(46)-C(47)-C(48)-C(49)	1(3)

C(47)-C(48)-C(49)-C(50)	-2(3)
C(48)-C(49)-C(50)-C(45)	3(3)
C(48)-C(49)-C(50)-Se(41)	179.6(15)
C(46)-C(45)-C(50)-C(49)	-1(3)
C(43)-C(45)-C(50)-C(49)	177.8(17)
C(46)-C(45)-C(50)-Se(41)	-178.5(14)
C(43)-C(45)-C(50)-Se(41)	0(2)
N(42)-Se(41)-C(50)-C(49)	-177.7(18)
N(42)-Se(41)-C(50)-C(45)	-0.5(14)
C(43)-N(42)-C(51)-C(52)	-86(3)
Se(41)-N(42)-C(51)-C(52)	94(2)
C(43)-N(42)-C(51)-C(53)	148.1(18)
Se(41)-N(42)-C(51)-C(53)	-32(2)
N(42)-C(51)-C(53)-C(54)	103(2)
C(52)-C(51)-C(53)-C(54)	-20(3)
N(42)-C(51)-C(53)-C(58)	-75(2)
C(52)-C(51)-C(53)-C(58)	162(2)
C(58)-C(53)-C(54)-C(55)	3(3)
C(51)-C(53)-C(54)-C(55)	-176(2)
C(53)-C(54)-C(55)-C(56)	-1(4)
C(54)-C(55)-C(56)-C(57)	0(4)
C(55)-C(56)-C(57)-C(58)	-2(4)
C(56)-C(57)-C(58)-C(53)	4(4)
C(54)-C(53)-C(58)-C(57)	-4(4)
C(51)-C(53)-C(58)-C(57)	174(2)
C(70)-Se(61)-N(62)-C(63)	3.7(15)
C(70)-Se(61)-N(62)-C(71)	174.7(17)
C(71)-N(62)-C(63)-O(64)	1(3)
Se(61)-N(62)-C(63)-O(64)	172.2(16)
C(71)-N(62)-C(63)-C(65)	-174.5(18)
Se(61)-N(62)-C(63)-C(65)	-4(2)
O(64)-C(63)-C(65)-C(70)	-174.3(19)
N(62)-C(63)-C(65)-C(70)	2(2)
O(64)-C(63)-C(65)-C(66)	5(3)
N(62)-C(63)-C(65)-C(66)	-179.0(18)
C(70)-C(65)-C(66)-C(67)	2(3)
C(63)-C(65)-C(66)-C(67)	-177.6(18)
C(65)-C(66)-C(67)-C(68)	0(3)
C(66)-C(67)-C(68)-C(69)	1(3)
C(67)-C(68)-C(69)-C(70)	-3(3)
C(66)-C(65)-C(70)-C(69)	-4(3)
C(63)-C(65)-C(70)-C(69)	175.8(17)
C(66)-C(65)-C(70)-Se(61)	-178.3(15)
C(63)-C(65)-C(70)-Se(61)	1(2)
C(68)-C(69)-C(70)-C(65)	4(3)
C(68)-C(69)-C(70)-Se(61)	178.0(15)
C(63)-N(62)-C(71)-C(73)	131(2)
Se(61)-N(62)-C(71)-C(73)	-40(2)
C(63)-N(62)-C(71)-C(72)	-105(2)

Se(61)-N(62)-C(71)-C(72)	85(2)
N(62)-C(71)-C(73)-C(74)	121(2)
C(72)-C(71)-C(73)-C(74)	0(3)
N(62)-C(71)-C(73)-C(78)	-61(3)
C(72)-C(71)-C(73)-C(78)	178(2)
C(78)-C(73)-C(74)-C(75)	3(3)
C(71)-C(73)-C(74)-C(75)	-179(2)
C(73)-C(74)-C(75)-C(76)	-2(4)
C(74)-C(75)-C(76)-C(77)	0(4)
C(75)-C(76)-C(77)-C(78)	-1(4)
C(76)-C(77)-C(78)-C(73)	2(4)
C(74)-C(73)-C(78)-C(77)	-3(3)
C(71)-C(73)-C(78)-C(77)	178(2)
C(90)-Se(81)-N(82)-C(83)	0.6(15)
C(90)-Se(81)-N(82)-C(91)	175.5(15)
C(91)-N(82)-C(83)-O(84)	1(3)
Se(81)-N(82)-C(83)-O(84)	175.5(15)
C(91)-N(82)-C(83)-C(85)	-176.4(16)
Se(81)-N(82)-C(83)-C(85)	-2(2)
O(84)-C(83)-C(85)-C(86)	6(3)
N(82)-C(83)-C(85)-C(86)	-176.4(18)
O(84)-C(83)-C(85)-C(90)	-175.0(16)
N(82)-C(83)-C(85)-C(90)	2(2)
C(90)-C(85)-C(86)-C(87)	2(3)
C(83)-C(85)-C(86)-C(87)	-179.8(17)
C(85)-C(86)-C(87)-C(88)	-2(3)
C(86)-C(87)-C(88)-C(89)	1(4)
C(87)-C(88)-C(89)-C(90)	0(3)
C(88)-C(89)-C(90)-C(85)	0(3)
C(88)-C(89)-C(90)-Se(81)	-177.4(15)
C(86)-C(85)-C(90)-C(89)	-1(3)
C(83)-C(85)-C(90)-C(89)	-179.5(16)
C(86)-C(85)-C(90)-Se(81)	176.8(15)
C(83)-C(85)-C(90)-Se(81)	-1.8(19)
N(82)-Se(81)-C(90)-C(89)	178.2(18)
N(82)-Se(81)-C(90)-C(85)	0.8(13)
C(83)-N(82)-C(91)-C(92)	-121(2)
Se(81)-N(82)-C(91)-C(92)	65(2)
C(83)-N(82)-C(91)-C(93)	112(2)
Se(81)-N(82)-C(91)-C(93)	-63(2)
N(82)-C(91)-C(93)-C(98)	-76(2)
C(92)-C(91)-C(93)-C(98)	158.4(19)
N(82)-C(91)-C(93)-C(94)	99(2)
C(92)-C(91)-C(93)-C(94)	-26(3)
C(98)-C(93)-C(94)-C(95)	0(3)
C(91)-C(93)-C(94)-C(95)	-176(2)
C(93)-C(94)-C(95)-C(96)	0(3)
C(94)-C(95)-C(96)-C(97)	-1(4)
C(95)-C(96)-C(97)-C(98)	3(4)

C(96)-C(97)-C(98)-C(93)	-3(4)
C(94)-C(93)-C(98)-C(97)	2(3)
C(91)-C(93)-C(98)-C(97)	178(2)

Table S4. Hydrogen bonds for **24a** [Å and °].

D-H...A	d(D-H)	d(H...A)	d(D...A)	<(DHA)
C(9)-H(9A)...O(4)#1	0.95	2.27	2.95(2)	128.1
C(29)-H(29A)...O(44)#2	0.95	2.36	3.05(2)	128.5
C(49)-H(49A)...O(24)	0.95	2.35	3.03(3)	127.6
C(69)-H(69A)...O(84)#3	0.95	2.45	3.16(3)	131.6
C(89)-H(89A)...O(64)	0.95	2.44	3.11(3)	127.4

Symmetry transformations used to generate equivalent atoms:.
#1 -x+3/2,-y+1,z+1/2 #2 x,y,z+1 #3 x,y,z-1 .

Table S5. Crystal data and structure refinement for **25a**.

Identification code	**25a**
Empirical formula	C15 H13 N O Se
Formula weight	302.22
Temperature	100(2) K
Wavelength	1.54184 Å
Crystal system	Orthorhombic
Space group	$P2_12_12_1$
Unit cell dimensions	a = 10.1164(3) Å
	b = 12.6176(4) Å
	c = 52.3764(15) Å
Volume	6685.6(3) $Å^3$
Z	20
Density (calculated)	1.501 Mg/m^3
Absorption coefficient	3.690 mm^{-1}
F(000)	3040
Crystal size	0.175 x 0.102 x 0.051 mm^3
Theta range for data collection	3.375 to 77.905°.
Index ranges	-12<=h<=12, -15<=k<=13, -64<=l<=25
Reflections collected	31953
Independent reflections	12315 [R(int) = 0.0329]
Completeness to theta = 67.684°	99.6 %
Absorption correction	Semi-empirical from equivalents
Max. and min. transmission	1.00000 and 0.87047
Refinement method	Full-matrix least-squares on F^2
Data / restraints / parameters	12315 / 6 / 816
Goodness-of-fit on F^2	1.096
Final R indices [I>2sigma(I)]	R1 = 0.0492, wR2 = 0.1213
R indices (all data)	R1 = 0.0502, wR2 = 0.1218
Absolute structure parameter	0.011(9)
Extinction coefficient	n/a

Largest diff. peak and hole	1.291 and -0.900 e.Å^{-3}

Table S6. Bond lengths [Å] and angles [°] for **25a**.

Se(1)-C(10)	1.900(8)
Se(1)-N(2)	1.922(6)
N(2)-C(3)	1.289(11)
N(2)-C(11)	1.492(10)
C(3)-O(4)	1.248(10)
C(3)-C(5)	1.493(11)
C(5)-C(10)	1.388(11)
C(5)-C(6)	1.401(11)
C(6)-C(7)	1.370(13)
C(7)-C(8)	1.396(13)
C(8)-C(9)	1.386(12)
C(9)-C(10)	1.391(12)
C(11)-C(13)	1.519(11)
C(11)-C(12)	1.531(11)
C(13)-C(14)	1.388(12)
C(13)-C(18)	1.397(12)
C(14)-C(15)	1.393(12)
C(15)-C(16)	1.393(14)
C(16)-C(17)	1.364(13)
C(17)-C(18)	1.388(12)
Se(21)-N(22)	1.877(7)
Se(21)-C(30)	1.895(8)
N(22)-C(23)	1.364(10)
N(22)-C(31)	1.478(11)
C(23)-O(24)	1.237(10)
C(23)-C(25)	1.461(11)
C(25)-C(26)	1.387(11)
C(25)-C(30)	1.409(11)
C(26)-C(27)	1.380(12)
C(27)-C(28)	1.399(13)
C(28)-C(29)	1.393(12)
C(29)-C(30)	1.392(11)
C(31)-C(32)	1.519(14)
C(31)-C(33)	1.525(12)
C(33)-C(38)	1.376(12)
C(33)-C(34)	1.398(13)
C(34)-C(35)	1.376(14)
C(35)-C(36)	1.371(16)
C(36)-C(37)	1.363(17)
C(37)-C(38)	1.396(14)
Se(41)-C(50)	1.882(8)
Se(41)-N(42)	1.890(7)
N(42)-C(43)	1.353(11)
N(42)-C(51)	1.483(11)

C(43)-O(44)	1.229(11)
C(43)-C(45)	1.451(12)
C(45)-C(46)	1.371(12)
C(45)-C(50)	1.423(12)
C(46)-C(47)	1.383(12)
C(47)-C(48)	1.390(13)
C(48)-C(49)	1.377(12)
C(49)-C(50)	1.389(11)
C(51)-C(53)	1.511(12)
C(51)-C(52)	1.528(17)
C(53)-C(58)	1.379(12)
C(53)-C(54)	1.392(13)
C(54)-C(55)	1.376(15)
C(55)-C(56)	1.384(16)
C(56)-C(57)	1.370(15)
C(57)-C(58)	1.412(13)
Se(61)-N(62)	1.882(6)
Se(61)-C(70)	1.887(8)
N(62)-C(63)	1.359(10)
N(62)-C(71)	1.454(11)
C(63)-O(64)	1.248(9)
C(63)-C(65)	1.488(11)
C(65)-C(66)	1.402(11)
C(65)-C(70)	1.403(11)
C(66)-C(67)	1.394(13)
C(67)-C(68)	1.388(14)
C(68)-C(69)	1.378(12)
C(69)-C(70)	1.387(11)
C(71)-C(73)	1.507(12)
C(71)-C(72)	1.527(13)
C(73)-C(78)	1.392(12)
C(73)-C(74)	1.407(13)
C(74)-C(75)	1.385(13)
C(75)-C(76)	1.362(15)
C(76)-C(77)	1.399(16)
C(77)-C(78)	1.365(14)
Se(81)-N(82)	1.882(7)
Se(81)-C(90)	1.905(8)
N(82)-C(83)	1.337(11)
N(82)-C(91)	1.469(11)
C(83)-O(84)	1.232(10)
C(83)-C(85)	1.484(11)
C(85)-C(90)	1.355(12)
C(85)-C(86)	1.399(12)
C(86)-C(87)	1.381(13)
C(87)-C(88)	1.386(14)
C(88)-C(89)	1.388(13)
C(89)-C(90)	1.404(11)
C(91)-C(93)	1.504(12)

C(91)-C(92)	1.527(17)
C(93)-C(98)	1.380(12)
C(93)-C(94)	1.391(14)
C(94)-C(95)	1.391(14)
C(95)-C(96)	1.374(17)
C(96)-C(97)	1.375(16)
C(97)-C(98)	1.387(14)
C(10)-Se(1)-N(2)	83.7(3)
C(3)-N(2)-C(11)	123.3(6)
C(3)-N(2)-Se(1)	117.6(5)
C(11)-N(2)-Se(1)	119.0(5)
O(4)-C(3)-N(2)	123.6(7)
O(4)-C(3)-C(5)	124.8(7)
N(2)-C(3)-C(5)	111.4(7)
C(10)-C(5)-C(6)	120.2(8)
C(10)-C(5)-C(3)	114.9(7)
C(6)-C(5)-C(3)	124.9(7)
C(7)-C(6)-C(5)	119.1(8)
C(6)-C(7)-C(8)	120.7(8)
C(9)-C(8)-C(7)	120.7(8)
C(8)-C(9)-C(10)	118.7(8)
C(5)-C(10)-C(9)	120.7(8)
C(5)-C(10)-Se(1)	112.2(6)
C(9)-C(10)-Se(1)	127.1(7)
N(2)-C(11)-C(13)	111.1(6)
N(2)-C(11)-C(12)	109.4(7)
C(13)-C(11)-C(12)	114.2(7)
C(14)-C(13)-C(18)	119.3(8)
C(14)-C(13)-C(11)	119.7(8)
C(18)-C(13)-C(11)	121.0(7)
C(13)-C(14)-C(15)	120.9(8)
C(16)-C(15)-C(14)	119.1(8)
C(17)-C(16)-C(15)	120.1(8)
C(16)-C(17)-C(18)	121.4(8)
C(17)-C(18)-C(13)	119.2(8)
N(22)-Se(21)-C(30)	84.8(3)
C(23)-N(22)-C(31)	121.4(7)
C(23)-N(22)-Se(21)	117.3(6)
C(31)-N(22)-Se(21)	121.3(5)
O(24)-C(23)-N(22)	123.5(7)
O(24)-C(23)-C(25)	126.1(7)
N(22)-C(23)-C(25)	110.4(7)
C(26)-C(25)-C(30)	119.0(8)
C(26)-C(25)-C(23)	125.4(8)
C(30)-C(25)-C(23)	115.5(7)
C(27)-C(26)-C(25)	119.5(8)
C(26)-C(27)-C(28)	121.2(8)
C(29)-C(28)-C(27)	120.3(8)
C(30)-C(29)-C(28)	117.9(8)

C(29)-C(30)-C(25)	121.9(7)
C(29)-C(30)-Se(21)	126.1(6)
C(25)-C(30)-Se(21)	111.9(6)
N(22)-C(31)-C(32)	110.3(8)
N(22)-C(31)-C(33)	108.2(7)
C(32)-C(31)-C(33)	115.3(8)
C(38)-C(33)-C(34)	118.1(8)
C(38)-C(33)-C(31)	123.3(9)
C(34)-C(33)-C(31)	118.6(8)
C(35)-C(34)-C(33)	121.2(10)
C(36)-C(35)-C(34)	119.9(10)
C(37)-C(36)-C(35)	119.9(9)
C(36)-C(37)-C(38)	120.7(9)
C(33)-C(38)-C(37)	120.1(9)
C(50)-Se(41)-N(42)	84.9(3)
C(43)-N(42)-C(51)	124.6(7)
C(43)-N(42)-Se(41)	116.6(6)
C(51)-N(42)-Se(41)	118.7(6)
O(44)-C(43)-N(42)	122.0(8)
O(44)-C(43)-C(45)	126.7(8)
N(42)-C(43)-C(45)	111.3(7)
C(46)-C(45)-C(50)	119.4(8)
C(46)-C(45)-C(43)	125.5(8)
C(50)-C(45)-C(43)	115.1(7)
C(45)-C(46)-C(47)	120.2(8)
C(46)-C(47)-C(48)	120.3(9)
C(49)-C(48)-C(47)	120.8(8)
C(48)-C(49)-C(50)	119.2(8)
C(49)-C(50)-C(45)	120.1(8)
C(49)-C(50)-Se(41)	128.0(6)
C(45)-C(50)-Se(41)	111.9(6)
N(42)-C(51)-C(53)	108.2(7)
N(42)-C(51)-C(52)	109.1(8)
C(53)-C(51)-C(52)	115.5(9)
C(58)-C(53)-C(54)	118.7(9)
C(58)-C(53)-C(51)	123.3(9)
C(54)-C(53)-C(51)	117.9(9)
C(55)-C(54)-C(53)	121.0(9)
C(54)-C(55)-C(56)	120.4(10)
C(57)-C(56)-C(55)	119.6(9)
C(56)-C(57)-C(58)	120.1(9)
C(53)-C(58)-C(57)	120.1(9)
N(62)-Se(61)-C(70)	85.8(3)
C(63)-N(62)-C(71)	123.9(7)
C(63)-N(62)-Se(61)	116.8(5)
C(71)-N(62)-Se(61)	118.8(5)
O(64)-C(63)-N(62)	124.9(7)
O(64)-C(63)-C(65)	125.1(7)
N(62)-C(63)-C(65)	110.0(6)

C(66)-C(65)-C(70)	120.2(8)
C(66)-C(65)-C(63)	123.9(7)
C(70)-C(65)-C(63)	115.8(7)
C(67)-C(66)-C(65)	117.8(8)
C(68)-C(67)-C(66)	121.2(9)
C(69)-C(68)-C(67)	121.2(9)
C(68)-C(69)-C(70)	118.5(8)
C(69)-C(70)-C(65)	121.1(8)
C(69)-C(70)-Se(61)	127.5(6)
C(65)-C(70)-Se(61)	111.5(6)
N(62)-C(71)-C(73)	110.2(7)
N(62)-C(71)-C(72)	111.1(8)
C(73)-C(71)-C(72)	114.8(8)
C(78)-C(73)-C(74)	117.4(8)
C(78)-C(73)-C(71)	119.3(8)
C(74)-C(73)-C(71)	123.2(8)
C(75)-C(74)-C(73)	121.2(9)
C(76)-C(75)-C(74)	119.3(10)
C(75)-C(76)-C(77)	121.1(10)
C(78)-C(77)-C(76)	119.1(9)
C(77)-C(78)-C(73)	121.8(9)
N(82)-Se(81)-C(90)	84.2(3)
C(83)-N(82)-C(91)	123.2(7)
C(83)-N(82)-Se(81)	117.4(6)
C(91)-N(82)-Se(81)	119.0(6)
O(84)-C(83)-N(82)	124.1(7)
O(84)-C(83)-C(85)	125.9(8)
N(82)-C(83)-C(85)	109.9(7)
C(90)-C(85)-C(86)	119.7(8)
C(90)-C(85)-C(83)	116.3(8)
C(86)-C(85)-C(83)	124.0(7)
C(87)-C(86)-C(85)	118.9(8)
C(86)-C(87)-C(88)	120.7(9)
C(87)-C(88)-C(89)	121.1(8)
C(88)-C(89)-C(90)	116.8(8)
C(85)-C(90)-C(89)	122.7(8)
C(85)-C(90)-Se(81)	112.2(6)
C(89)-C(90)-Se(81)	125.0(7)
N(82)-C(91)-C(93)	109.8(7)
N(82)-C(91)-C(92)	109.1(9)
C(93)-C(91)-C(92)	115.7(9)
C(98)-C(93)-C(94)	118.3(8)
C(98)-C(93)-C(91)	123.4(9)
C(94)-C(93)-C(91)	118.3(8)
C(95)-C(94)-C(93)	121.2(10)
C(96)-C(95)-C(94)	120.0(11)
C(95)-C(96)-C(97)	118.8(10)
C(96)-C(97)-C(98)	121.6(10)
C(93)-C(98)-C(97)	120.0(9)

Table S7. Torsion angles [°] for **25a**.

C(11)-N(2)-C(3)-O(4)	1.8(12)
Se(1)-N(2)-C(3)-O(4)	-176.7(6)
C(11)-N(2)-C(3)-C(5)	178.1(7)
Se(1)-N(2)-C(3)-C(5)	-0.5(9)
O(4)-C(3)-C(5)-C(10)	178.7(8)
N(2)-C(3)-C(5)-C(10)	2.5(10)
O(4)-C(3)-C(5)-C(6)	-2.9(13)
N(2)-C(3)-C(5)-C(6)	-179.1(7)
C(10)-C(5)-C(6)-C(7)	-0.7(13)
C(3)-C(5)-C(6)-C(7)	-179.0(8)
C(5)-C(6)-C(7)-C(8)	-0.2(13)
C(6)-C(7)-C(8)-C(9)	0.9(14)
C(7)-C(8)-C(9)-C(10)	-0.8(13)
C(6)-C(5)-C(10)-C(9)	0.9(13)
C(3)-C(5)-C(10)-C(9)	179.3(7)
C(6)-C(5)-C(10)-Se(1)	178.2(6)
C(3)-C(5)-C(10)-Se(1)	-3.3(9)
C(8)-C(9)-C(10)-C(5)	-0.1(13)
C(8)-C(9)-C(10)-Se(1)	-177.0(7)
N(2)-Se(1)-C(10)-C(5)	2.4(6)
N(2)-Se(1)-C(10)-C(9)	179.5(8)
C(3)-N(2)-C(11)-C(13)	-154.0(7)
Se(1)-N(2)-C(11)-C(13)	24.5(9)
C(3)-N(2)-C(11)-C(12)	79.0(9)
Se(1)-N(2)-C(11)-C(12)	-102.5(7)
N(2)-C(11)-C(13)-C(14)	87.1(9)
C(12)-C(11)-C(13)-C(14)	-148.5(8)
N(2)-C(11)-C(13)-C(18)	-89.6(9)
C(12)-C(11)-C(13)-C(18)	34.8(11)
C(18)-C(13)-C(14)-C(15)	1.4(13)
C(11)-C(13)-C(14)-C(15)	-175.4(8)
C(13)-C(14)-C(15)-C(16)	-1.2(14)
C(14)-C(15)-C(16)-C(17)	0.9(14)
C(15)-C(16)-C(17)-C(18)	-0.8(14)
C(16)-C(17)-C(18)-C(13)	0.9(13)
C(14)-C(13)-C(18)-C(17)	-1.2(12)
C(11)-C(13)-C(18)-C(17)	175.6(8)
C(30)-Se(21)-N(22)-C(23)	-1.3(6)
C(30)-Se(21)-N(22)-C(31)	179.5(7)
C(31)-N(22)-C(23)-O(24)	2.2(13)
Se(21)-N(22)-C(23)-O(24)	-177.0(6)
C(31)-N(22)-C(23)-C(25)	-178.8(8)
Se(21)-N(22)-C(23)-C(25)	2.1(9)
O(24)-C(23)-C(25)-C(26)	-0.7(14)
N(22)-C(23)-C(25)-C(26)	-179.8(8)
O(24)-C(23)-C(25)-C(30)	177.1(8)

N(22)-C(23)-C(25)-C(30)	-1.9(10)
C(30)-C(25)-C(26)-C(27)	0.9(13)
C(23)-C(25)-C(26)-C(27)	178.6(8)
C(25)-C(26)-C(27)-C(28)	-1.2(14)
C(26)-C(27)-C(28)-C(29)	0.8(15)
C(27)-C(28)-C(29)-C(30)	-0.1(13)
C(28)-C(29)-C(30)-C(25)	-0.2(13)
C(28)-C(29)-C(30)-Se(21)	-179.2(7)
C(26)-C(25)-C(30)-C(29)	-0.2(13)
C(23)-C(25)-C(30)-C(29)	-178.2(7)
C(26)-C(25)-C(30)-Se(21)	178.9(6)
C(23)-C(25)-C(30)-Se(21)	1.0(10)
N(22)-Se(21)-C(30)-C(29)	179.2(8)
N(22)-Se(21)-C(30)-C(25)	0.1(6)
C(23)-N(22)-C(31)-C(32)	86.4(10)
Se(21)-N(22)-C(31)-C(32)	-94.5(9)
C(23)-N(22)-C(31)-C(33)	-146.7(8)
Se(21)-N(22)-C(31)-C(33)	32.4(10)
N(22)-C(31)-C(33)-C(38)	-104.2(10)
C(32)-C(31)-C(33)-C(38)	19.7(13)
N(22)-C(31)-C(33)-C(34)	75.2(10)
C(32)-C(31)-C(33)-C(34)	-160.8(9)
C(38)-C(33)-C(34)-C(35)	3.3(14)
C(31)-C(33)-C(34)-C(35)	-176.2(9)
C(33)-C(34)-C(35)-C(36)	-1.5(16)
C(34)-C(35)-C(36)-C(37)	-0.9(17)
C(35)-C(36)-C(37)-C(38)	1.4(16)
C(34)-C(33)-C(38)-C(37)	-2.8(13)
C(31)-C(33)-C(38)-C(37)	176.7(9)
C(36)-C(37)-C(38)-C(33)	0.5(15)
C(50)-Se(41)-N(42)-C(43)	-3.6(7)
C(50)-Se(41)-N(42)-C(51)	177.6(8)
C(51)-N(42)-C(43)-O(44)	3.4(15)
Se(41)-N(42)-C(43)-O(44)	-175.3(7)
C(51)-N(42)-C(43)-C(45)	-176.7(9)
Se(41)-N(42)-C(43)-C(45)	4.6(10)
O(44)-C(43)-C(45)-C(46)	-3.2(16)
N(42)-C(43)-C(45)-C(46)	177.0(9)
O(44)-C(43)-C(45)-C(50)	176.7(9)
N(42)-C(43)-C(45)-C(50)	-3.1(11)
C(50)-C(45)-C(46)-C(47)	-1.7(14)
C(43)-C(45)-C(46)-C(47)	178.2(9)
C(45)-C(46)-C(47)-C(48)	-0.7(16)
C(46)-C(47)-C(48)-C(49)	3.2(16)
C(47)-C(48)-C(49)-C(50)	-3.1(14)
C(48)-C(49)-C(50)-C(45)	0.7(13)
C(48)-C(49)-C(50)-Se(41)	-177.6(7)
C(46)-C(45)-C(50)-C(49)	1.7(13)
C(43)-C(45)-C(50)-C(49)	-178.1(8)

C(46)-C(45)-C(50)-Se(41)	-179.8(7)
C(43)-C(45)-C(50)-Se(41)	0.4(10)
N(42)-Se(41)-C(50)-C(49)	-180.0(8)
N(42)-Se(41)-C(50)-C(45)	1.6(6)
C(43)-N(42)-C(51)-C(53)	-134.0(9)
Se(41)-N(42)-C(51)-C(53)	44.6(10)
C(43)-N(42)-C(51)-C(52)	99.6(12)
Se(41)-N(42)-C(51)-C(52)	-81.8(9)
N(42)-C(51)-C(53)-C(58)	-114.9(9)
C(52)-C(51)-C(53)-C(58)	7.7(13)
N(42)-C(51)-C(53)-C(54)	61.8(11)
C(52)-C(51)-C(53)-C(54)	-175.6(9)
C(58)-C(53)-C(54)-C(55)	0.5(14)
C(51)-C(53)-C(54)-C(55)	-176.3(9)
C(53)-C(54)-C(55)-C(56)	-0.4(16)
C(54)-C(55)-C(56)-C(57)	0.3(16)
C(55)-C(56)-C(57)-C(58)	-0.4(15)
C(54)-C(53)-C(58)-C(57)	-0.6(12)
C(51)-C(53)-C(58)-C(57)	176.0(8)
C(56)-C(57)-C(58)-C(53)	0.6(13)
C(70)-Se(61)-N(62)-C(63)	-2.6(6)
C(70)-Se(61)-N(62)-C(71)	-175.3(7)
C(71)-N(62)-C(63)-O(64)	-3.1(13)
Se(61)-N(62)-C(63)-O(64)	-175.3(6)
C(71)-N(62)-C(63)-C(65)	176.0(7)
Se(61)-N(62)-C(63)-C(65)	3.7(9)
O(64)-C(63)-C(65)-C(66)	-5.1(13)
N(62)-C(63)-C(65)-C(66)	175.9(8)
O(64)-C(63)-C(65)-C(70)	175.9(8)
N(62)-C(63)-C(65)-C(70)	-3.2(10)
C(70)-C(65)-C(66)-C(67)	-1.5(13)
C(63)-C(65)-C(66)-C(67)	179.4(8)
C(65)-C(66)-C(67)-C(68)	-0.4(14)
C(66)-C(67)-C(68)-C(69)	1.7(15)
C(67)-C(68)-C(69)-C(70)	-1.0(13)
C(68)-C(69)-C(70)-C(65)	-1.0(12)
C(68)-C(69)-C(70)-Se(61)	179.2(7)
C(66)-C(65)-C(70)-C(69)	2.3(12)
C(63)-C(65)-C(70)-C(69)	-178.6(7)
C(66)-C(65)-C(70)-Se(61)	-177.9(6)
C(63)-C(65)-C(70)-Se(61)	1.2(9)
N(62)-Se(61)-C(70)-C(69)	-179.5(8)
N(62)-Se(61)-C(70)-C(65)	0.6(6)
C(63)-N(62)-C(71)-C(73)	-109.3(9)
Se(61)-N(62)-C(71)-C(73)	62.8(9)
C(63)-N(62)-C(71)-C(72)	122.3(9)
Se(61)-N(62)-C(71)-C(72)	-65.6(9)
N(62)-C(71)-C(73)-C(78)	76.5(10)
C(72)-C(71)-C(73)-C(78)	-157.2(8)

N(62)-C(71)-C(73)-C(74)	-100.2(9)
C(72)-C(71)-C(73)-C(74)	26.1(12)
C(78)-C(73)-C(74)-C(75)	-0.2(13)
C(71)-C(73)-C(74)-C(75)	176.5(9)
C(73)-C(74)-C(75)-C(76)	0.7(14)
C(74)-C(75)-C(76)-C(77)	-0.1(15)
C(75)-C(76)-C(77)-C(78)	-0.9(16)
C(76)-C(77)-C(78)-C(73)	1.4(15)
C(74)-C(73)-C(78)-C(77)	-0.8(13)
C(71)-C(73)-C(78)-C(77)	-177.7(9)
C(90)-Se(81)-N(82)-C(83)	-2.7(7)
C(90)-Se(81)-N(82)-C(91)	-176.3(8)
C(91)-N(82)-C(83)-O(84)	-0.2(15)
Se(81)-N(82)-C(83)-O(84)	-173.5(7)
C(91)-N(82)-C(83)-C(85)	176.2(8)
Se(81)-N(82)-C(83)-C(85)	2.9(10)
O(84)-C(83)-C(85)-C(90)	174.9(8)
N(82)-C(83)-C(85)-C(90)	-1.5(11)
O(84)-C(83)-C(85)-C(86)	-4.0(14)
N(82)-C(83)-C(85)-C(86)	179.6(8)
C(90)-C(85)-C(86)-C(87)	-0.9(13)
C(83)-C(85)-C(86)-C(87)	178.0(8)
C(85)-C(86)-C(87)-C(88)	-0.6(14)
C(86)-C(87)-C(88)-C(89)	0.9(15)
C(87)-C(88)-C(89)-C(90)	0.3(14)
C(86)-C(85)-C(90)-C(89)	2.2(13)
C(83)-C(85)-C(90)-C(89)	-176.7(8)
C(86)-C(85)-C(90)-Se(81)	178.4(7)
C(83)-C(85)-C(90)-Se(81)	-0.5(10)
C(88)-C(89)-C(90)-C(85)	-1.9(13)
C(88)-C(89)-C(90)-Se(81)	-177.6(6)
C(83)-N(82)-C(91)-C(93)	-129.8(9)
Se(81)-N(82)-C(91)-C(93)	43.4(11)
C(83)-N(82)-C(91)-C(92)	102.5(12)
Se(81)-N(82)-C(91)-C(92)	-84.3(10)
N(82)-C(91)-C(93)-C(98)	-120.6(9)
C(92)-C(91)-C(93)-C(98)	3.4(14)
N(82)-C(91)-C(93)-C(94)	57.7(11)
C(92)-C(91)-C(93)-C(94)	-178.3(10)
C(98)-C(93)-C(94)-C(95)	1.1(13)
C(91)-C(93)-C(94)-C(95)	-177.3(8)
C(93)-C(94)-C(95)-C(96)	-1.3(15)
C(94)-C(95)-C(96)-C(97)	1.2(15)
C(95)-C(96)-C(97)-C(98)	-0.9(15)
C(94)-C(93)-C(98)-C(97)	-0.8(13)
C(91)-C(93)-C(98)-C(97)	177.5(8)
C(96)-C(97)-C(98)-C(93)	0.7(14)

Table S8. Hydrogen bonds for **25a** [Å and °].

D-H...A	d(D-H)	d(H...A)	d(D...A)	<(DHA)
C(9)-H(9)...O(4)#1	0.95	2.28	2.953(10)	127.3
C(29)-H(29)...O(44)#2	0.95	2.36	3.037(10)	128.1
C(49)-H(49)...O(24)	0.95	2.35	3.020(10)	127.1
C(69)-H(69)...O(84)	0.95	2.45	3.125(10)	127.8
C(89)-H(89)...O(64)#3	0.95	2.50	3.200(11)	130.6

Symmetry transformations used to generate equivalent atoms:.
#1 -x+1,y-1/2,-z+1/2 #2 x,y-1,z #3 x,y+1,z .
